# 53BP1-ACLY-SLBP-coordinated activation of replication-dependent histone biogenesis maintains genomic integrity

**DOI:** 10.1093/nar/gkab1300

**Published:** 2022-01-17

**Authors:** TingTing Wu, Semo Jun, Eun-Ji Choi, Jiao Sun, Eun-Bi Yang, Hyun-Seo Lee, Sang-Yong Kim, Naima Ahmed Fahmi, Qibing Jiang, Wei Zhang, Jeongsik Yong, Jung-Hee Lee, Ho Jin You

**Affiliations:** DNA Damage Response Network Center; Department of Pharmacology; DNA Damage Response Network Center; Department of Pharmacology; DNA Damage Response Network Center; Department of Cellular and Molecular Medicine; Department of Computer Science, University of Central Florida, Orlando, FL 32816, USA; DNA Damage Response Network Center; Department of Cellular and Molecular Medicine; DNA Damage Response Network Center; Division of Endocrinology, Chosun University School of medicine, 375 Seosuk-dong, Gwangju 61452, Republic of Korea; Division of Endocrinology, Chosun University School of medicine, 375 Seosuk-dong, Gwangju 61452, Republic of Korea; Division of Endocrinology, Chosun University School of medicine, 375 Seosuk-dong, Gwangju 61452, Republic of Korea; Division of Endocrinology, Chosun University School of medicine, 375 Seosuk-dong, Gwangju 61452, Republic of Korea; Department of Biochemistry, Molecular Biology and Biophysics, University of Minnesota Twin Cities, Minneapolis, MN 55455, USA; DNA Damage Response Network Center; Department of Cellular and Molecular Medicine; DNA Damage Response Network Center; Department of Pharmacology

## Abstract

p53-binding protein 1 (53BP1) regulates the DNA double-strand break (DSB) repair pathway and maintains genomic integrity. Here we found that 53BP1 functions as a molecular scaffold for the nucleoside diphosphate kinase-mediated phosphorylation of ATP-citrate lyase (ACLY) which enhances the ACLY activity. This functional association is critical for promoting global histone acetylation and subsequent transcriptome-wide alterations in gene expression. Specifically, expression of a replication-dependent histone biogenesis factor, stem-loop binding protein (SLBP), is dependent upon 53BP1-ACLY-controlled acetylation at the SLBP promoter. This chain of regulation events carried out by 53BP1, ACLY, and SLBP is crucial for both quantitative and qualitative histone biogenesis as well as for the preservation of genomic integrity. Collectively, our findings reveal a previously unknown role for 53BP1 in coordinating replication-dependent histone biogenesis and highlight a DNA repair-independent function in the maintenance of genomic stability through a regulatory network that includes ACLY and SLBP.

## INTRODUCTION

p53-binding protein 1 (53BP1) has been studied extensively for its role in modulating both the nonhomologous end joining (NHEJ) and homologous recombination (HR) pathways necessary for DNA double-strand break (DSB) repair. When DSBs occur, chromatin surrounding the lesions is ubiquitinated by the E3 ligases RNF8 and RNF168. 53BP1 is then recruited to these sites and binds both ubiquitinylated H2AK15 and di-methylated histone H4K20 (1,2). Recruitment of 53BP1 is important for maintaining genomic integrity because it promotes the classical NHEJ pathway over the error-prone alternative end joining repair pathway (3,4) and suppresses hyper-resectioning to minimize mutations that arise during homology-directed repair (5). This is accomplished by the 53BP1/RIF1/REV7 complex and a downstream effector, the shieldin complex, which restrains DNA end resection (6–8). However, the same process is deleterious in cancers caused by a BRCA1 dysfunction. The dysfunctions associated with a BRCA1 deficiency, including genomic instability, tumorigenesis, HR defects, and high sensitivity to poly(ADP-ribose) polymerase (PARP) inhibitor, are restored when 53BP1 is absent (6–12). When 53BP1 promotes NHEJ and inhibits HR in cells with a BRCA1 mutation, massive chromosomal rearrangements and genomic instability drive tumorigenesis (13).

53BP1 also functions as a tumor suppressor, shown in cancer-prone 53BP1^−^/^−^ mouse models (14–16). 53BP1-deficient cells have various types of chromosomal instabilities, including structural chromosomal aberrations and aneuploidy, which are inconsistent with DSB repair functions (14–17). For this reason, it has been suggested that 53BP1 plays additional roles in suppressing chromosomal instability and counteracting tumorigenesis. Several lines of evidence suggest that 53BP1 is involved in proper mitotic progression (18–22) and contributes to protecting and/or restarting stalled replication forks after replication stress (23–25). These functions appear to be independent of its well-understood role in DSB repair, but details are lacking.

ATP citrate lyase (ACLY) converts mitochondria-derived citrate into acetyl-CoA, which is the building block for de novo lipid synthesis (26). ACLY is widely expressed in many tissues and its activity is elevated in lipogenic tissues including fat, liver, and lactating mammary glands (27). ACLY also exists in the nucleus and functions in histone acetylation, thus regulating gene expression (28). The enzymatic activity of ACLY is regulated by autophosphorylation or phosphorylation by Akt, nucleoside diphosphate kinase (NDPK), or cyclic AMP-dependent protein kinase (27). Particularly, NDPK phosphorylates ACLY at histidine 760 (H760), which serves as a catalytic phosphate acceptor. The phosphorylation of ACLY H760 allows the formation of a citryl-CoA intermediate that is cleaved into acetyl-CoA and oxaloacetate, and thus the alanine mutation of H760 (H760A) is known to impair the ACLY enzymatic activity (26,29–32).

In this study, we discovered a previously uncharacterized molecular link between 53BP1 and ACLY, and their regulatory role on histone modifications. Further characterization of the interaction of 53BP1 with ACLY revealed an unknown regulatory cascade of 53BP1-ACLY on the regulation of histone biogenesis and a DNA repair-independent molecular mechanism of genome maintenance.

## MATERIAL AND METHODS

### Cell lines and culture conditions

Dulbecco's modified Eagle's medium (DMEM) was used to culture each of the following cell lines: human cervix adenocarcinoma HeLa cells, human osteosarcoma U2OS cells, human embryonic kidney HEK293T cells, 53BP1 wild-type and 53BP1 knockout mouse embryonic fibroblasts, 53BP1^+/+^ MEF and 53BP1^−/−^ MEF cells. Human breast cancer cells (MCF-7, BT549, T47D, and H1937), human lung cancer cells (A549, H1299, and H460), human colon cancer SW480, and human prostate cancer PC3 cells were cultured in RPMI-1640 medium. Human colon cancer HCT116 (p53^+/+^ and p53^−/−^) cells were grown in Iscove's modified Dulbecco's medium. MDA-MD-231 and MDA-MB-436 (human breast cancer) and MRC5 (normal lung) cells were maintained in modified Eagle's medium. In all cases, the media was supplemented with 10% heat-inactivated fetal bovine serum, 100 units/ml penicillin, and 100 mg/ml streptomycin sulfate (Invitrogen, Carlsbad, CA, USA). For primary MEF cells, 1 × MEM non-essential amino acid solution (Sigma-Aldrich, St. Louis, MO, USA) was included. All cells were maintained in a humidified incubator at 37°C under 5% CO_2_. Upon reaching 70–80% confluency, cells were digested with 0.5% trypsin-EDTA before passaging. Cells in exponential growth were harvested for subsequent experiments. To determine glucose-induced histone acetylation, the same number of 53BP1^+/+^ and 53BP1^−/−^ MEFs were first seeded and cultured in DMEM culture media for 24 h. And then cells were cultured in glucose-free DMEM media (Invitrogen) supplemented with or without 25 mM glucose for 48 h.

### Mouse

All animal procedures were reviewed and approved by the Institutional Animal Welfare and Use Committee of Chosun University School of Medicine. B6129SF1/J (Stock No: 101043) and B6;129-*Trp53bp1^tm1Jc^*/J (Stock No: 006495) mice were obtained from the Jackson Laboratory. A pregnant female mouse (13.5 days) was sacrificed by cervical dislocation. Embryos were separated from the placenta and washed three times with PBS, after which the head and internal organs was removed from each. The embryos were then minced using a sterile razor blade for several minutes until further reduced in size. The minced tissue was transferred to a 15 mL tube and incubated at room temperature for 5 min, after which the supernatant was removed and 1–2 mL trypsin mixture (20% collagenase: 0.25% trypsin EDTA: free media at 1:4:5) was added. The cells were then dissociated from the tissues by vigorous pipetting and incubated in a 37°C water bath for 30 min. After centrifugation at 1,500 rpm for 10 min and removal of the supernatant, cell pellets were resuspended in DMEM media. These cells were designated passage 0 (P0). After growth to 80–90% confluence over the course of 2–3 days, cells were frozen for future use. For immortalization of MEF cells, we used SV40 T antigen. The primary MEF cells (P2-4) were transfected with 2 μg T antigen expression vector using Lipofectamine 2000 (Invitrogen) for 48 h. When cells had just reached confluence, they were split into 100 mm culture dishes and were designated P1. To achieve immortalized cells, low density (1/10) and high density (1/4) cultures were split at least 5 times. By P6, normal growth indicated that the cells had become immortalized.

### Plasmid constructs and transfection

**Figure 1.**
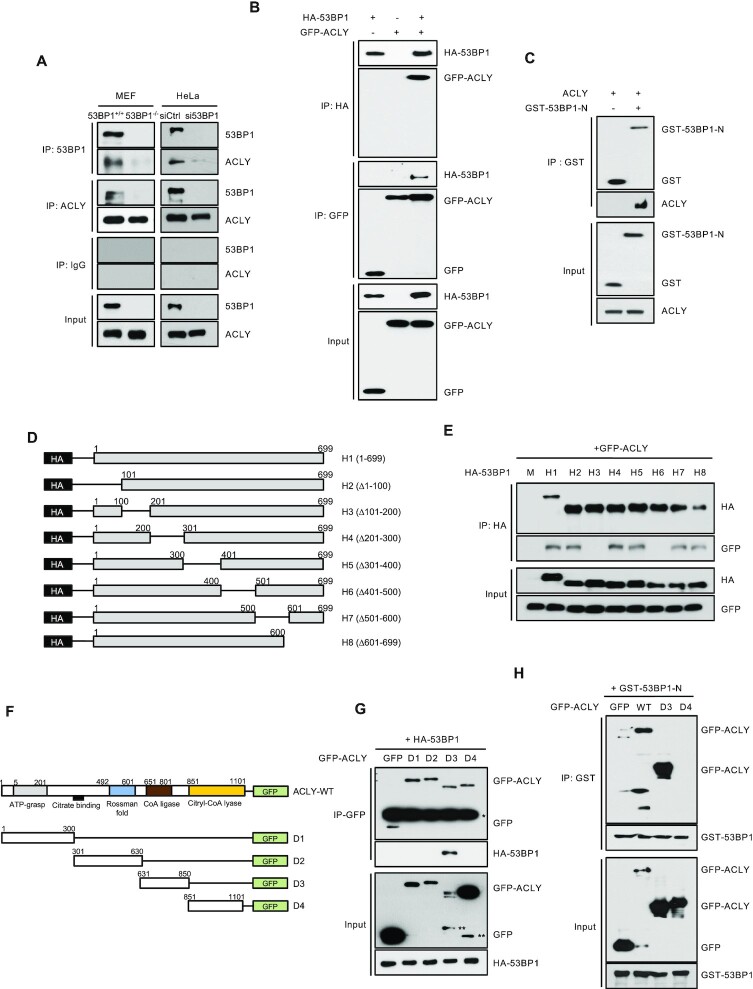
53BP1 interacts with ACLY and promotes ACLY activity. **(A)** Immunoprecipitations (IPs) with anti-53BP1, anti-ACLY, or control IgG antibodies were carried out using extracts from 53BP1^+^/^+^ or 53BP1^−/−^ MEFs (left) and control or 53BP1-depleted HeLa cells (right). Western blots were conducted using the indicated antibodies. **(B)** Co-IPs and western blot analysis of overexpressed HA-53BP1 and GFP-ACLY in HEK293T cells. Antibodies used for IPs and western blots are indicated. **(C)** A GST pulldown assay was carried out using GST or GST-tagged N-terminus of the 53BP1 fusion protein (GST-53BP1-N) and recombinant ACLY protein. The *in vitro* interaction of ACLY with GST-53BP1-N was validated by western blotting using an anti-ACLY antibody. Note that GST-53BP1-N migrates to a position where a dimer would be expected. **(D)** A schematic of the HA-tagged N-terminal S/T-Q motif of 53BP1 (H1) and seven deletion mutants (H2-H8) is shown. **(E)** HEK293T cells were co-transfected with the indicated HA-tagged 53BP1 constructs along with the GFP-tagged ACLY construct. Cell lysates were then subjected to IPs and western blots using indicated antibodies. **(F)** A schematic of GFP-ACLY (full length) and a series of deletion mutants is shown. **(G)** Co-IPs and western blot analyses of the interactions between GFP-ACLY deletion mutants and HA-53BP1 in HEK293T cells. **(H)** A GST pulldown assay was carried out using recombinant GST-53BP1-N and total cell lysates from WT GFP-ACLY or GFP-ACLY deletion mutants (D3 and D4) transfected HEK293T cells.

The human wild-type 53BP1 constructs (pcDNA-HA-53BP1) and ATM phosphomutant of 53BP1 (53BP1-20AQ) were a gift from Prof. Stanley Fields (Department of Medicine, University of Washington) and Dr. Simon J. Boulton (DSB Repair Metabolism Lab., The Francis Crick Institute), respectively. To generate N-terminal (amino acids 1–699, H), C-terminal (amino acids 1627–1972), and N-terminal serial deletion (H2-H8) mutants of 53BP1, each region was PCR amplified from pcDNA-HA-53BP1 and the products were cloned into a pcDNA-HA vector. To produce the full-length and individual domain constructs of ACLY, each region of the ACLY gene (D1-D4) was amplified from human HeLa cDNA, and the PCR products were cloned into pEGFP-N1 (Figure 1D; all protein sequence numbering was based on NP_001087.2). To create an expression vector encoding the ACLY H760A mutant, we performed mutagenesis using the GENEART^®^ Site-Directed Mutagenesis System (Invitrogen) according to the manufacturer's instructions. The pCMV6-Entry-SLBP construct was obtained from OriGene (OriGene Technologies, Inc. Rockville, MD, USA). Transfection of the cells with expression plasmids was performed with the use of the Lipofectamine 2000 reagent (Invitrogen). The primer sequences used in these experiments were listed in Supplementary Table S5. All constructs were confirmed by automated DNA sequencing.

### RNA-seq analysis

To evaluate the transcriptome of the 53BP1 knockout at the nucleotide level, we performed two biological repeats of RNA-seq experiments using poly (A^+^) RNA isolated from 53BP1^+/+^ and 53BP1^−/−^ MEF cells. The short reads were aligned to the mm10 reference genome provided by TopHat (33) with up to two mismatches allowed. The unmapped reads were first trimmed to remove poly-A/T tails (repeats of [A/N]s or [T/N]s) from read ends/starts and then aligned to the reference genome. Only the reads with at least 30 bp in both ends were retained after trimming. To identify the differentially expressed genes, Kallisto was first applied to quantify the gene expression with RefSeq annotation (34,35). The genes are considered ‘expressed’ in 53BP1^+/+^ and 53BP1^−/−^ if the CPM (Counts per million) is larger than 1. Then DESeq2 was applied to identify the differentially expressed genes between 53BP1^+/+^ and 53BP1^−/−^ based on the union of the two sets of genes that are expressed in at least one of the cell lines (36). Fold-change and FDR cutoffs were 2 and 0.1 respectively. RNA-seq data generated for this study is available at the NCBI BioProject database (https://www.ncbi.nlm.nih.gov/bioproject/) under accession number PRJNA496219.

### ACLY activity assay

ACLY activity was measured using the malate dehydrogenase coupled method as described previously (37). Cells were lysed in extraction buffer (10 mM MgCl_2_, 100 mM Tris-HCl pH 8.5, 20 mM citrate, 0.33 mM CoASH, 0.14 mM NADH, 10 mM DTT, 2.5 mM ATP and 3.3 U/mL MDH) at 37°C for 10 min. ACLY activity was measured every 5 min over the course of more than 2 h using a spectrophotometer.

### Acetyl-CoA measurements

Cells were washed with cold PBS, scraped, lysed in cell lysis buffer (20 mM Tris (pH7.5), 150 mM NaCl, 1 mM EDTA, 1% Triton X-100, 2.5 mM sodium pyrophosphate, 1 mM Glycerolphosphate, 1 μg/ml leupeptin and 1 mM PMSF) and proteins were precipitated with 2.5 μM perichloric acids/mg. After centrifugation, supernatant was neutralized with 3M potassium bicarbonate solution until pH 6–8 range and centrifuged for removing precipitates. The supernatants measured acetyl-CoA concentration in triplicate by using an Acetyl-CoA assay kit (Sigma MAK039) according to manufacturer's instructions. Fluorescence was detected by VARIOSKAN LUX microplate reader (excitation/emission: 535/587 nm, Thermoscientific).

### Real-time quantitative PCR (RT-qPCR) analysis

Total RNA was isolated from cells using the Trizol Reagent (Invitrogen) according to the manufacturer's instructions. RNA samples were subsequently treated with DNase I (Thermo scientific) according to the manufacturer's instructions. 1 μg of Total or polyA^+^ RNA was reverse transcribed using Superscript III reverse transcriptase (Invitrogen) and either random hexamers (Macrogen, Korea) or oligo-dT primers (Macrogen). Real-time quantitative PCR was performed with SYBR Premix Ex Taq (Clontech, Mountain View, CA, USA) on a CFX96 (Bio‐Rad, Hercules, CA, USA) using the following program: 94°C for 2 min followed by 40 cycles of 94°C for 5 s, 60°C for 10 s, and 72°C for 20 s. Each reaction was performed in triplicate, and results of three independent experiments were used for statistical analysis. Relative mRNA expression levels were normalized against GAPDH mRNA expression using the ΔΔC(t) method. For the absolute quantification of histone mRNA, 1 μg of RNA was reverse transcribed using Superscript III reverse transcriptase (Invitrogen) and either random hexamers or oligo-dT primers. A standard curve was generated based on a serial dilution of PCR fragments were cloned using the *pGEM T easy vector* (Promega, Madison, WI, USA). Using the standard curve, the copy number of Histone mRNA was measured. Data are presented as means ± standard error of the *mean* (*s.d*.). Statistical comparisons were carried out using an unpaired *t*-test and values of *P* < 0.05 were considered to be statistically significant. The primer sequences used in these experiments were listed in Supplementary Tables S5 and S6.

### siRNA sequences and transfection

For knockdown of 53BP1, ACLY, SLBP, NDPK, Cdc7, Cen2, and Mcm5, HeLa cells and MEFs were transiently transfected with siRNA using lipofectamine RNAiMax (Invitrogen). The sequences of siRNAs used in these experiments were listed in Supplementary Table S7.

### Immunoprecipitation assay and western blotting

Cells were lysed in RIPA buffer (50 mM Tris–HCl pH 7.5, 150 mM NaCl, 1% NP-40, 0.5% sodium deoxycholate, 0.1% sodium dodecyl sulfate) containing protease inhibitors (Roche Diagnostic Corp.). Snap-frozen tissues (20–50 mg) were homogenized using a BioMasher (Nippi, Incorporated Protein Engineering Office, Japan) and added to RIPA Buffer. After centrifugation at 13,000 rpm for 30 min at 4°C, the supernatants were collected. Equal amounts of each extract were separated by 6–15% SDS–PAGE followed by electrotransfer onto a polyvinylidene difluoride membrane (Millipore, Bedford, MA, USA). The membranes were blocked for 1 h with TBS-t (10 mM Tris-HCl pH 7.4, 150 mM NaCl, and 0.1% Tween-20) containing 5% skim milk and then incubated with primary antibodies overnight at 4°C. The blots were washed four times for 15 min each with TBS-t and then incubated for 2 h with peroxidase-conjugated secondary antibodies. The membranes were washed four more times and developed using an enhanced chemiluminescence detection system (ECL; GE Healthcare, Buckinghamshire, UK). Protein was quantified using Scion Image software (Scion Corp., Fredrick, MD). For the immunoprecipitation assays, cell lysates were pre-cleared with Protein G Sepharose beads (GE Healthcare) prior to adding antibody. After pelleting the Protein G Sepharose by centrifugation, the supernatant was incubated at 4 °C overnight with the appropriate antibodies. Fresh Protein G Sepharose beads were added and the reaction mixture was incubated overnight at 4°C with rotation. The beads were washed three times in RIPA buffer without protease inhibitors, resuspended in SDS sample buffer, and boiled for 5 min. The samples were then analyzed by Western blotting using the appropriate antibodies. Western blot analysis we performed were repeated 3 times. The antibodies used in these experiments were listed in Supplementary Table S8.

### Construction of GST fusion proteins and *in vitro* binding assays

*E. coli* BL21 harboring the GST-53BP1-N-terminal expression construct was grown in Luria broth (LB) supplemented with 100 μg/ml ampicillin and incubated overnight at 37°C with shaking at 150 rpm. Fresh LB (20 ml) containing 100 μg/ml ampicillin was inoculated with 40 ul of pre-cultured cells and incubated at 37°C with shaking at 200 rpm until the culture reached an OD_600_ of 0.8. *E. coli* was then induced with 1 mM IPTG and incubated at 37°C with shaking at 200 rpm for 3 h. Cells were harvested and lysed in PBS buffer. Expression of GST-53BP1 was evaluated by 10% SDS-PAGE and visualized using Coomassie blue staining. For the *in vitro* binding assay, either GST alone or GST-tagged human 53BP1 N-terminal fragments were immobilized onto Glutathione Sepharose 4B beads (GE Healthcare) and incubated with 200 ng purified ACLY protein (Sino Biological Inc., Beijing, China) or ACLY-expressed cell lysates in 0.5 ml binding buffer (20 mM Tris-HCl, pH 7.5, 150 mM NaCl, 0.5% NP40, 1 mM EDTA, 1 mM DTT, and 10% glycerol) at 4 °C for 90 min. The GST beads were washed five times with NTEN buffer (0.5% NP-40, 20 mM Tris-HCl pH 7.4, 1 mM EDTA and 300 mM NaCl), and bound proteins were separated by SDS-PAGE and analyzed by Western blotting using the anti-ACLY antibody (Cell Signaling Technology, Danvers, MA, USA, #4332).

### DNA Fiber assay

To measure the replication elongation rate, cells were labeled with 500 μM 5-chloro-2′-deoxyuridine (CldU) for 40 min and chased with 500 μM 5-iodo-2′-deoxyuridine (IdU) for 40 min. For DNA replication fork restart analysis, cells were labeled with 500 μM CldU for 20 min, followed by exposure to 5 mM HU (hydroxyurea) for 2 h, and chased with 500 μM IdU for 1h. After labeled cells were harvested, cell lysates were prepared at a concentration of 7.5 × 10^5^ cells/ml in PBS. Two μl of cell lysate was dropped onto a cover slide and air-dried for 5 min or until the volume of the drop was greatly reduced but not completely dry, and then 7 μl lysis solution (50 mM EDTA, 0.5% SDS, 200 mM Tris-HCl pH7.5) was added on top of the lysate. The air-dried cover slide was fixed with methanol:acetic acid (at 3:1) for 10 min. DNA-stretched coverslips were denatured (2.5 N HCl for 45 min), neutralized (0.1 M sodium borate) and blocked (5% BSA and 0.5% Tween 20 in PBS) for 30 min. IdU and CldU tracts were stained by first binding with primary antibodies [mouse α-BrdU (Sigma-Aldrich) at 1:25 dilution for IdU and rat α-BrdU (Sigma-Aldrich) at 1:400 dilution for CldU] and then with fluorescently labeled secondary antibodies [anti-mouse IgG conjugated with AlexaFluor 488 (Invitrogen) for IdU and Cy3-conjugated anti-rat IgG (Invitrogen) for CldU]. Fibers were visualized under a confocal microscope (Zeiss LSM 510 Meta) and analyzed using Zeiss microscopic imaging software ZEN (Carl Zeiss, Oberkochen, Germany).

### Cell cycle analysis

Trypsinized cells were washed twice with PBS and fixed in 70% ethanol. The fixed cells were washed with PBS, incubated with 10 mg/ml RNase A (Thermo Scientific) at 37°C for 30 min, stained with Propidium Iodide (PI, 50 mg/ml), and analyzed on a FACSCalibur flow cytometer (BD Biosciences, San Jose, CA, USA). The percentage of cells in each phase of the cell cycle was determined using the Cell Quest Pro software (BD Biosciences). For S-phase progression studies, the cultures were incubated in media containing 50 ng/ml aphidicolin (Sigma-Aldrich) for G1 arrest. After 24 h, the media was replaced with fresh growth medium and 50 ng/ml colcemid (Sigma-Aldrich) for G2/M arrest. For cell cycle synchronization study, the cells were incubated in DMEM medium with 2 mM thymidine (Sigma-Aldrich) for 18 h. Cells were washed twice with PBS and released into fresh growth medium. After 9 h, cells were incubated in DMEM medium with 2 mM thymidine (Sigma-Aldrich) for 16 h. G1/S arrested cells were released into fresh medium were processed for FACS analysis.

### EdU incorporation assay

The EdU incorporation assay was performed with Click-iT® Plus EdU Imaging Kits (Molecular Probes, Carlsbad, CA, USA). Briefly, cells were pulsed with 10 μM 5-ethynyl-2′-deoxyuridine (EdU) for 4 h. Cells were fixed with 4% paraformaldehyde for 15 mins and subsequently washed twice with 3% BSA in PBS, followed by permeabilization with 0.5% Triton X-100 for 20 min at room temperature. After washing using 3% BSA, each slide was incubated with Click-iT Plus reaction cocktails including Alexa Flur 488 for 30 min at room temperature. For nuclear staining, cells were incubated with 5 μg/ml Hoechst 33342 solution for 30 min at room temperature. Numbers of EdU-positive and Hoechst-positive cells were analyzed using an IN Cell Analyzer 2500 HS (GE Healthcare).

### Measurement of new histone supply

To synchronize in G1/S phase, double thymidine block was accomplished by growing cells in the medium containing 2 mM thymidine (Sigma-Aldrich) for 18 h, released into fresh media without thymidine for 9 h. The cells were then reincubated in the medium containing 2 mM thymidine for an additional 17 h. Next, cells were released into fresh media for 2 h for synchronization in S-phase. The synchronized cells in S-phase were lysed in RIPA buffer, and total proteins were analyzed by western blotting using H3 and H4K12ac antibodies.

### Chromatin immunoprecipitation (ChIP) assay

ChIP was performed using a commercially available SimpleChIP Assay Kit (Cell Signaling Technology) according to the manufacturer's instructions. DNA-bound protein was immunoprecipitated using an anti-acetyl-Histone H3 antibody (*Millipore*) or rabbit IgG (Active Motif, Rixensart, Belgium) as a negative control. For quantification of co-precipitated DNA, Real-time qPCR were amplified using primers that bind to the promoter region of each gene with SYBR Premix Ex Taq (Clontech) on a CFX96 (Bio‐Rad) using the following program: 94°C for 2 min followed by 40 cycles of 94°C for 5 s, 60°C for 10 s, and 72°C for 20 s. The enrichments were calculated using the percent input method that is the signals obtained from the ChIP samples are divided by the signals obtained from the input samples. Each reaction was performed in triplicate, and results of three independent experiments were used for statistical analysis. The primer sequences used in these experiments were listed in Supplementary Table S5.

### Cell growth assay

Cells were harvested by trypsinization, counted on a Countess automated cell counter (Invitrogen), and plated at 1,500 cells per well in 96 tissue culture plates with 10 replicates. Photomicrographs were taken every 24 h using an Incucyte live cell imager (Essen Biosciences, Ann Arbor, MI, USA) and the confluence of cultures was measured using Incucyte software (Essen Biosciences) over 5 days in culture.

### Chromosomal aberration analysis

After transfection with SLBP siRNAs, cells were treated with 2 mM HU for 24 h. Cells were washed in PBS and then treated with 100 ng/ml colcemid (Sigma-Aldrich) for 1 h at 37 °C. Cells were harvested by trypsinization, incubated in 75 mM KCl for 20 min at 37°C and fixed in a methanol/acetic acid (3:1) solution. The fixed suspension was dropped onto slides to obtain chromosome spreads and air-dried overnight. The slide was mounted in Vectashield with DAPI (Vector Laboratories). The metaphase images were captured using confocal microscope (Zeiss LSM 510 Meta; Carl Zeiss) and visible chromatid breaks/gap were counted. At least 50 chromosomes were analyzed, and representative images were shown.

### Comparative Genomic Hybridization (CGH) array and data analysis

Genomic DNA from 53BP1^+/+^ MEF, SLBP-depleted 53BP1^+/+^ MEF, 53BP1^−/−^ MEF, and 53BP1^−/−^ MEF cells stably expressing Flag-SLBP was isolated using an AccuPrep^®^ Genomic DNA Extraction kit (Bioneer, Daejeon, South Korea) according to the manufacturer's instructions. Array CGH analysis was performed using a Mouse CGH 1 × 1M Array (Agilent Technologies, Santa Clara, CA, USA). Briefly, mouse genomic DNA (0.1 μg) from 53BP1^+/+^ MEF cells was labelled with Cy3-dCTP (as reference DNA). Mouse genomic DNA (0.1 μg) from 53BP1^+/+^ MEF cells stably expressing shSLBP1, 53BP1^−/−^ MEF cells, or 53BP1^−/−^ MEF cells stably expressing Flag-SLBP was labeled with Cy5-dCTP (as test DNA). The reference and test DNAs were cohybridize to the arrays, together with herring sperm DNA and washed. The hybridized array was scanned using Agilent's DNA microarray scanner and Feature Extraction Software (Agilent Technology) was used to process the images. The log_2_ ratio of the fluorescence intensities of test to reference was calculated from background-subtracted median intensity values. A test:reference log_2_ ratio of zero implies no copy number change at that clone, positive and negative values indicates gain and loss of copy number, respectively.

### Statistical analysis

Data analyses were conducted using GraphPad Prism software and Microsoft Excel where they were applicable. Differences between two independent groups were tested with two-tailed paired Student′s *t*-test. For the nonparametric statistical test, Mann-Whitney test was used. *p* value of less than 0.01 was considered statistically significant and *p* values were indicated by asterisks as followed: **P < 0.01 and n.s. = nonsignificant. Error bars represent standard deviation (SD) of triplicate experiments.

## RESULTS

### ACLY is a newly identified interactor of 53BP1.

53BP1 has functional domains that interact with numerous DSB-responsive proteins. Specifically, the clustered Ser/Thr-Gln sites (S/T-Q motifs; aa 1–699) and the tBRCT domain (aa 1661–1972) are known to bind multiple proteins (Supplementary Figure S1A) (13,38). To explore the biological function of 53BP1, we first determined the interactome of 53BP1 using yeast two-hybrid screening with either the S/T-Q motifs or the tBRCT domain as a bait. This screening confirmed the known tBRCT domain that binds p53. Surprisingly, we also identified novel interactors of the S/T-Q motif. One interactor of particular interest is ATP-citrate lyase (ACLY) (Supplementary Figure S1B-E), as it localizes to both the nucleus and cytosol and catalyzes the formation of glucose-derived acetyl-CoA from citrate. Hence, it plays an important role in *de novo* lipid synthesis and nuclear histone acetylation (27,28,39,40). To confirm this interaction, co-immunoprecipitation (co-IP) and western blotting experiments were performed using antibodies against 53BP1 and ACLY. As shown in Figure 1A, 53BP1 and ACLY co-immunoprecipitated together from cell extracts of both mouse embryonic fibroblasts (MEFs) and HeLa cells. The interaction between ACLY and 53BP1 was further validated; ectopic overexpression of GFP-ACLY and HA-53BP1 or HA-53BP1-N (aa 1–699; N-terminal S/T-Q motif) followed by co-IP and western blots confirmed the yeast two hybrid screening results (Figure 1B and Supplementary Figure S1F and G). This interaction between 53BP1 and ACLY was not affected by serum or glucose starvation (Supplementary Figure S1H). The direct interaction between 53BP1 and ACLY was confirmed using purified recombinant GST-53BP1-N and ACLY in a GST-pulldown experiment performed under cell-free conditions (Figure 1C).

To map the region of 53BP1-N involved with binding to ACLY, we generated HA-tagged constructs of eight different deletion mutants of 53BP1-N (Figure 1D) and used them for co-immunoprecipitation experiments with ACLY. ACLY co-immunoprecipitated with all of the tested deletion mutants except the 53BP1 fragments 101–200 (53BP1-H3) and 401–500 (53BP1-H6), suggesting that these regions are important for the interaction with ACLY (Figure 1E). A further dissection of this interaction using overexpression of various GFP-tagged ACLY deletion mutants (Figure 1F) and HA-53BP1 revealed the CoA ligase-containing region (residues 631–850; D3) of ACLY as the binding region for 53BP1 (Figure 1G). A GST pull-down experiment using GST-53BP1-N also supports the interaction between 53BP1-N and ACLY-D3 (Figure 1H).

### 53BP1 positively coordinates the ACLY activity.

**Figure 2.**
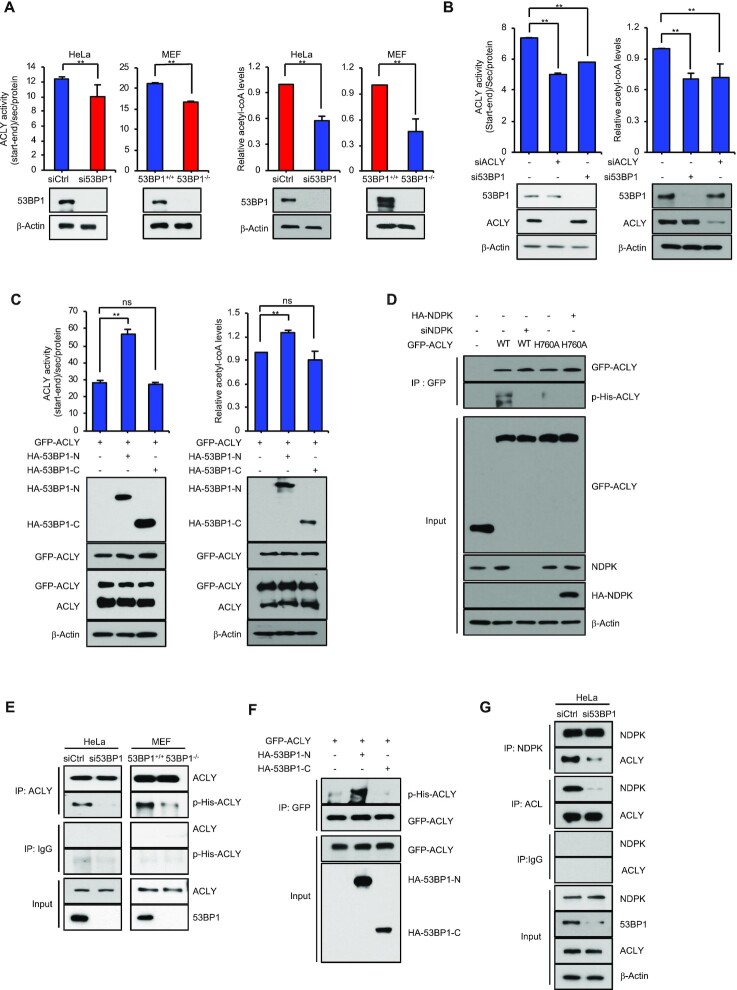
53BP1 positively regulates the ACLY activity by promoting the interaction between ACLY and NDPK. **(A)** The ACLY enzyme activity and the level of acetyl-CoA were measured in whole cell extracts made of control siRNA- and 53BP1 siRNA-transfected HeLa cells (left) or 53BP1^+/+^ and 53BP1^−/−^ MEFs (right). The results are shown as the mean ± SD (n = 3), ** *P* < 0.01, Student's *t*-test. (**B**) The ACLY enzyme activity and the level of acetyl-CoA were measured in whole cell extracts made of control siRNA-, ACLY siRNA-, and 53BP1 siRNA-transfected HeLa cells. The results are shown as the mean ± SD (n = 3), ** *P* < 0.01, Student's *t*-test. **(C)** HA-tagged N-terminus (HA-53BP1-N) or C-terminus (HA-53BP1-C) of 53BP1 was expressed in HEK293T cells along with GFP-ACLY. The ACLY enzyme activity and the level of acetyl-CoA were then measured after 48h. The results are shown as the mean ± SD (n = 3), ^∗∗^*P* < 0.01. ns, not significant, Student's *t*-test. **(D)** HA-53BP1, WT GFP-ACLY, GFP-ACLY H760A, HA-NDPK, and NDPK siRNA were transfected into HeLa cells with the indicated combinations. Co-IPs using the anti-GFP antibody were performed and the following western blot analyses were done using anti-ACLY or anti-phospho-histidine antibodies. (**E**) ACLY was immunoprecipitated from control or 53BP1 siRNA-transfected HeLa cells (left) and 53BP1^+/+^ or 53BP1^−/−^ MEF cell extracts (right). Phosphorylation of ACLY was analyzed using an anti-phospho-histidine antibody. (**F**) HA-53BP1-N or HA-53BP1-C was overexpressed in HEK293T cells along with GFP-ACLY. Co-IPs using an anti-GFP antibody was performed and analyzed by western blots using anti-ACLY or anti-phospho-histidine antibodies. **(G)** HeLa cells were transfected with control or 53BP1 siRNA for 48 h. IPs were conducted using an anti-NDPK antibody or an anti-ACLY antibody and western blot analyses were done using the indicated antibodies.

To investigate the physiological relevance of the 53BP1-ACLY interaction, we measured the ACLY activity and the amount of acetyl-CoA in the presence or absence of 53BP1. In HeLa cells, with the knockdown of 53BP1 by siRNA, both ACLY activity and the amount of acetyl-CoA decreased compared to control siRNA-transfected cells (Figure 2A). Similar results were observed in 53BP1 knockout mice embryonic fibroblasts (53BP1^−/−^ MEF) (Figure 2A). The decrease of both ACLY activity and acetyl-CoA levels in 53BP1-deficient cells was comparable to that of ACLY-knocked down cells (Figure 2B). Full-length 53BP1 or 53BP1-N could specifically increase the activity of overexpressed ACLY up to 2-fold (Figure 2C and Supplementary Figure S2A), suggesting the importance of 53BP1-binding to ACLY for its activity.

To examine the regulatory mechanism of ACLY activity by this interaction, we looked more closely at the CoA ligase domain of ACLY which contains a phosphorylation site at histidine 760 (H760) (32,41,42). H760 of ACLY is phosphorylated by NDPK and the H760A mutant was shown to impair the ACLY enzymatic activity (29). Consistent with this report, histidine (His) phosphorylation in ACLY was significantly inhibited by the knockdown of NDPK (Supplementary Figure S2B). To investigate whether the interaction between 53BP1 and ACLY is important for the phosphorylation of ACLY H760, we first tested whether the anti-phospho-His antibody used for Supplementary Figure S2B is specific to the phosphorylation of ACLY H760. To this end, GFP-ACLY WT and a phospho mutant H760A were tested for their phosphorylation by NDPK using the anti-His antibody. As shown in Figure 2D, GFP-ACLY WT but not the H760A mutant was specifically phosphorylated by NDPK (lanes 2–4 from left). Additionally, overexpression of NDPK could not promote the phosphorylation of ACLY-H760A mutant (Figure 2D, lanes 4 and 5 from left), demonstrating that anti-phospho-His antibody-detectable phosphorylation of ACLY H760 is mediated by NDPK. Next, we asked whether the phosphorylation of ACLY H760 is dependent on the presence of 53BP1. Immunoprecipitations of endogenous ACLY followed by western blot analysis showed a reduction of H760 phosphorylation in ACLY in both 53BP1^−/−^ MEFs and 53BP1-depleted HeLa cells (Figure 2E). Consistent with our interaction mapping results, overexpression of 53BP1-N, but not 53BP1-C, also increased the phosphorylation of overexpressed GFP-ACLY H760 (Figure 2F). Because NDPK-mediated phosphorylation of ACLY H760 is promoted by the presence of 53BP1 or ACLY-binding 53BP1 N-terminal fragment, we next investigated whether the interaction between ACLY and NDPK is promoted in the presence of 53BP1. Co-immunoprecipitation experiments were conducted using anti-ACLY or anti-NDPK antibodies in the presence or absence of 53BP1 knockdown in HeLa cells. As shown in Figure 2G, the interaction between NDPK and ACLY was significantly impaired by the knockdown of 53BP1. These results suggest that 53BP1 promotes the interaction between ACLY and NDPK and activates ACLY through NDPK-mediated phosphorylation of H760.

### 53BP1 affects global histone acetylation.

**Figure 3.**
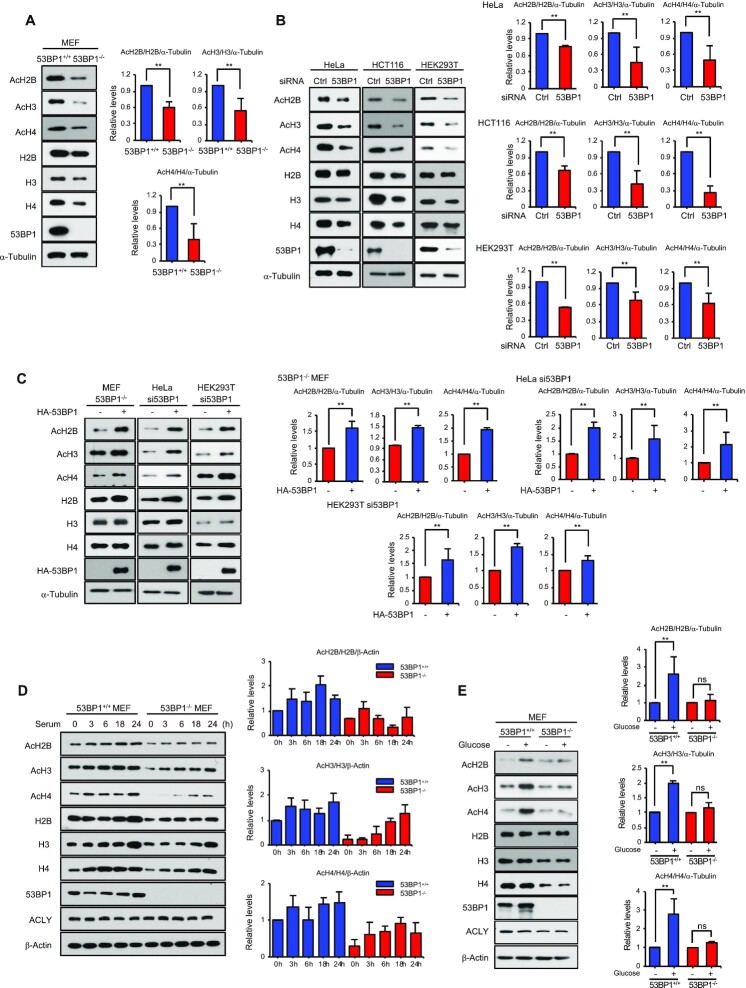
53BP1 regulates global histone acetylation. **(A)** Total cell extracts from 53BP1^+/+^ and 53BP1^−/−^ MEFs were analyzed for total and acetylated histones by western blotting using the indicated antibodies. Quantitation of acetylated histones was done by normalizing to total histone/α-tubulin (please refer to materials and methods for details of the procedure). **(B)** Western blot analyses of total and acetylated histones in HeLa, HCT116, and HEK293T cells with control or 53BP1 knockdown. Quantitation of acetylated histone was done by normalizing to total histone/α-tubulin. The results are shown as the mean ± SD (n = 3), ^∗∗^*P* < 0.01, Student's *t*-test. **(C)** HA-53BP1 was reconstituted in 53BP1^−/−^ MEFs, 53BP1-depleted HeLa and HEK293T cells by overexpression for 48 h. Histone acetylation and total amounts of histones were analyzed using the indicated antibodies. Quantitation of acetylated histone was done by normalizing to total histone/α-tubulin. The results are shown as the mean ± SD (n = 3), ^∗∗^*P* < 0.01, Student's *t*-test. **(D)** 53BP1^+/+^ and 53BP1^−/−^ MEFs were starved by serum depletion for 16 h and subsequently stimulated with 10% fetal bovine serum-containing media for the indicated time points. Total cell lysates were analyzed for total and acetylated histones by western blotting using the indicated antibodies. Quantitation of acetylated histone was done by normalizing to total histone/α-tubulin. The results are shown as the mean ± SD (n = 3). **(E)** 53BP1^+/+^ and 53BP1^−/−^ MEFs were cultured for 48 h in the presence or absence of 25 mM glucose. Total cell lysates were analyzed by western blotting using the indicated antibodies. Quantitation of acetylated histone was done by normalizing to total histone/α-tubulin. The results are shown as the mean ± SD (n = 3), ^∗∗^*P* < 0.01. ns, not significant, Student's *t*-test.

ACLY is a well-characterized cytoplasmic enzyme but a nuclear function of ACLY in histone acetylation was reported (28). Thus, we hypothesized that 53BP1 modulates this nuclear function of ACLY. To test this idea, we measured acetylated histone levels in the presence or absence of 53BP1 using western blots and found that acetylation of histone proteins decreased in cells lacking 53BP1 (Figure 3A and B, and Supplementary Figure S3A and B). Quantitation of western blots by two independent normalization methods (a double normalization to a housekeeping gene product α-tubulin and histone proteins or a normalization to equal loaded histone proteins) showed a significant decrease of histone acetylation upon the 53BP1 deficiency (Figure 3A and B, and Supplementary Figure S3A-D). Notably, we also observed that the overall level of histone proteins was consistently lower in 53BP1-deficient cells than 53BP1-positive cells (Figure 3A and B and Supplementary Figure S3A and B; see below for details). Reconstitution of 53BP1 in these 53BP1-deficient cells was sufficient to restore both acetylated histone and total histone levels (Figure 3C and Supplementary Figure S3E and F).

The dynamic changes in histone acetylation that occur in response to mitogenic stimulation are mediated by ACLY (28). To test whether 53BP1 contributes to this process, we conducted serum starvation and add-back experiments using control and 53BP1^−^/^−^ MEFs and both acetylated and total histone protein levels were measured using western blotting. As reported previously (28), histone acetylation in control cells increased as cells progressed into mitogenesis (Figure 3D and Supplementary Figure S3G and H). Although ACLY levels were similar in both cell lines, there was a significant decrease in histone acetylation across the tested time points in 53BP1^−^/^−^ MEFs. As glucose is a substrate for the ACLY-mediated synthesis of acetyl-CoA (27) and critical for histone acetylation, we also examined whether 53BP1 is involved in glucose-induced histone acetylation. While histone acetylation was highly dependent upon the supply of glucose in control MEFs, glucose-induced histone acetylation in 53BP1^−^/^−^ MEFs was markedly decreased despite the presence of a comparable amount of ACLY (Figure 3E and Supplementary Figure S3I and J). These results suggest that 53BP1 is required for ACLY-mediated histone acetylation.

### 53BP1 is important for cellular production of histone proteins.

**Figure 4.**
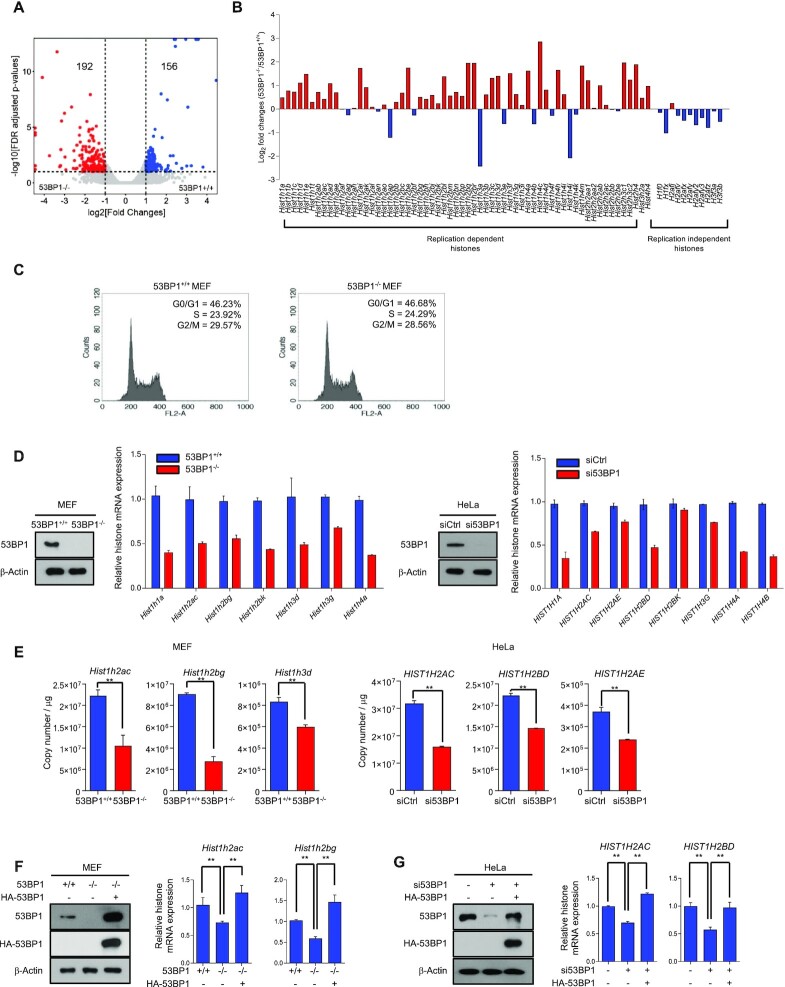
53BP1 is important for the histone gene expression. **(A)** Differential gene expression analysis of 53BP1^+/+^ and 53BP1^−/−^ MEFs using RNA-seq. The X-axis in the volcano plot indicates fold changes of gene expression in 53BP1^+/+^ and 53BP1^−/−^ MEFs and the Y-axis indicates log value of FDR-adjusted p-values. FDR cutoff 0.1 and fold change cutoff 2 were used to highlight differential gene expression in the analysis. **(B)** Fold-changes (log_2_) of the polyadenylated replication-dependent and replication-independent histone gene families in the RNA-seq data from 53BP1^+/+^ and 53BP1^−/−^ MEFs. **(C)** Cell cycle distribution in 53BP1^+/+^ and 53BP1^−/−^ MEFs. G0/G1, S, and G2/M indicate cell cycle phases. **(D)** Total RNAs from 53BP1^+/+^ and 53BP1^−/−^ MEFs (left) or control siRNA- and 53BP1 siRNA-transfected HeLa cells (right) were analyzed for the expression of histone mRNAs by RT-qPCR using random-priming. Western blots show the level of 53BP1 in cells used in these experiments. The results are shown as the mean ± SD (n = 3). **(E)** Absolute quantitation of histone transcripts from select histone genes was conducted using 53BP1^+/+^, 53BP1^−/−^ MEFs and 53BP1 depleted-HeLa cells by RT-qPCR. The results are shown as the mean ± SD (n = 3), ^∗∗^*P* < 0.01, Student's *t*-test. **(F and G)** RT-qPCR analyses of *Hist1h2ac* and *Hist1h2bg* transcripts in 53BP1^+/+^, 53BP1^−/−^, and 53BP1-reconstituted 53BP1^−/−^ MEFs (F). The same analyses for *HIST1H2AC* and *HIST1H2BD* transcripts were performed in control, 53BP1 depleted-HeLa cells with or without reconstitution of 53BP1 (G). The results are shown as the mean ± SD (n = 3), ^∗∗^*P* < 0.01. ns, not significant, Student's *t*-test.

Because 53BP1 affects overall histone acetylation, it is likely to play a role in epigenetic programming and alterations of the transcriptome (43). To profile 53BP1-mediated changes in gene expression, we performed RNA sequencing (RNA-seq) experiments using poly(A+) RNAs purified from 53BP1^−/−^ and 53BP1^+/+^ MEFs. Differential gene expression analysis of our RNA-seq data showed that 53BP1^−/−^ MEFs enriched cellular processes associated with response to *Staphylococcus aureu*s infection and osteoclast differentiation, and downregulated cellular pathways such as DNA replication, cell cycle, and pyrimidine metabolism (Supplementary Figure S4A and B). Interestingly, we also found that numerous replication-dependent histone genes were upregulated in 53BP1^−/−^ MEFs (Systemic lupus erythematosus and alcoholism in KEGG pathways; Figure 4A-C, Supplementary Figure S4C, and Supplementary Tables S1 and S2). To confirm these results, we performed real-time quantitative PCR (RT-qPCR) to measure the relative expression of histone genes using cDNAs synthesized by random primers as replication-dependent histone mRNAs do not contain poly(A) tails (Supplementary Figure S4D) (44). Surprisingly, opposite to our RNA-Seq results, RT-qPCR analyses showed an overall downregulation of numerous histone genes upon the knockout of 53BP1 (Figure 4D). We reasoned that these opposite results on histone gene expression using two different experimental approaches might come from the polyadenylation of histone mRNAs. Generally, replication-dependent histone mRNAs undergo distinct processing and do not receive polyadenylation that most eukaryotic mRNAs do (45). For this reason, histone mRNAs minimally show up in RNA-seq experiments using poly(A)+ RNAs. Thus, our transcriptome profiling suggests that the 53BP1-knockout transcriptome contains a higher amount of polyadenylated histone mRNAs compared to that of 53BP1^+/+^ MEFs. To test this idea, we measured the relative amount of polyadenylated histone mRNAs using (oligo(dT)-primed cDNA) (Supplementary Figure S4E). In this case, consistent with our RNA-Seq results, polyadenylated histone mRNAs were increased in 53BP1^−/−^ MEFs (Supplementary Figure S4E). A collection of these observations was recurrent in 53BP1-depleted HeLa cells (Figure 4D and Supplementary Figure S4E). Further absolute quantitation of select total and polyadenylated histone mRNAs using RT-qPCR confirmed these results and also showed that while the increase of polyadenylated histone mRNAs was substantial upon 53BP1 deficiency, their fractions compared to total histone mRNA levels were varied by histone genes and they did not contribute to the total amounts of histone mRNAs in cells (Figure 4E and Supplementary Figure S4F). Additionally, these results are consistent with our western blot analyses (Figure 3A and B) and demonstrate that the 53BP1 deficiency in cells leads to the impairment of histone mRNA and protein production. Reconstitution of 53BP1 in 53BP1-deficient cells was sufficient to rescue the level of total histone mRNAs (Figure 4F and G) while decreasing the level of polyadenylated histone mRNAs (Supplementary Figure S4G).

### 53BP1 controls the expression of SLBP, a master regulatory of histone biogenesis.

**Figure 5.**
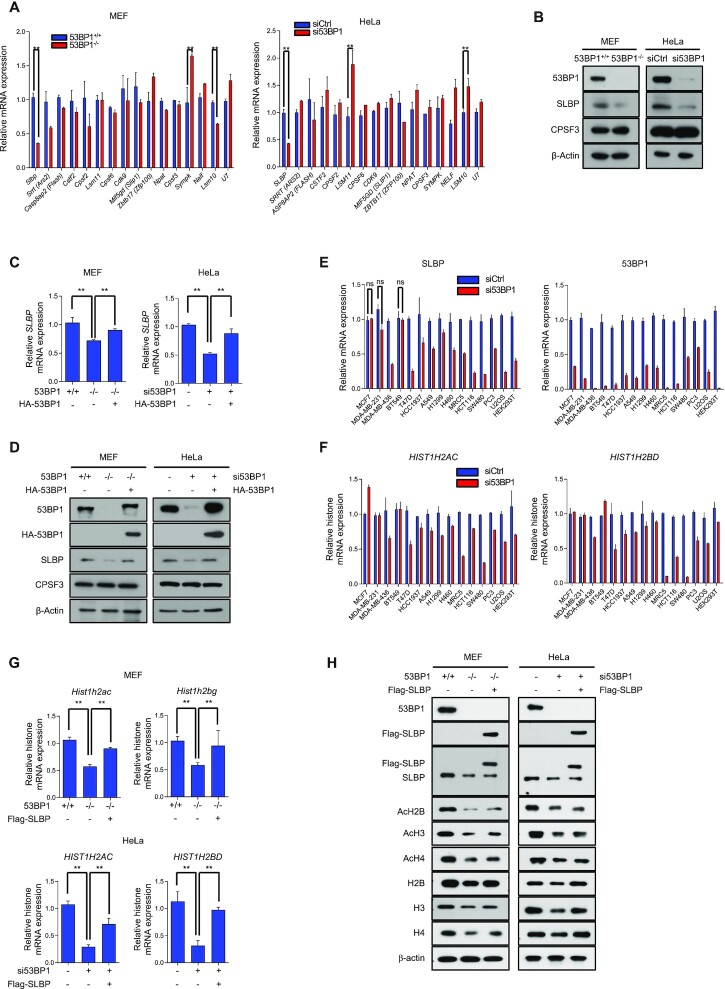
53BP1 regulates the expression of SLBP and affects histone biogenesis. **(A)** RT-qPCR analysis of representative genes known for their function in histone mRNA biogenesis. Total RNAs from 53BP1^+/+^ and 53BP1^−/−^ MEFs (left) or control and 53BP1 siRNA-transfected HeLa cells (right) were used. The results are shown as the mean ± SD (n = 3), ^∗∗^*P* < 0.01, Student's *t*-test. **(B)** Western blot analyses of SLBP expression using 53BP1^+/+^ and 53BP1^−/−^ MEFs or control siRNA- and 53BP1 siRNA-transfected HeLa cells. CPSF3 is a known processing factor for histone gene expression and was used as a control. (**C**) Relative measurement of SLBP mRNA expression was done by RT-qPCR in 53BP1^+/+^, 53BP1^−/−^, and 53BP1-reconstituted 53BP1^−/−^ MEFs or control, 53BP1-depleted HeLa cells, and 53BP1-depleted HeLa cells with 53BP1 reconstitution. The results are shown as the mean ± SD (n = 3), ^∗∗^*P* < 0.01, Student's *t*-test. **(D)** 53BP1 was reconstituted in 53BP1^−/−^ MEFs or 53BP1-depleted HeLa cells and the changes of SLBP expression were analyzed by western blotting. **(E and F)** 53BP1 was knocked down in the indicated human immortalized or cancer cell lines by siRNA. 48 h after transfection, the levels of SLBP and 53BP1 mRNA (E), and *HIST1H2AC* and *HIST1H2BD* mRNA (F) were analyzed by RT-qPCR. The results are shown as the mean ± SD (n = 3). ns, not significant, Student's *t*-test. **(G)** RT-qPCR analysis of *HIST1H2AC* and *HIST1H2BG/D* mRNAs in 53BP1^+/+^, 53BP1^−/−^, and SLBP-reconstituted 53BP1^−/−^ MEFs (left), or control, 53BP1 depleted-HeLa cells, and SLBP-reconstituted 53BP1 knockdown HeLa cells (right). The results are shown as the mean ± SD (n = 3), ^∗∗^*P* < 0.01, Student's *t*-test. **(H)** Analysis of total and acetylated histones H2B, H3 and H4 by western blotting in SLBP-reconstituted 53BP1^−/−^ MEFs or 53BP1-depleted HeLa cells with SLBP reconstitution.

Previous studies have shown that multiple 3′-end processing factors play a key role for the expression of histone genes and their downregulation leads to the decrease of histone mRNAs and proteins (45–48). These studies also noted that the decrease of histone gene expression accompanies the increase of polyadenylated histone mRNAs. These observations are along the same line as the phenotypic changes of histone gene expression in 53BP1-deficient cells. Therefore, we first profiled the differential gene expression of these factors in our RNA-seq data (Supplementary Table S3) and then asked whether the expression of these factors was indeed changed in 53BP1-deficient cells using RT-qPCR (Figure 5A). Among those known factors for histone mRNA processing, the expression of stem-loop binding protein (SLBP) moderately decreased in our RNA-Seq data analysis (Supplementary Table S3). SLBP is a master regulator of histone biogenesis from transcription-coupled 3′-end processing of histone mRNAs in the nucleus to translation in the cytoplasm (45,49). Consistent with our RNA-Seq data, qPCR analysis showed that SLBP was downregulated in both 53BP1^−/−^ MEFs and 53BP1-depleted HeLa cells (Figure 5A and B). Importantly, the reconstitution of 53BP1 was sufficient to rescue this downregulation of SLBP in 53BP1-deficient cells at both mRNA and protein levels (Figure 5C and D). Similar phenotypic changes of SLBP downregulation were observed upon the knockdown of 53BP1 in many human cell lines with the exception of three breast cancer cell lines (Figure 5E). Consistently, this 53BP1-dependent SLBP expression has a positive correlation with cellular level of total histone transcripts (Figure 5F). Moreover, the reconstitution of SLBP in 53BP1-deficient cells was sufficient to rescue the total level of histone transcripts and proteins and the rescue minimally affected the profile of cell cycle (Figure 5G and Supplementary Figure S5A-C). However, the SLBP reconstitution did not rescue the acetylation of histone proteins (Figure 5H). These data suggest that the phenotypic changes of histone gene expression seen in 53BP1-deficient cells are coming from the downregulation of *SLBP* expression, and this phenotype appears to be conserved in most normal and human cancer cells.

Because 53BP1 is a transcriptional coactivator with p53 (50), it is plausible that the 53BP1-dependent expression of *SLBP* is also mediated by p53. However, we observed similar levels of SLBP expression in both HCT116 p53^+^/^+^ and HCT116 p53^−^/^−^ cells, arguing against a potential role of p53 in this process (Supplementary Figure S6A and B). Furthermore, the knockdown of 53BP1 in both cell lines led to the decrease of SLBP expression, indicating that 53BP1-dependent SLBP and histone gene expression occurs regardless of the presence of p53 in cells (Supplementary Figure S6C-E). All of these results demonstrate that the regulation of SLBP expression by 53BP1 is independent of p53 functions.

**Figure 6.**
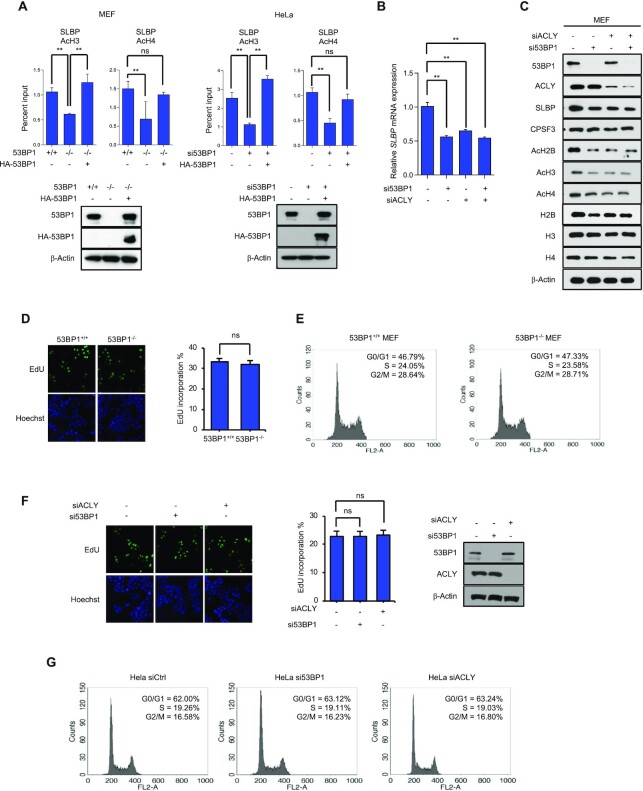
53BP1 positively regulates the SLBP expression by altering the acetylation status of the *SLBP* promoter. **(A)** Chromatin immunoprecipitation (ChIP)-qPCR analysis of the *SLBP* and *CPSF3* promoters in 53BP1^+/+^ and 53BP1^−/−^ MEFs (left) or control and 53BP1 siRNA-transfected HeLa cells (right). ChIP-qPCR was performed using antibodies to acetylated histone H3 (Ac-H3) and H4 (Ac-H4). Data represent ChIP enrichment relative to input. The results are shown as the mean ± SD (n = 3), ^∗∗^*P* < 0.01, Student's *t*-test. (**B and C**) The levels of SLBP mRNA (B) and protein (C) in MEF cells transfected with control siRNA-, 53BP1 siRNA-, or ACLY siRNA in the indicated combinations. The results are shown as the mean ± SD (n = 3), ^∗∗^*P* < 0.01, Student's *t*-test. (**D**) 53BP1^+/+^ and 53BP1^−/−^ MEFs were stained with propidium iodide and the DNA content was analyzed by flow cytometry. G0/G1, S, and G2/M indicate cell cycle phases. (**E**) The number of 53BP1^+/+^ and 53BP1^−/−^ MEFs in the S-phase was detected by EdU incorporation assay. EdU staining (red) for S-phase cells. Hoechst staining (blue) for the cell nuclei. Lower panel shows quantification of the percentage of EdU-positive cells. The results are shown as the mean ± SD (n = 3). ns, not significant, Student's *t*-test. (**F**) Cell cycle profiles of control, 53BP1-, and ACLY-depleted HeLa cells obtained by flow cytometry. (**G**) The S-phase cells of control, 53BP1-, and ACLY-depleted HeLa cells were stained by EdU incorporation. Lower panel shows the percentage of EdU-positive cells. Data are presented as mean ± SD (n = 3). Student's t-test was used. ns, not significant, Student's *t*-test.

To gain mechanistic insight into 53BP1-mediated *SLBP* expression, we asked whether epigenetic regulation of gene expression, particularly histone acetylation (51,52) (50,51) (50,51), plays a role. Using a chromatin immunoprecipitation (ChIP)-qPCR assay and anti-acetyl H3 and H4 antibodies, we observed that levels of acetylated H3 and H4 at the *SLBP* promoter were highly dependent on 53BP1 and they were restored to normal levels when 53BP1 was reconstituted in 53BP1-deficient cells (Figure 6A and B). We also checked the *CPSF3* promoter as a control and observed that 53BP1 knockdown did not affect the level of acetylated H3 at the *CPSF3* promoter (Supplementary Figure S7A).

To ask whether the impairment of ACLY activity upon 53BP1 deficiency is a major reason for the downregulation of SLBP and consequently a defect in histone biogenesis, we knocked down ACLY and examined the SLBP expression. In the ACLY-knockdown HeLa cells, the *SLBP* expression decreased at both mRNA and protein levels, and the steady state levels of both histone acetylation and histone protein also decreased (Fig. 6C, and Supplementary Figure S7B). Consistently, the knockdown of ACLY also phenocopied the 53BP1-dependent SLBP expression and histone gene expression in most human cell lines we tested (Supplementary Figure S7C and D). Although the knockdown of either 53BP1 or ACLY phenocopied each other in histone biogenesis, the knockdown of one or the other (53BP1 or ACLY) did not affect the protein level of the other one (Figure 6C). Impairments of histone biogenesis, SLBP expression, and acetylation in the *SLBP* promoter caused by the 53BP1 knockdown were restored by the introduction of 53BP1-N, but not 53BP1-H3 nor 53BP1-H6 (Supplementary Figure S7E-H), again validating that the interaction between 53BP1 and ACLY is important for the expression of SLBP. To further test whether phosphorylation of H760 contributes to SLBP expression, cells expressing GFP-ACLY-H760A were treated with siRNAs targeting ACLY 3$\prime$UTR to deplete endogenous ACLY. Then SLBP expression and the acetylation of the SLBP promoter were examined. We found that GFP-ACLY-H760A was not able to restore the SLBP expression level nor H3 acetylation at the SLBP promoter (Supplementary Figure S8A-C). Next, we looked at whether NDPK contributes to 53BP1-mediated SLBP expression. To this end, NDPK was knocked down using siRNA in 53BP1-deficient MEF and HeLa cells, and subsequently 53BP1 was reconstituted in these cells. As shown in Supplementary Figure S8D and E, reconstituted 53BP1 in NDPK-knocked down cells was not able to rescue the SLBP expression and H3 acetylation at the SLBP promoter.

Our RNA-Seq data shows that 53BP1 knockout downregulates genes associated with DNA replication and cell cycle (Supplementary Figure S4A). To test whether cell cycle was affected due to 53BP1 deficiency, we examined the changes of cell cycle upon the knockout or knockdown of 53BP1 using MEFs or HeLa cells respectively. We also measured the effect of ACLY knockdown in HeLa cells as an additional control. As shown in Figure 6D-G, there were no detectable perturbations of cell cycle in asynchronous cells deficient of 53BP1 or ACLY expression. Consistent with these observations, reconstitution of 53BP1 in 53BP1-deficient MEFs and HeLa cells had no observable effects on cell cycle (Supplementary Figure S8F). However, when the progression of cell cycle was traced by time course after the release of double thymidine blocked cells, we found that 53BP1-deficient cells display a slower progression of S phase compared to 53BP1-positive cells (Supplementary Figure S8G). Since the expression of SLBP and replication dependent histone genes are known to associate with cell cycle (44,45,53,54), we examined whether the phenotypic changes of histone mRNA processing are due to the decrease of gene expression associated with cell cycle or DNA replication. To this end, we knocked down exemplary three genes (*Cdc7*, *Ccna2*, and *Mcm5*) that are downregulated in 53BP1 knockout MEFs using WT MEFs and measured the level of total and polyadenylated histone mRNAs. We found that the level of total and polyadenylated histone mRNAs and SLBP transcript were not significantly changed upon the knockdown of *Cdc7*, *Ccna2*, or *Mcm5* (Supplementary Figure S9A-C). These results suggest that the phenotypic changes in SLBP expression and histone biogenesis in 53BP1-deficient cells are not likely coming from the changes of cell cycle. Taken all together, these findings provide evidence that 53BP1-coordinated phosphorylation of ACLY by NDPK is a major regulatory pathway for SLBP expression and histone biogenesis.

### 53BP1-coordinated SLBP expression is crucial for S-phase progression, recovery of stalled replication forks, and genomic integrity.

**Figure 7.**
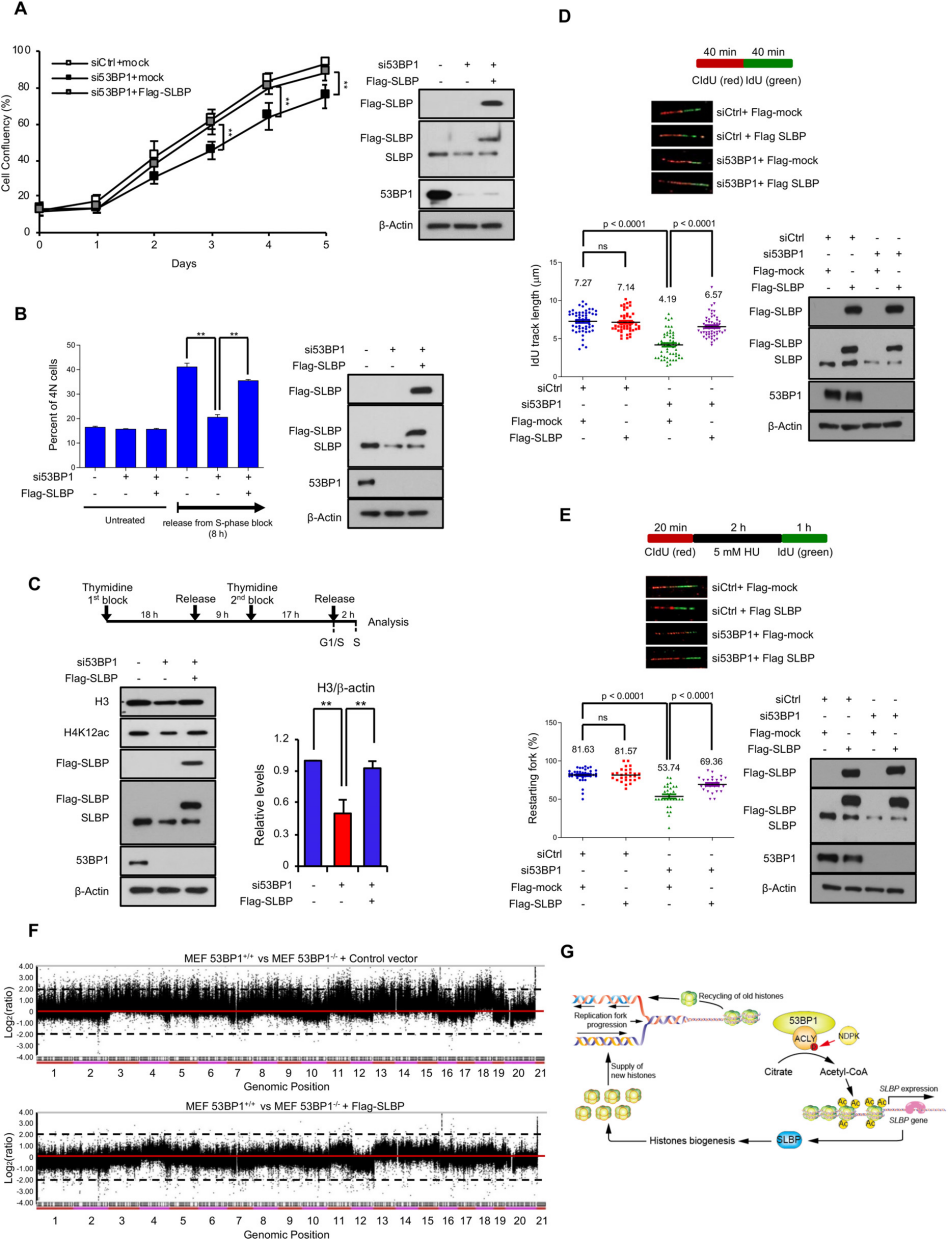
53BP1-ACLY-SLBP regulatory axis contributes to S-phase progression and genomic integrity. **(A)** Proliferation of control (siCtrl), 53BP1-depleted (si53BP1) HeLa cells, and 53BP1-depleted HeLa cells with SLBP overexpression was monitored by live cell imaging for 5 days. The results are shown as the mean ± SD (n = 3), ^∗∗^*P* < 0.01, Student's *t*-test. **(B)** The indicated siRNA and/or vector-transfected HeLa cells were treated with aphidicolin to arrest cells in S-phase. Then cells were incubated with colcemid-containing medium to release from the arrest and to trap in M-phase. Cell cycle profiles were measured by flow cytometry. The results are shown as the mean ± SD (n = 3), ^∗∗^*P* < 0.01, Student's *t*-test. **(C)** The experimental design for S-phase synchronization (top) is shown. The indicated siRNA and/or vector-transfected HeLa cells were synchronized at S-phase through a two-step thymidine block. Total cell extracts were prepared and analyzed by western blotting using the indicated antibodies (bottom). **(D)** Replication elongation rates of control, 53BP1-depleted, and Flag-SLBP-reconstituted/53BP1-depleted HeLa cells were measured. Each cell was treated with CIdU (red) and IdU (green) for 40 min each and the IdU track length of CIdU-positive fibers was measured. Data represent mean ± SD (n = 3). *P* values between indicated samples were calculated using a Mann-Whitney test. **(E)** Replication fork restart of control, 53BP1-depleted, SLBP-reconstituted 53BP1-depleted HeLa cells was measured. Pulse-labelling of cells with CIdU, HU (hydroxyurea), and IdU was done as shown in the schematic of experiment. DNA fibers were counted by contiguous IdU and CIdU tracks. Data represent mean ± SD (n = 3). *P* values between indicated samples were calculated using a Mann-Whitney test. **(F)** Array CGH profiles of clones derived from 53BP1^−/−^ MEFs (top), 53BP1^−/−^ MEF with SLBP-reconstitution (bottom) are shown. Chromosomal regions above or below the dotted line indicate amplifications or deletions of genomic regions, respectively. (**G**) A model of the newly characterized role for 53BP1 in quantitative and qualitative histone homeostasis. The interaction between 53BP1 and ACLY enhances histidine phosphorylation of ACLY by NDPK, leading to increased expression of SLBP, which then influences histone mRNA processing and production of new histones. This pathway is critical for S-phase progression and chromosomal stability.

SLBP is essential for histone biogenesis in S-phase, and the downregulation of SLBP leads to the inhibition of DNA replication, cell growth, and prolonged S-phase (45,49,53,55). To determine whether 53BP1 is an upstream regulator of these processes, we knocked down either SLBP or 53BP1 in HeLa cells and measured cell proliferation and cell cycle progression. Although asynchronous 53BP1-deficient cells did not show changes in cell cycle compared to 53BP1-positive cells (Figure 4C), the knockdown of either 53BP1 (Figure 7A) or SLBP (Supplementary Figure S10A) impaired cell proliferation. In addition, when the cells with either knockdown were synchronized and released using aphidicolin and colcemid respectively, the accumulation of 4N cells increased significantly compared to untreated cells, indicating a delayed progression from S to M phase (Figure 7B and Supplementary Figure S10B and C). Overexpression of SLBP in 53BP1-depleted HeLa cells was sufficient to restore normal cell proliferation and S-phase progression (Figure 7A and B). Because SLBP is also important for the supply of newly synthesized histones during S-phase (45,49,53,55), we measured both newly synthesized histone H4 marked by K12 acetylation (H4K12ac) and the soluble pool of histone H3 (56) by western blotting in cells synchronized at S-phase with a thymidine block. Consistent with previous findings (55), both readouts of newly synthesized histone proteins were reduced in either the SLBP knockdown or the 53BP1 knockdown and the reconstitution of SLBP was sufficient to restore the newly synthesized histone proteins (Figure 7C and Supplementary Figure S10D and E). Likewise, knockdown of either 53BP1 or SLBP reduced the rate of replication fork progression, as measured by the single DNA fiber assay, and the replication fork progression was restored by the reconstitution of SLBP (Figure 7D and Supplementary Figure S10F). Both DNA synthesis and histone deposition are required to restart replication folks after replication fork stalling (57–59). Consistent with our findings in the role of 53BP1 in histone biogenesis, a defect of replication fork progression was observed in both 53BP1- and SLBP-depleted cells after the treatment of hydroxyurea (HU) (Figure 7E and Supplementary Figure S11G). This defective phenotype could be also rescued by the reconstitution of SLBP in 53BP1-depleted cells (Figure 7E).

Because a regulatory phosphorylation of nuclear ACLY at S455 occurs by ATM following DNA damage and the function of 53BP1 coordinated by DNA damage (6,40,60), we investigated whether 53BP1-mediated H760 phosphorylation in ACLY and histone biogenesis are also affected by DNA damage. We first tested whether DNA damage-inducing IR or hydroxyurea (HU) treatment on cells, or cell cycle affects the phosphorylation of ACLY H760. We found that ACLY H760 phosphorylation was not significantly changed by DNA damage or cell cycle (Supplementary Figure S11A-C). Moreover, all alanine substitution mutant of ATM-targeted 20 phosphorylation sites in the S/TQ motifs (53BP1-20AQ) showed a similar level of binding to ACLY and maintained its capacity to increase the phosphorylation of ACLY H760 (Supplementary Figure S11D). In addition, phospho-mutation of ACLY at S455 or H760 did not affect each other's phosphorylation status (Supplementary Figure S11E), suggesting that the regulatory phosphorylation of these two sites in ACLY is independent. Consistent with these observations, IR exposure did not affect the levels of total and acetylated histones (Supplementary Figure S11F), the expression of total and polyadenylated histone mRNAs (Supplementary Figure S11G), and SLBP expression (Supplementary Figure S11H). Of note, 53BP1 or ACLY knockdown-mediated reduction of histone biogenesis and SLBP expression was not affected by IR treatment (Supplementary Figure S11F-H). Together, these results indicate that the phenotypic changes of histone biogenesis found in 53BP1-depleted cells are independent of DSB repair pathway. This idea is further supported by the following observations: the reconstitution of ACLY-WT but not ACLY-H760A ameliorated the impairment of S-phase progression and delayed re-activation of stalled replication forks in ACLY-deficient cells (Supplementary Fig. S12A-C) and the reconstitution of 53BP1-20AQ in 53BP1-deficient cells rescues S-phase progression, replication forks progression, and recovery of stalled replication forks (Supplementary Fig. S12D-F).

Because defects in histone biogenesis during S-phase are detrimental to genomic integrity (55,61,62), we investigated whether 53BP1-regulated expression of SLBP is important for the maintenance of genomic integrity using array comparative genomic hybridization (array CGH) and found an increased frequency of chromosomal rearrangements, including clonal amplifications and deletions, was observed in the 53BP1 knockdown cells as well as the SLBP knockdown cells (Figure 7F and Supplementary Figure S13A-C). Remarkably, this high frequency of chromosomal abnormalities observed in 53BP1^−^/^−^ MEFs was effectively reduced by the reconstitution of SLBP (Figure 7F). These results provide evidence that the genomic instability caused by the deficiency of 53BP1 comes from the downregulation of SLBP and further establish a regulatory axis of 53BP1-ACLY-SLBP in histone biogenesis and genome integrity.

## DISCUSSION

In this study, we describe a DSB repair-independent function of 53BP1. We found ACLY as a new interactor of 53BP1 and that their interaction is crucial for the phosphorylation of ACLY H760 by NDPK and consequently, an increase in ACLY activity. Although it needs further investigations to understand how the binding of 53BP1 to ACLY facilitates the phosphorylation of ACLY by NDPK, this newly discovered regulatory mechanism suggests that 53BP1 serves as a molecular scaffold to control the ACLY function. Numerous studies have been focusing on the cytosolic function of ACLY in the production of acetyl-CoA from citrate (39). A nuclear function of ACLY in HR has begun to be understood recently (40). Acetyl-CoA is a major donor of the acetyl group in protein modifications and thus, ACLY is proposed to function in the nuclear protein acetylation such as histone acetylation (28). Together with our findings, these place 53BP1 as a potential upstream modulator of ACLY-driven histone acetylation and epigenetic program. As our RNA-seq experiments showed, hundreds of genes were either upregulated or downregulated in the absence of 53BP1, and these transcriptome changes were associated with many cellular pathways. Our findings highlight a previously uncharacterized function of 53BP1 in the regulation of gene expression and further suggest that 53BP1 affects diverse biological processes.

Strikingly, one of the cellular pathways affected by the 53BP1-ACLY-NDPK regulatory axis in our study is the regulation of cellular histone biogenesis (Figure 7G). Specifically, the deficiency in 53BP1 or ACLY causes downregulation of SLBP, a master regulator of histone mRNA metabolism in both the nucleus and cytoplasm, and renders genomic instability by disrupting histone biogenesis. Consistent with these findings, our custom analysis of published data showed that a pharmacological inhibition of ACLY in prostate cancer cells (63) phenocopied both SLBP downregulation and histone mRNA polyadenylation, a potential indicator for the decrease of histone transcripts as observed in 53BP1-deficient cells in this study (Supplementary Fig. S14 and Supplementary Table S4). Moreover, the data also illustrate that the inhibition of ACLY activity leads to drastic changes of global gene expression, indicating the involvement of ACLY in the transcriptome programming. The fact that 53BP1 and ACLY have a combined regulatory function opens up the possibility of identifying unexplored roles for these proteins in other diverse cellular processes. For example, because 53BP1 controls the ACLY activity, it may then be an upstream modulator of acetyl-CoA, a central metabolite that controls cellular processes such as energy metabolism, mitosis, autophagy, mitophagy, and aging (27,39,40,64,65). ACLY is a well-characterized metabolic enzyme in the cytoplasm but its function in the nucleus, where it affects histone acetylation and DSB repair, are becoming clearer (40). Collectively, the 53BP1-ACLY regulatory axis could play a role in the changes of genome-wide histone acetylation and specifically modulates histone biogenesis and genome integrity through the epigenetic regulation of SLBP expression. We further argue that our conclusions provide a new regulatory crosstalk mechanism that connects the nutritional environment of cells to genome integrity as ACLY supports the cellular levels of acetyl-CoA.

Histone biogenesis and deposition during S-phase is crucial for DNA replication, progression of S-phase, and resolution of stalled replication forks (57–59,66). Extensive characterization of histone biogenesis in the context of both trans- and cis-acting elements has been made (45–47,49,67,68). For example, downregulation of SLBP, FLASH, or SRRT showed a decrease in histone biogenesis. However, upstream regulatory pathways of these proteins are yet elusive. Our findings provide mechanistic insight into how histone biogenesis is coordinated at multiple levels by 53BP1. We found that the reconstitution of SLBP alleviated the genomic instability caused by the 53BP1 deficiency. The reconstitution of SLBP brought back histone levels to normal. However, there is no evidence for a role of SLBP in DSB repair as SLBP is known to play a critical role in the processing, nuclear export, translation and degradation of histone mRNA (45). Therefore, the chromosomal abnormalities that occur in 53BP1-depleted cells are likely due to the lack of histone supply in S-phase. Although we cannot completely rule out the possibility of involving other cellular pathways that are affected by 53BP1 deficiency in cells, our conclusions provide an explanation to previous observations that were not clearly answered using a traditional model of 53BP1-mediated DSB repair: how 53BP1 contributes to the recovery of stalled replication forks (23–25) and how 53BP1 dysregulation leads to genomic instability and the development of cancer (45,61,66,69). Collectively, these results strongly argue that 53BP1 maintains genomic integrity through diverse mechanisms and that genomic instability caused by the 53BP1 deficiency is due in part to impaired histone biogenesis. Our data shed light on the molecular mechanism of 53BP1 that is independent of its role in DSB repair.

Although the mechanistic details and specificity of how ACLY and 53BP1 regulate genome integrity need to be investigated, it is surprising to observe that both ACLY and 53BP1 are involved with DNA damage signaling-dependent and -independent maintenance of genome integrity: the phosphorylation of S455 in ACLY by DNA damage signaling prevents the localization of 53BP1 to DSB sites and promotes BRCA1-driven DNA repair by HR (40), while H760 phosphorylation in ACLY is facilitated by binding to 53BP1 and functions in histone biogenesis. In this regard, various kinases functioning on multiple phosphorylation sites of ACLY could provide crosstalk between diverse cellular mechanisms and/or extracellular environments and genome maintenance. Thus, we envision that the phosphorylation of particular residues in ACLY could serve as a specific molecular switch for ACLY-regulated downstream processes.

The role of 53BP1 in the decision of NHEJ over HR in DSB repair has been extensively discussed (3,4,9,10). However, the long-standing observations that the 53BP1 deficiency increases genomic instability and tumor incidence in animal models (14–17) raise the possibility of multifaceted functions of 53BP1 in the regulation of genomic instability. Our findings provide mechanistic insights of how this multifunctionality of 53BP1 in genome integrity could be achieved by changing the dynamics of its interacting proteins.

## DATA AVAILABILITY

RNA-seq data is available at NCBI with BioProject ID: PRJNA496219.

## Supplementary Material

gkab1300_Supplemental_File

## References

[1] Botuyan M.V. , Lee J., Ward I.M., Kim J.E., Thompson J.R., Chen J., Mer G. Structural basis for the methylation state-specific recognition of histone H4-K20 by 53BP1 and crb2 in DNA repair. Cell. 2006; 127:1361–1373. 17190600 10.1016/j.cell.2006.10.043 PMC1804291

[2] Fradet-Turcotte A. , Canny M.D., Escribano-Diaz C., Orthwein A., Leung C.C., Huang H., Landry M.C., Kitevski-LeBlanc J., Noordermeer S.M., Sicheri F. et al. 53BP1 is a reader of the DNA-damage-induced H2A lys 15 ubiquitin mark. Nature. 2013; 499:50–54. 23760478 10.1038/nature12318 PMC3955401

[3] Chapman J.R. , Barral P., Vannier J.B., Borel V., Steger M., Tomas-Loba A., Sartori A.A., Adams I.R., Batista F.D., Boulton S.J. RIF1 is essential for 53BP1-dependent nonhomologous end joining and suppression of DNA double-strand break resection. Mol. Cell. 2013; 49:858–871. 23333305 10.1016/j.molcel.2013.01.002 PMC3594748

[4] Escribano-Diaz C. , Orthwein A., Fradet-Turcotte A., Xing M., Young J.T., Tkac J., Cook M.A., Rosebrock A.P., Munro M., Canny M.D. et al. A cell cycle-dependent regulatory circuit composed of 53BP1-RIF1 and BRCA1-CtIP controls DNA repair pathway choice. Mol. Cell. 2013; 49:872–883. 23333306 10.1016/j.molcel.2013.01.001

[5] Ochs F. , Somyajit K., Altmeyer M., Rask M.B., Lukas J., Lukas C. 53BP1 fosters fidelity of homology-directed DNA repair. Nat. Struct. Mol. Biol. 2016; 23:714–721. 27348077 10.1038/nsmb.3251

[6] Mirman Z. , Lottersberger F., Takai H., Kibe T., Gong Y., Takai K., Bianchi A., Zimmermann M., Durocher D., de Lange T. 53BP1-RIF1-shieldin counteracts DSB resection through CST- and Polalpha-dependent fill-in. Nature. 2018; 560:112–116. 30022158 10.1038/s41586-018-0324-7 PMC6072559

[7] Ghezraoui H. , Oliveira C., Becker J.R., Bilham K., Moralli D., Anzilotti C., Fischer R., Deobagkar-Lele M., Sanchiz-Calvo M., Fueyo-Marcos E. et al. 53BP1 cooperation with the REV7-shieldin complex underpins DNA structure-specific NHEJ. Nature. 2018; 560:122–127. 30046110 10.1038/s41586-018-0362-1 PMC6989217

[8] Noordermeer S.M. , Adam S., Setiaputra D., Barazas M., Pettitt S.J., Ling A.K., Olivieri M., Alvarez-Quilon A., Moatti N., Zimmermann M. et al. The shieldin complex mediates 53BP1-dependent DNA repair. Nature. 2018; 560:117–121. 30022168 10.1038/s41586-018-0340-7 PMC6141009

[9] Bunting S.F. , Callen E., Wong N., Chen H.T., Polato F., Gunn A., Bothmer A., Feldhahn N., Fernandez-Capetillo O., Cao L. et al. 53BP1 inhibits homologous recombination in Brca1-deficient cells by blocking resection of DNA breaks. Cell. 2010; 141:243–254. 20362325 10.1016/j.cell.2010.03.012 PMC2857570

[10] Bouwman P. , Aly A., Escandell J.M., Pieterse M., Bartkova J., van der Gulden H., Hiddingh S., Thanasoula M., Kulkarni A., Yang Q. et al. 53BP1 loss rescues BRCA1 deficiency and is associated with triple-negative and BRCA-mutated breast cancers. Nat. Struct. Mol. Biol. 2010; 17:688–695. 20453858 10.1038/nsmb.1831 PMC2912507

[11] Jaspers J.E. , Kersbergen A., Boon U., Sol W., van Deemter L., Zander S.A., Drost R., Wientjens E., Ji J., Aly A. et al. Loss of 53BP1 causes PARP inhibitor resistance in Brca1-mutated mouse mammary tumors. Cancer Discov. 2013; 3:68–81. 23103855 10.1158/2159-8290.CD-12-0049 PMC7518105

[12] Gupta R. , Somyajit K., Narita T., Maskey E., Stanlie A., Kremer M., Typas D., Lammers M., Mailand N., Nussenzweig A. et al. DNA repair network analysis reveals shieldin as a key regulator of NHEJ and PARP inhibitor sensitivity. Cell. 2018; 173:972–988. 29656893 10.1016/j.cell.2018.03.050 PMC8108093

[13] Chapman J.R. , Taylor M.R., Boulton S.J. Playing the end game: DNA double-strand break repair pathway choice. Mol. Cell. 2012; 47:497–510. 22920291 10.1016/j.molcel.2012.07.029

[14] Morales J.C. , Franco S., Murphy M.M., Bassing C.H., Mills K.D., Adams M.M., Walsh N.C., Manis J.P., Rassidakis G.Z., Alt F.W. et al. 53BP1 and p53 synergize to suppress genomic instability and lymphomagenesis. Proc. Natl. Acad. Sci. U. S. A. 2006; 103:3310–3315. 16492765 10.1073/pnas.0511259103 PMC1413919

[15] Ward I.M. , Difilippantonio S., Minn K., Mueller M.D., Molina J.R., Yu X., Frisk C.S., Ried T., Nussenzweig A., Chen J. 53BP1 cooperates with p53 and functions as a haploinsufficient tumor suppressor in mice. Mol. Cell. Biol. 2005; 25:10079–10086. 16260621 10.1128/MCB.25.22.10079-10086.2005 PMC1280262

[16] Minter-Dykhouse K. , Ward I., Huen M.S., Chen J., Lou Z. Distinct versus overlapping functions of MDC1 and 53BP1 in DNA damage response and tumorigenesis. J. Cell Biol. 2008; 181:727–735. 18504301 10.1083/jcb.200801083 PMC2396806

[17] Difilippantonio S. , Gapud E., Wong N., Huang C.Y., Mahowald G., Chen H.T., Kruhlak M.J., Callen E., Livak F., Nussenzweig M.C. et al. 53BP1 facilitates long-range DNA end-joining during V(D)J recombination. Nature. 2008; 456:529–533. 18931658 10.1038/nature07476 PMC3596817

[18] Kucharski T.J. , Minshall P.E., Moustafa-Kamal M., Turnell A.S., Teodoro J.G. Reciprocal regulation between 53BP1 and the anaphase-promoting complex/cyclosome is required for genomic stability during mitotic stress. Cell Rep. 2017; 18:1982–1995. 28228263 10.1016/j.celrep.2017.01.080

[19] Fong C.S. , Mazo G., Das T., Goodman J., Kim M., O’Rourke B.P., Izquierdo D., Tsou M.F. 53BP1 and USP28 mediate p53-dependent cell cycle arrest in response to centrosome loss and prolonged mitosis. Elife. 2016; 5:e16270. 27371829 10.7554/eLife.16270 PMC4946878

[20] Lambrus B.G. , Daggubati V., Uetake Y., Scott P.M., Clutario K.M., Sluder G., Holland A.J. A USP28-53BP1-p53-p21 signaling axis arrests growth after centrosome loss or prolonged mitosis. J. Cell Biol. 2016; 214:143–153. 27432896 10.1083/jcb.201604054 PMC4949452

[21] Meitinger F. , Anzola J.V., Kaulich M., Richardson A., Stender J.D., Benner C., Glass C.K., Dowdy S.F., Desai A., Shiau A.K. et al. 53BP1 and USP28 mediate p53 activation and G1 arrest after centrosome loss or extended mitotic duration. J. Cell Biol. 2016; 214:155–166. 27432897 10.1083/jcb.201604081 PMC4949453

[22] Tiwari A. , Addis Jones O., Chan K.L 53BP1 can limit sister-chromatid rupture and rearrangements driven by a distinct ultrafine DNA bridging-breakage process. Nat. Commun. 2018; 9:677. 29445165 10.1038/s41467-018-03098-y PMC5813243

[23] Xu Y. , Ning S., Wei Z., Xu R., Xu X., Xing M., Guo R., Xu D 53BP1 and BRCA1 control pathway choice for stalled replication restart. Elife. 2017; 6:e30523. 29106372 10.7554/eLife.30523 PMC5683755

[24] Her J. , Ray C., Altshuler J., Zhang H., Bunting S.F. 53BP1 Mediates ATR-Chk1 signaling and protects replication forks under conditions of replication stress. Mol. Cell. Biol. 2018; 38:e00472-17. 29378830 10.1128/MCB.00472-17 PMC5879462

[25] Villa M. , Bonetti D., Carraro M., Longhese M.P. Rad9/53BP1 protects stalled replication forks from degradation in Mec1/ATR-defective cells. EMBO Rep. 2018; 19:351–367. 29301856 10.15252/embr.201744910 PMC5797966

[26] Pinkosky S.L. , Groot P.H.E., Lalwani N.D., Steinberg G.R. Targeting ATP-Citrate lyase in hyperlipidemia and metabolic disorders. Trends Mol. Med. 2017; 23:1047–1063. 28993031 10.1016/j.molmed.2017.09.001

[27] Zaidi N. , Swinnen J.V., Smans K. ATP-citrate lyase: a key player in cancer metabolism. Cancer Res. 2012; 72:3709–3714. 22787121 10.1158/0008-5472.CAN-11-4112

[28] Wellen K.E. , Hatzivassiliou G., Sachdeva U.M., Bui T.V., Cross J.R., Thompson C.B. ATP-citrate lyase links cellular metabolism to histone acetylation. Science. 2009; 324:1076–1080. 19461003 10.1126/science.1164097 PMC2746744

[29] Fan F. , Williams H.J., Boyer J.G., Graham T.L., Zhao H., Lehr R., Qi H., Schwartz B., Raushel F.M., Meek T.D. On the catalytic mechanism of human ATP citrate lyase. Biochemistry. 2012; 51:5198–5211. 22657152 10.1021/bi300611s

[30] Wei J. , Leit S., Kuai J., Therrien E., Rafi S., Harwood H.J. Jr, DeLaBarre B., Tong L An allosteric mechanism for potent inhibition of human ATP-citrate lyase. Nature. 2019; 568:566–570. 30944472 10.1038/s41586-019-1094-6

[31] Adam K. , Ning J., Reina J., Hunter T. NME/NM23/NDPK and histidine phosphorylation. Int. J. Mol. Sci. 2020; 21:5848. 10.3390/ijms21165848 PMC7461546 32823988

[32] Wagner P.D. , Vu N.D Phosphorylation of ATP-citrate lyase by nucleoside diphosphate kinase. J. Biol. Chem. 1995; 270:21758–21764. 7665595 10.1074/jbc.270.37.21758

[33] Trapnell C. , Pachter L., Salzberg S.L. TopHat: discovering splice junctions with RNA-Seq. Bioinformatics. 2009; 25:1105–1111. 19289445 10.1093/bioinformatics/btp120 PMC2672628

[34] O’Leary N.A. , Wright M.W., Brister J.R., Ciufo S., Haddad D., McVeigh R., Rajput B., Robbertse B., Smith-White B., Ako-Adjei D. et al. Reference sequence (RefSeq) database at NCBI: current status, taxonomic expansion, and functional annotation. Nucleic. Acids. Res. 2016; 44:D733–D745. 26553804 10.1093/nar/gkv1189 PMC4702849

[35] Bray N.L. , Pimentel H., Melsted P., Pachter L. Near-optimal probabilistic RNA-seq quantification. Nat. Biotechnol. 2016; 34:525–527. 27043002 10.1038/nbt.3519

[36] Love M.I. , Huber W., Anders S. Moderated estimation of fold change and dispersion for RNA-seq data with DESeq2. Genome Biol. 2014; 15:550. 25516281 10.1186/s13059-014-0550-8 PMC4302049

[37] Srere P.A. The citrate cleavage enzyme. I. Distribution and purification. J. Biol. Chem. 1959; 234:2544–2547. 13833535

[38] Panier S. , Boulton S.J. Double-strand break repair: 53BP1 comes into focus. Nat. Rev. Mol. Cell Biol. 2014; 15:7–18. 24326623 10.1038/nrm3719

[39] Zhao S. , Torres A., Henry R.A., Trefely S., Wallace M., Lee J.V., Carrer A., Sengupta A., Campbell S.L., Kuo Y.M. et al. ATP-Citrate lyase controls a Glucose-to-Acetate metabolic switch. Cell Rep. 2016; 17:1037–1052. 27760311 10.1016/j.celrep.2016.09.069 PMC5175409

[40] Sivanand S. , Rhoades S., Jiang Q., Lee J.V., Benci J., Zhang J., Yuan S., Viney I., Zhao S., Carrer A. et al. Nuclear Acetyl-CoA production by ACLY promotes homologous recombination. Mol. Cell. 2017; 67:252–265. 28689661 10.1016/j.molcel.2017.06.008 PMC5580398

[41] Tuhackova Z. , Krivanek J. GTP, a nonsubstrate of ATP citrate lyase, is a phosphodonor for the enzyme histidine autophosphorylation. Biochem. Biophys. Res. Commun. 1996; 218:61–66. 8573177 10.1006/bbrc.1996.0012

[42] Sun T. , Hayakawa K., Bateman K.S., Fraser M.E. Identification of the citrate-binding site of human ATP-citrate lyase using X-ray crystallography. J. Biol. Chem. 2010; 285:27418–27428. 20558738 10.1074/jbc.M109.078667 PMC2930740

[43] Yang X. , Xu B., Mulvey B., Evans M., Jordan S., Wang Y.D., Pagala V., Peng J., Fan Y., Patel A. et al. Differentiation of human pluripotent stem cells into neurons or cortical organoids requires transcriptional co-regulation by UTX and 53BP1. Nat. Neurosci. 2019; 22:362–373. 30718900 10.1038/s41593-018-0328-5 PMC6511450

[44] Marzluff W.F. , Koreski K.P. Birth and death of histone mRNAs. Trends Genet. 2017; 33:745–759. 28867047 10.1016/j.tig.2017.07.014 PMC5645032

[45] Marzluff W.F. , Wagner E.J., Duronio R.J. Metabolism and regulation of canonical histone mRNAs: life without a poly(A) tail. Nat. Rev. Genet. 2008; 9:843–854. 18927579 10.1038/nrg2438 PMC2715827

[46] Gruber J.J. , Olejniczak S.H., Yong J., La Rocca G., Dreyfuss G., Thompson C.B. Ars2 promotes proper replication-dependent histone mRNA 3′ end formation. Mol. Cell. 2012; 45:87–98. 22244333 10.1016/j.molcel.2011.12.020 PMC3269315

[47] Kohn M. , Ihling C., Sinz A., Krohn K., Huttelmaier S. The Y3** ncRNA promotes the 3′ end processing of histone mRNAs. Genes Dev. 2015; 29:1998–2003. 26443846 10.1101/gad.266486.115 PMC4604341

[48] Youngblood B.A. , Grozdanov P.N., MacDonald C.C. CstF-64 supports pluripotency and regulates cell cycle progression in embryonic stem cells through histone 3′ end processing. Nucleic. Acids. Res. 2014; 42:8330–8342. 24957598 10.1093/nar/gku551 PMC4117776

[49] Sullivan K.D. , Mullen T.E., Marzluff W.F., Wagner E.J. Knockdown of SLBP results in nuclear retention of histone mRNA. RNA. 2009; 15:459–472. 19155325 10.1261/rna.1205409 PMC2657014

[50] Iwabuchi K. , Li B., Massa H.F., Trask B.J., Date T., Fields S. Stimulation of p53-mediated transcriptional activation by the p53-binding proteins, 53BP1 and 53BP2. J. Biol. Chem. 1998; 273:26061–26068. 9748285 10.1074/jbc.273.40.26061

[51] Clayton A.L. , Hazzalin C.A., Mahadevan L.C. Enhanced histone acetylation and transcription: a dynamic perspective. Mol. Cell. 2006; 23:289–296. 16885019 10.1016/j.molcel.2006.06.017

[52] Kurdistani S.K. , Tavazoie S., Grunstein M. Mapping global histone acetylation patterns to gene expression. Cell. 2004; 117:721–733. 15186774 10.1016/j.cell.2004.05.023

[53] Zhao X. , McKillop-Smith S., Muller B. The human histone gene expression regulator HBP/SLBP is required for histone and DNA synthesis, cell cycle progression and cell proliferation in mitotic cells. J. Cell Sci. 2004; 117:6043–6451. 15546920 10.1242/jcs.01523

[54] Armstrong C. , Spencer S.L. Replication-dependent histone biosynthesis is coupled to cell-cycle commitment. Proc. Natl. Acad. Sci. U. S. A. 2021; 118:e2100178118. 34326254 10.1073/pnas.2100178118 PMC8346857

[55] Mejlvang J. , Feng Y., Alabert C., Neelsen K.J., Jasencakova Z., Zhao X., Lees M., Sandelin A., Pasero P., Lopes M. et al. New histone supply regulates replication fork speed and PCNA unloading. J. Cell Biol. 2014; 204:29–43. 24379417 10.1083/jcb.201305017 PMC3882791

[56] Alabert C. , Groth A. Chromatin replication and epigenome maintenance. Nat. Rev. Mol. Cell Biol. 2012; 13:153–167. 22358331 10.1038/nrm3288

[57] Groth A. , Ray-Gallet D., Quivy J.P., Lukas J., Bartek J., Almouzni G. Human asf1 regulates the flow of s phase histones during replicational stress. Mol. Cell. 2005; 17:301–311. 15664198 10.1016/j.molcel.2004.12.018

[58] Groth A. , Corpet A., Cook A.J., Roche D., Bartek J., Lukas J., Almouzni G. Regulation of replication fork progression through histone supply and demand. Science. 2007; 318:1928–1931. 18096807 10.1126/science.1148992

[59] Azzalin C.M. , Lingner J. The human RNA surveillance factor UPF1 is required for s phase progression and genome stability. Curr. Biol. 2006; 16:433–439. 16488880 10.1016/j.cub.2006.01.018

[60] Zimmermann M. , de Lange T. 53BP1: pro choice in DNA repair. Trends Cell Biol. 2014; 24:108–117. 24094932 10.1016/j.tcb.2013.09.003 PMC3946699

[61] Meeks-Wagner D. , Hartwell L.H. Normal stoichiometry of histone dimer sets is necessary for high fidelity of mitotic chromosome transmission. Cell. 1986; 44:43–52. 3510079 10.1016/0092-8674(86)90483-6

[62] Groth A. , Rocha W., Verreault A., Almouzni G. Chromatin challenges during DNA replication and repair. Cell. 2007; 128:721–733. 17320509 10.1016/j.cell.2007.01.030

[63] Shah S. , Carriveau W.J., Li J., Campbell S.L., Kopinski P.K., Lim H.W., Daurio N., Trefely S., Won K.J., Wallace D.C. et al. Targeting ACLY sensitizes castration-resistant prostate cancer cells to AR antagonism by impinging on an ACLY-AMPK-AR feedback mechanism. Oncotarget. 2016; 7:43713–43730. 27248322 10.18632/oncotarget.9666 PMC5190055

[64] Schroeder S. , Pendl T., Zimmermann A., Eisenberg T., Carmona-Gutierrez D., Ruckenstuhl C., Marino G., Pietrocola F., Harger A., Magnes C. et al. Acetyl-coenzyme A: a metabolic master regulator of autophagy and longevity. Autophagy. 2014; 10:1335–1337. 24904996 10.4161/auto.28919 PMC4203557

[65] Youn C.K. , Kim H.B., Wu T.T., Park S., Cho S.I., Lee J.H. 53BP1 contributes to regulation of autophagic clearance of mitochondria. Sci. Rep. 2017; 7:45290. 28345606 10.1038/srep45290 PMC5366885

[66] Kaygun H. , Marzluff W.F. Regulated degradation of replication-dependent histone mRNAs requires both ATR and upf1. Nat. Struct. Mol. Biol. 2005; 12:794–800. 16086026 10.1038/nsmb972

[67] Ideue T. , Adachi S., Naganuma T., Tanigawa A., Natsume T., Hirose T. U7 small nuclear ribonucleoprotein represses histone gene transcription in cell cycle-arrested cells. Proc. Natl. Acad. Sci. U. S. A. 2012; 109:5693–5698. 22451911 10.1073/pnas.1200523109 PMC3326466

[68] Pirngruber J. , Shchebet A., Schreiber L., Shema E., Minsky N., Chapman R.D., Eick D., Aylon Y., Oren M., Johnsen S.A. CDK9 directs H2B monoubiquitination and controls replication-dependent histone mRNA 3′-end processing. EMBO Rep. 2009; 10:894–900. 19575011 10.1038/embor.2009.108 PMC2726677

[69] Salzler H.R. , Davidson J.M., Montgomery N.D., Duronio R.J. Loss of the histone pre-mRNA processing factor stem-loop binding protein in drosophila causes genomic instability and impaired cellular proliferation. PLoS One. 2009; 4:e8168. 19997601 10.1371/journal.pone.0008168 PMC2781718

