# Supplementary material for: 53BP1-ACLY-SLBP-coordinated activation of replication-dependent histone biogenesis maintains genomic integrity
Source: Nucleic Acids Res. 2022 Jan 17;50(3):1465–83. doi: 10.1093/nar/gkab1300 (PMC8860602; doi:10.1093/nar/gkab1300)
Supplement: gkab1300_Supplemental_File [file gkab1300_supplemental_file.docx]

**53BP1-ACLY-SLBP-coordinated activation of replication-dependent histone biogenesis maintains genomic integrity**

14 supplementary figures

8 supplementary tables

**
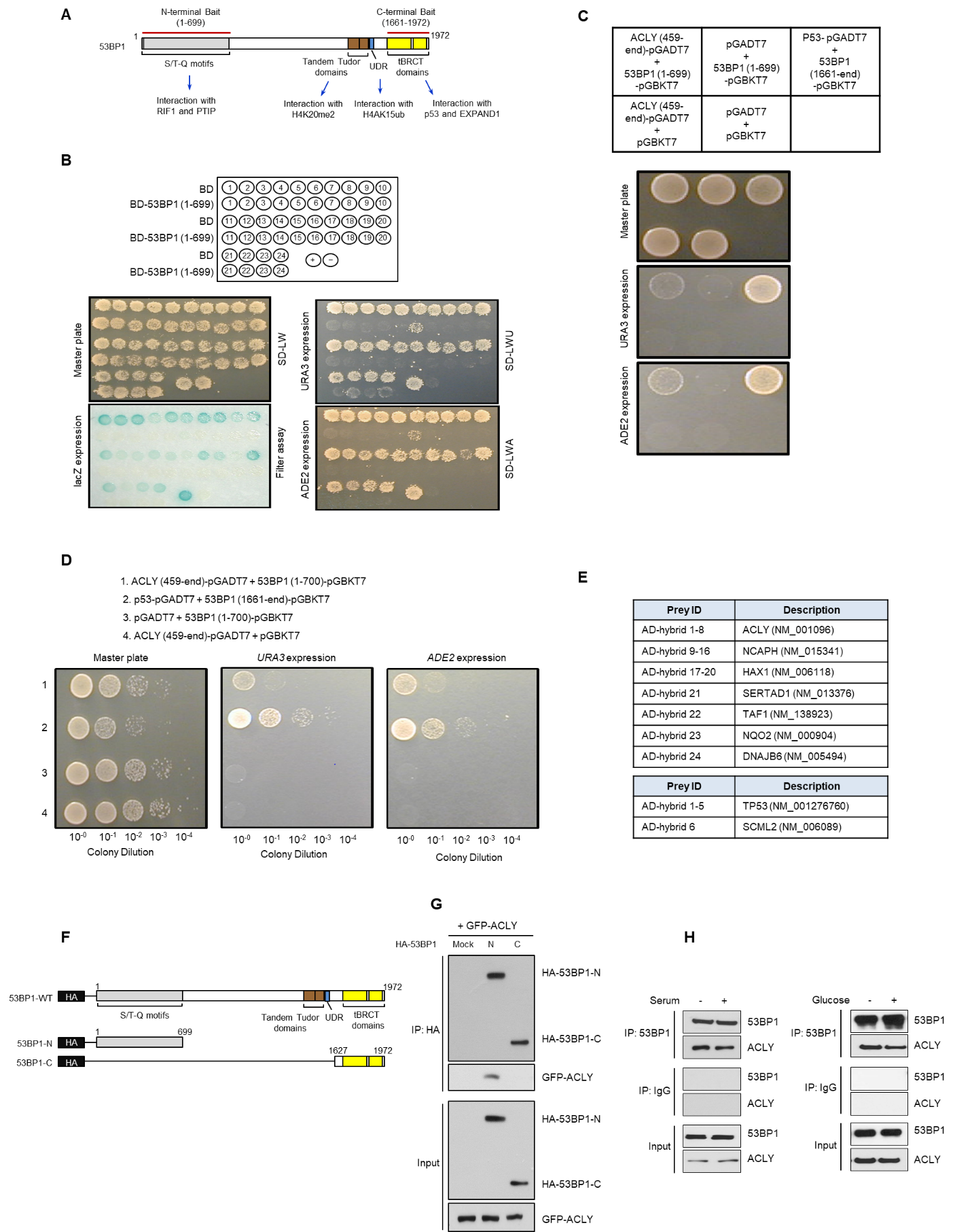
**

**Figure S1. Yeast two-hybrid screening identifies proteins that interact with either the N-terminal (1 – 699) or C-terminal (1661 – 1972) regions of 53BP1.**

(**A**) A schematic presentation of the functional domains of 53BP1 and proteins known to bind to each domain is shown. The S/T-Q motif (amino acids 1 to 699) and the tBRCT domain (amino acids 1661 to 1972) were used as a bait in the yeast two-hybrid experiment. (**B**) Yeast cells were transformed with the bait plasmid expressing either the GAL4-DNA binding domain (BD) (pGBKT7) or the GAL4-BD-fused S/T-Q motif and the prey plasmid expressing the GAL4 transcription activation domain-fused HeLa cDNA library. Transformed yeast cells were selected on the minimal media lacking leucine and tryptophan (SD-LW) to select for bait and prey plasmids, respectively. Specific interactions between two proteins were monitored by (i) the appearance of a visible blue color on the filter assay; (ii) the growth of colonies on the selective medium lacking leucine, tryptophan and uracil (SD-LWU); and (iii) the growth of colonies on the selective medium lacking leucine, tryptophan and adenine (SD-LWA). From this screening, 24 positive clones were identified. pGBKT7-53 (p53) and pGADT7-T (SV40 large T-antigen) served as a positive control for the protein-protein interaction. pGBKT7 and pGADT7 were used as the negative control. (**C**) Amino acids 459-1101 of ACLY are sufficient to interact with the N-terminal 1-699 amino acids of 53BP1. Combinations of truncated ACLY and truncated 53BP1, as indicated (upper table), were co-transformed into yeast cells (PBN204). Specific interactions between the two proteins were monitored as described in (b). p53-pGADT7 and 53BP1-BRCT (1661-end)-pGBKT7 served as a positive control, and pGADT7 and pGBKT7 were used as a negative control. (**D**) Combinations of truncated ACLY and truncated 53BP1, as indicated (upper list), were co-transformed into yeast cells (PBN204). The resulting colonies were grown overnight and spotted in ten-fold serial dilutions onto the indicated medium descried as (**B**). (**E**) A list of proteins identified in the screening that interacted with the N-terminal domain of 53BP1. The identity of each clone was determined by automated DNA sequencing and BLAST analysis. Yeast cells were co-transformed with the bait plasmid expressing either the GAL4-DNA BD (pGBKT7) or GAL4-BD-fused tBRCT domain (amino acids 1661 to 1972) and the prey plasmid expressing the GAL4 transcription activation domain-fused HeLa cDNA library. The screening was performed as described in (**B**). Two proteins were identified as potentially interacting with the C-terminal domain of 53BP1. (**F**) A schematic presentation of wild-type and the N- and C-terminal truncations of HA-53BP1 are shown. (**G**) HA-tagged versions of the N- or C-terminus of 53BP1 were co-transfected with GFP-ACLY into HEK293T cells. Coimmunoprecipitation was performed with an anti-HA antibody, and the immunoprecipitates were analyzed by western blots. Note that HA-tagged 53BP1-N migrates slower than expected in the gel. (**H**) HeLa cells were starved by serum depletion for 16 h and subsequently stimulated with 10% fetal bovine serum-containing media for 24 h (left). HeLa cells were cultured for 48 h in the presence or absence of 25 mM glucose (right). Total cell lysates were then immunoprecipitated with anti-53BP1 antibody and subjected to western blot analysis with anti-ACLY and anti-53BP1 antibodies.


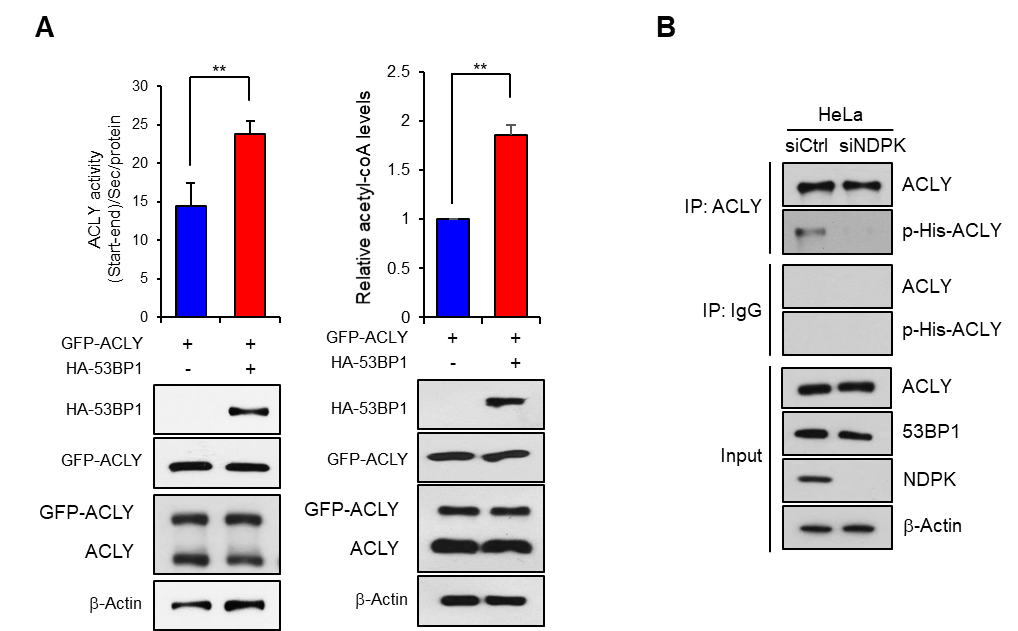


**Figure S2.** **53BP1 positively regulates ACLY activity**

(**A**) HA-53BP1 and GFP-ACLY were overexpressed inHEK293T cells in the indicated combinations. The ACLY enzyme activity (left) and the level of acetyl-CoA (right) were then measured after 48h. Data are presented as the mean (SD) from three independent experiments. ^**^*P* < 0.01, Student’s *t*-test. (**B**) HeLa cells were transfected with control or NDPK siRNA. 48 h after transfection, cell lysates were prepared for the immunoprecipitation (IP) using an anti-ACLY antibody and subjected to western blot analysis with the anti-ACLY or anti-phospho-histidine antibodies.


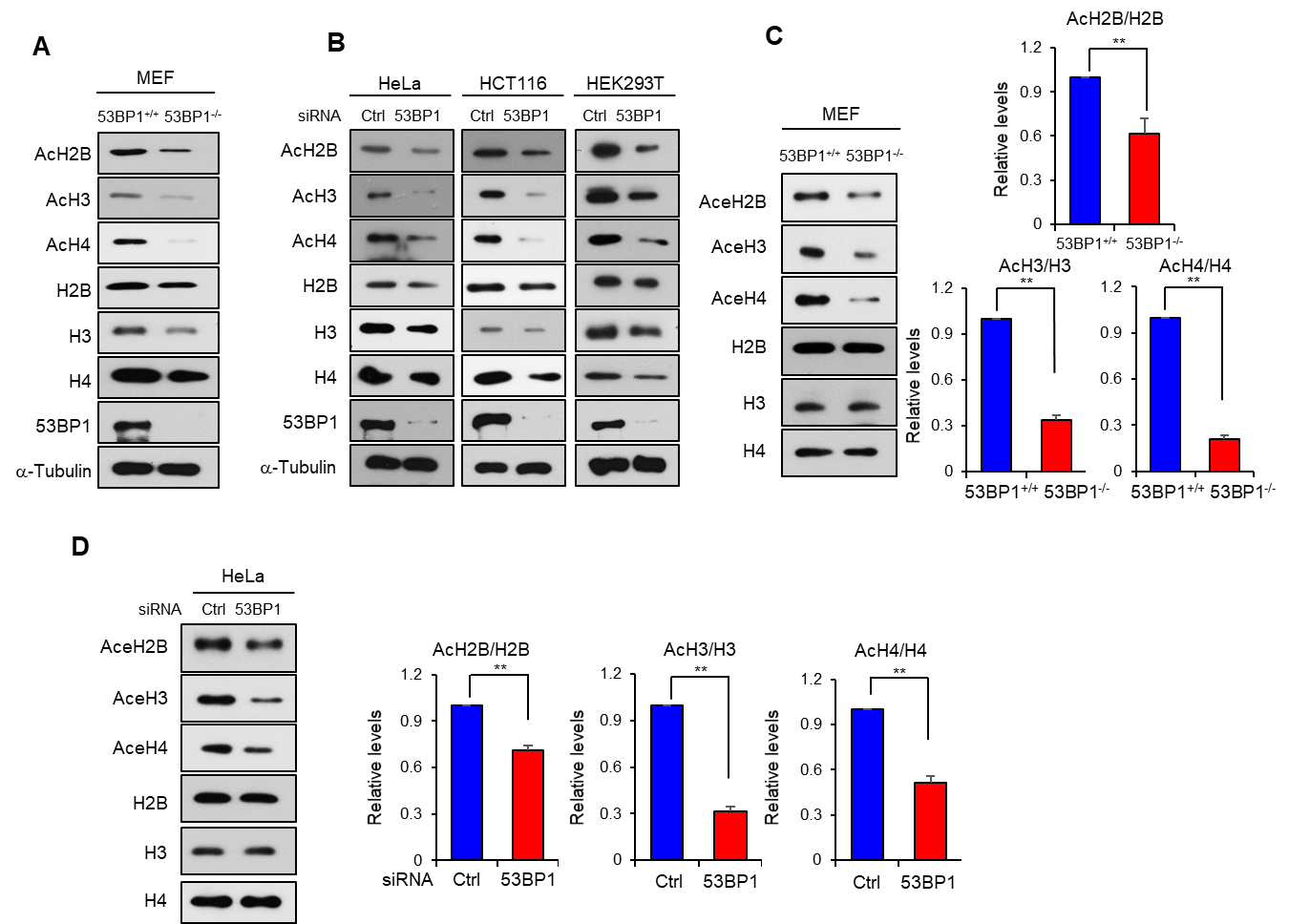


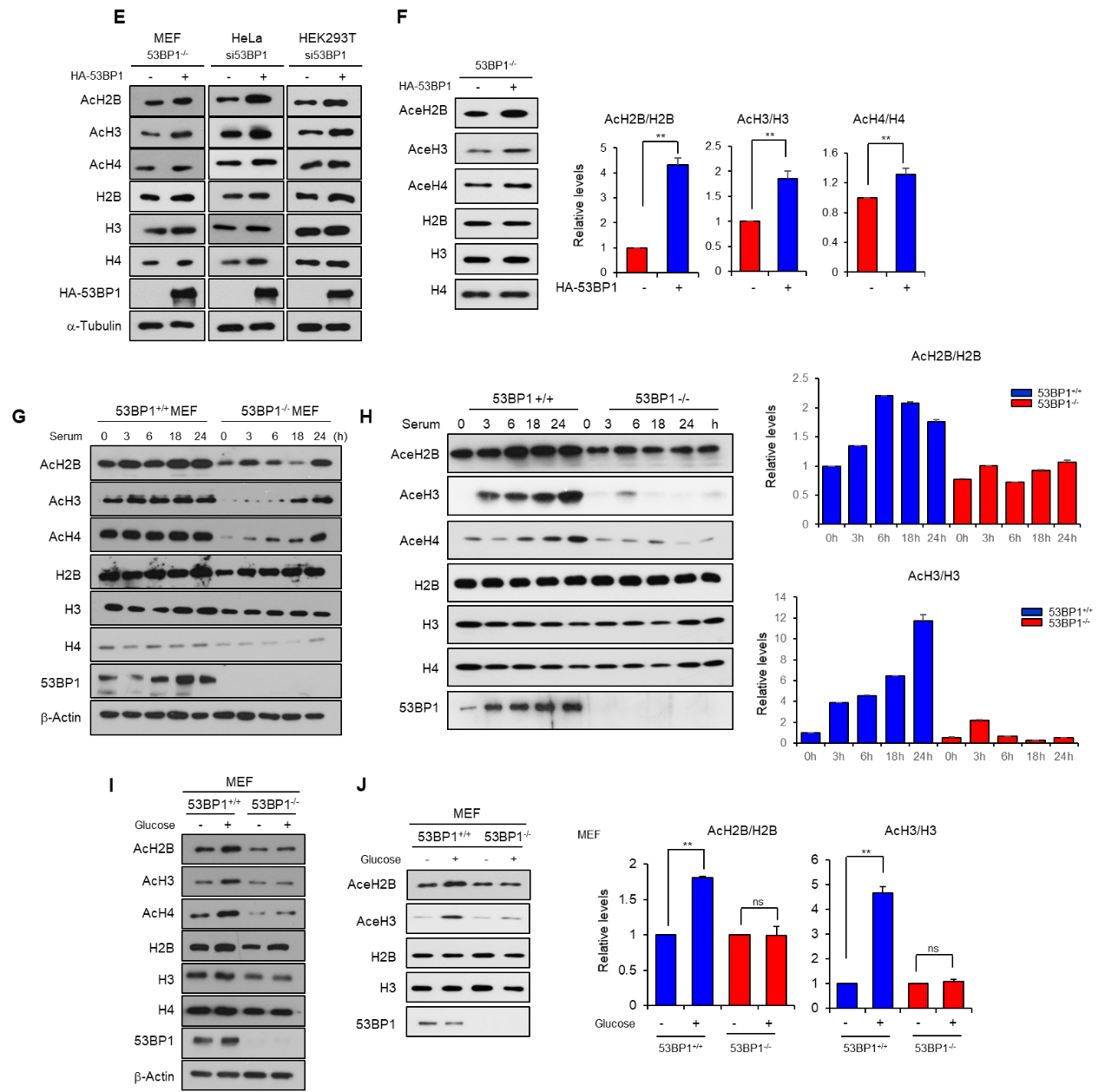


**Figure S3. 53BP1 regulates global histone acetylation.**

(**A**) Total cell extracts from 53BP1^+/+^ and 53BP1^−/−^ MEFs were analyzed for total and acetylated histones by western blotting using the indicated antibodies. (**B**) Western blot analyses of total and acetylated histones from control and 53BP1 knockdown in HeLa, HCT116, and HEK293T cells is shown. (**C and D**) The same quantitative western blot analyses shown in (A) were done by loading the same amount of histone proteins extracted from 53BP1^+/+^ and 53BP1^−/−^ MEFs (**C**), control and 53BP1-depleted HeLa cells (**D**). Data are presented as the mean (SD) from three independent experiments. ^**^*P* < 0.01, Student’s *t*-test. (**E**) HA-53BP1 was reconstituted in 53BP1^−/−^ MEFs, 53BP1-depleted HeLa and HEK293T cells for 48 h. Histone acetylation and total amounts of histones were analyzed using the indicated antibodies. (**F**) The same quantitative western blot analyses shown in (E) were done by loading the same amount of histone proteins extracted from 53BP1^−/−^ and HA-53BP1-reconstituted MEFs. Data are presented as the mean (SD) from three independent experiments. ^**^*P* < 0.01, Student’s *t*-test. (**G**) 53BP1^+/+^ and 53BP1^−/−^ MEFs were starved by serum depletion for 16 h and subsequently stimulated with 10% fetal bovine serum for the indicated time points. Total cell lysates were analyzed for total and acetylated histones by western blotting using the indicated antibodies. (**H**) The same amount of histone proteins extracted from the cells described in (**G**) was loaded in each lane and total and acetylated histones were analyzed by western blotting using the indicated antibodies. Data are presented as the mean (SD) from three independent experiments. (**I**) 53BP1^+/+^ and 53BP1^−/−^ MEFs were cultured for 48 h in the presence or absence of 25 mM glucose. Total cell lysates were analyzed by western blotting using the indicated antibodies. (**J**) The same amount of histone proteins extracted from the cells described in (**I**) was loaded in each lane and total and acetylated histones were analyzed by western blotting using the indicated antibodies. The results are shown as the mean ± SD (n = 3), ^**^*P* < 0.01. ns, not significant, Student’s *t*-test.


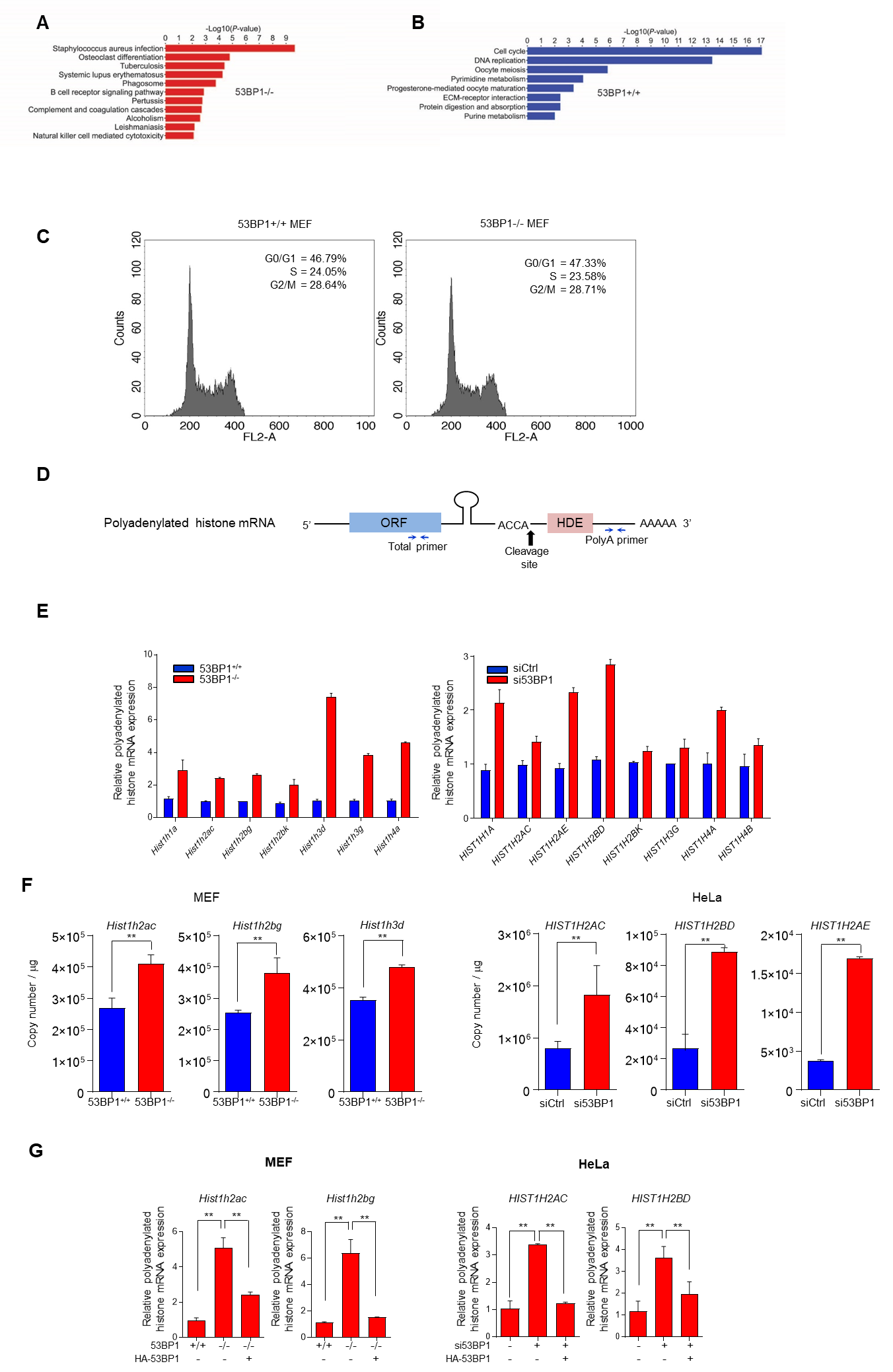


**Figure S4. The effect of 53BP1 on the level of total and polyadenylated histone mRNAs in 53BP1- deficient MEFs or 53BP1-depleted HeLa cells is shown**.

(**A**,**B**) Kyoto Encyclopedia of Genes and Genomes (KEGG) pathway analysis of genes for differential gene expression between 53BP1^-/-^ and 53BP1^+/+^ MEFs. **(C)** Cell cycle distribution in 53BP1^+/+^ and 53BP1^−/−^ MEFs. (**D)** A schematic of polyadenylated (top) or processed (bottom) histone mRNA is shown. The open reading frame (ORF), stem-loop, the cleavage site, and histone downstream element (HDE) regions are indicated. The position of RT-qPCR primers used to detect total (polyadenylated + fully processed) or polyadenylated histone mRNAs are shown. (**E**) Total RNAs from 53BP1^+/+^ and 53BP1^−/−^MEFs (left) or control siRNA- and 53BP1 siRNA-transfected HeLa cells (right) were analyzed for the polyadenylated histone mRNAs by RT-qPCR using oligo-dT-priming. The results are shown as the mean ± SD (n = 3). (**F**) Absolute quantitation of polyadenylated histone transcripts on select histone genes was conducted using 53BP1^+/+^, 53BP1^−/−^MEFs and 53BP1-depleted HeLa cells by RT-qPCR. The results are shown as the mean ± SD (n = 3), ^**^*P* < 0.01, Student’s *t*-test. (**G**) RT-qPCR analysis of polyadenylated *HIST1H2AC* and *HIST1H2BG/D* mRNAs in 53BP1^+/+^, 53BP1^−/−^, and 53BP1-reconstituted 53BP1^−/−^ MEFs, or control, 53BP1 depleted-HeLa cells, and 53BP1-depleted HeLa cells reconstituted with 53BP1. The results are shown as the mean ± SD (n = 3), ^**^*P* < 0.01, Student’s *t*-test.


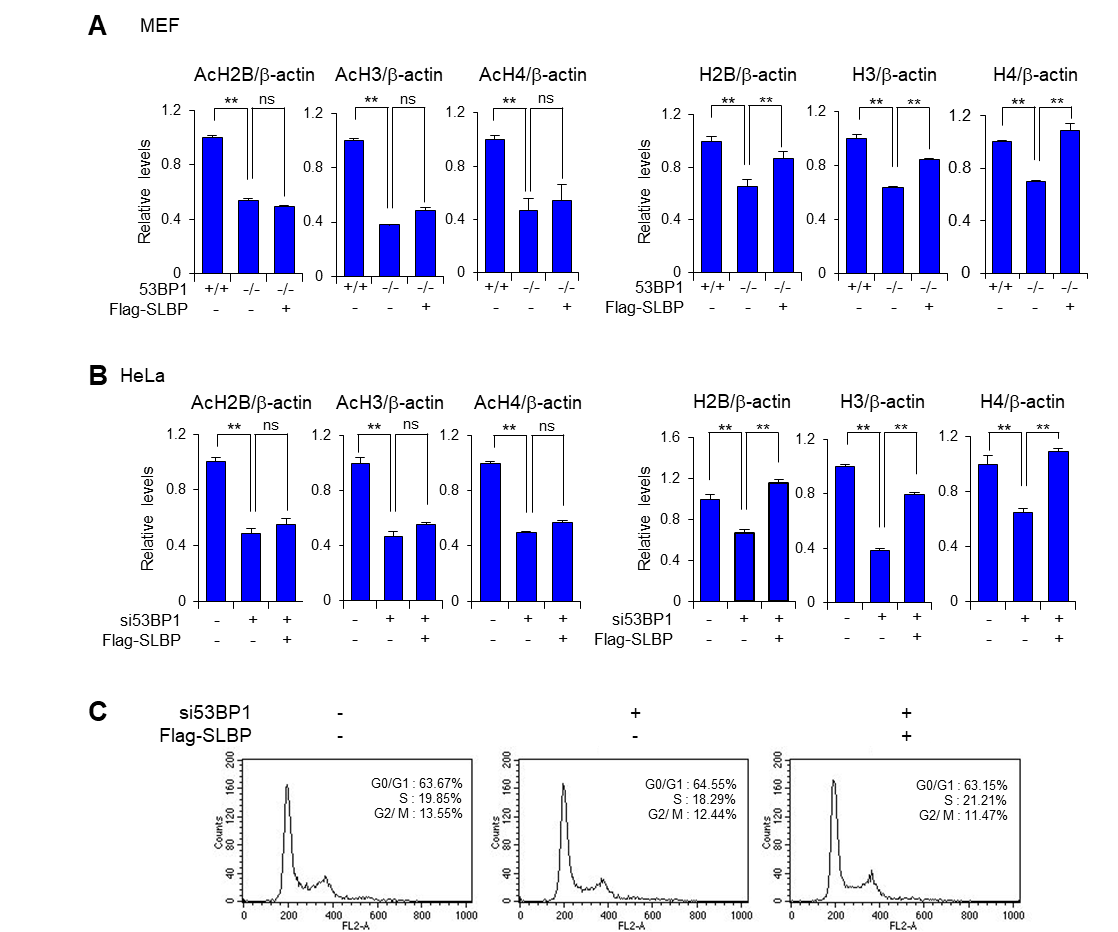


**Figure S5. The effect of overexpression of SLBP on cell cycle.**

(**A**, **B**) Densitometric analysis of the total and acetylated histones H2B, H3 and H4 in 53BP1^+/+^MEFs, 53BP1^−/−^MEFs, and SLBP-overexpressing 53BP1^−/−^ MEFs (**A**) and control HeLa, 53BP1-depleted HeLa, and SLBP-reconstituted 53BP1-depleted HeLa cells (**B**). Values are represented as the mean (SD) from three independent experiments. ***P* < 0.01. ns, not significant, Student’s *t*-test. (**C**) Control, 53BP1-depleted, and 53BP1-depleted HeLa cells reconstituted with SLBP were stained with propidium iodide and the DNA contents were then analyzed by flow cytometry.

**
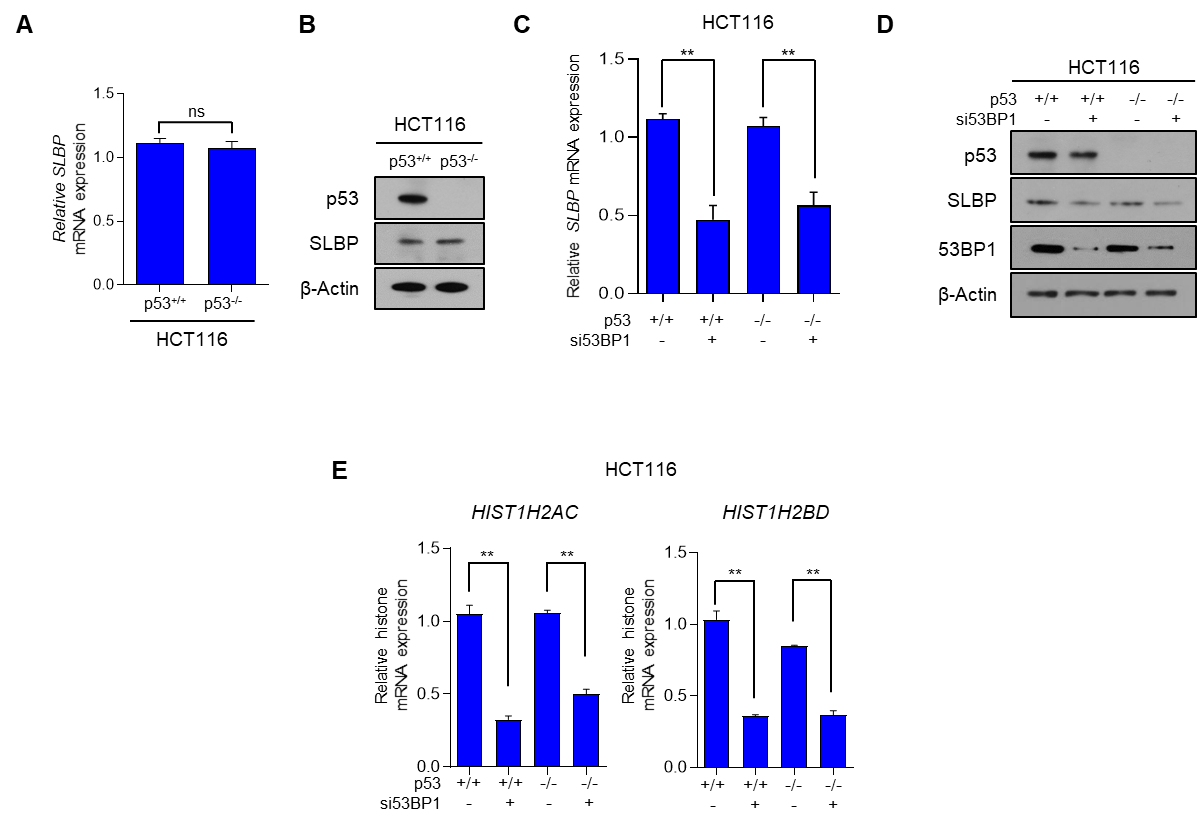
**

**Figure S6. p53 does not contribute to 53BP1-mediated regulation of SLBP expression and histone polyadenylation.**

**(A)** RT-qPCR analysis of *SLBP* mRNA expression in HCT116 p53^+/+^ and HCT116 p53^−/−^ cells. The results are shown as the mean ± SD (n = 3). ns, not significant, Student’s *t*-test. **(B)** Western blot analysis of SLBP expression in HCT116 p53^+/+^ and HCT116 p53^−/−^ cells. **(C)** RT-qPCR analysis of *SLBP* expression in 53BP1 siRNA-transfected HCT116 p53^+/+^ and HCT116 p53^−/−^ cells. The results are shown as the mean ± SD (n = 3), ^**^*P* < 0.01, Student’s *t*-test. **(D)** Western blot analysis of SLBP expression in HCT116 p53^+/+^ and p53^−/−^ cells with or without the knockdown of 53BP1. **(E)** RT-qPCR analysis of *Hist1h2ac* and *Hist1h2bD* mRNAs in HCT116 p53^+/+^ and p53^−/−^ cells with or without the knockdown of 53BP1. The results are shown as the mean ± SD (n = 3), ^**^*P* < 0.01, Student’s *t*-test.

**
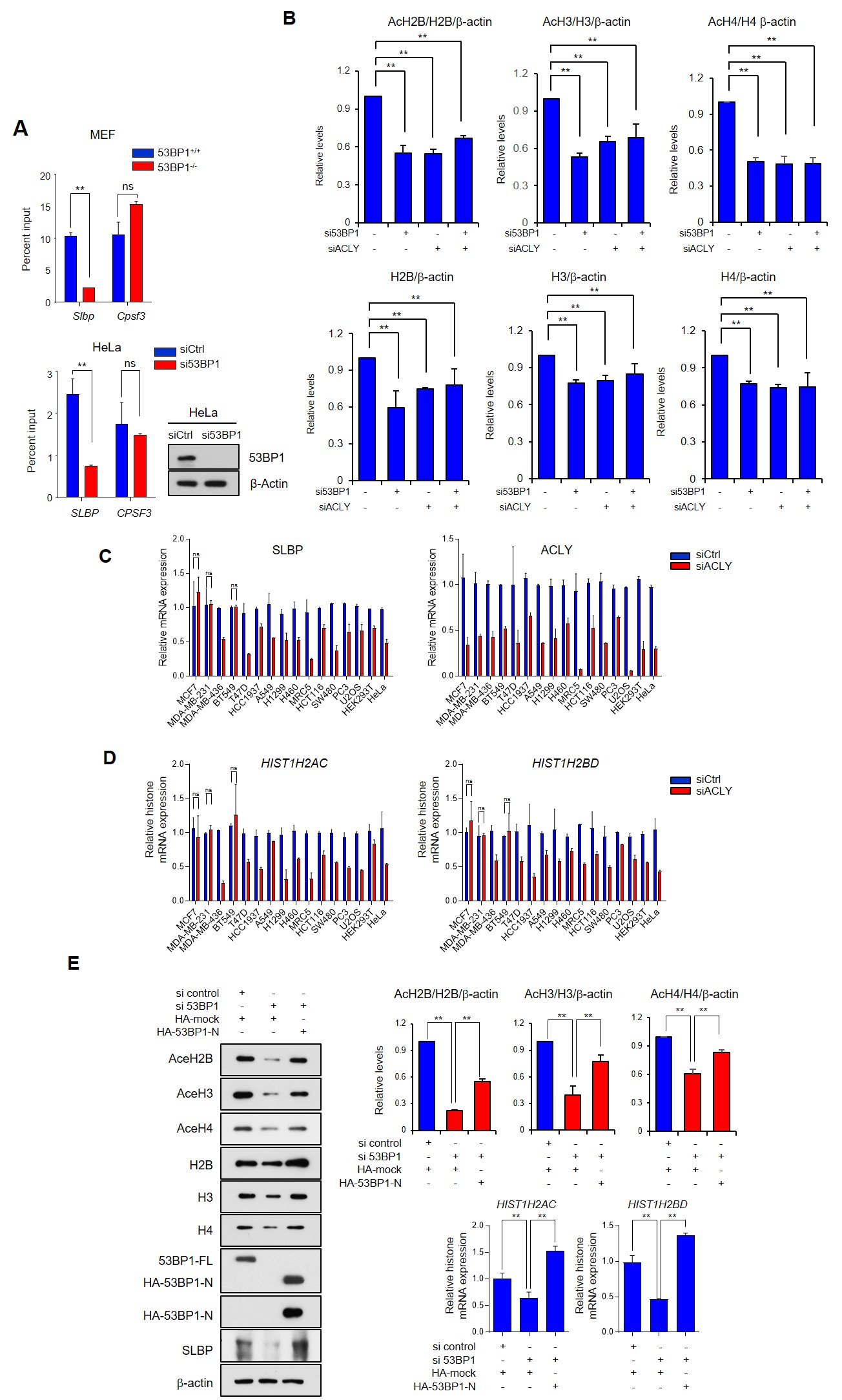
**

**
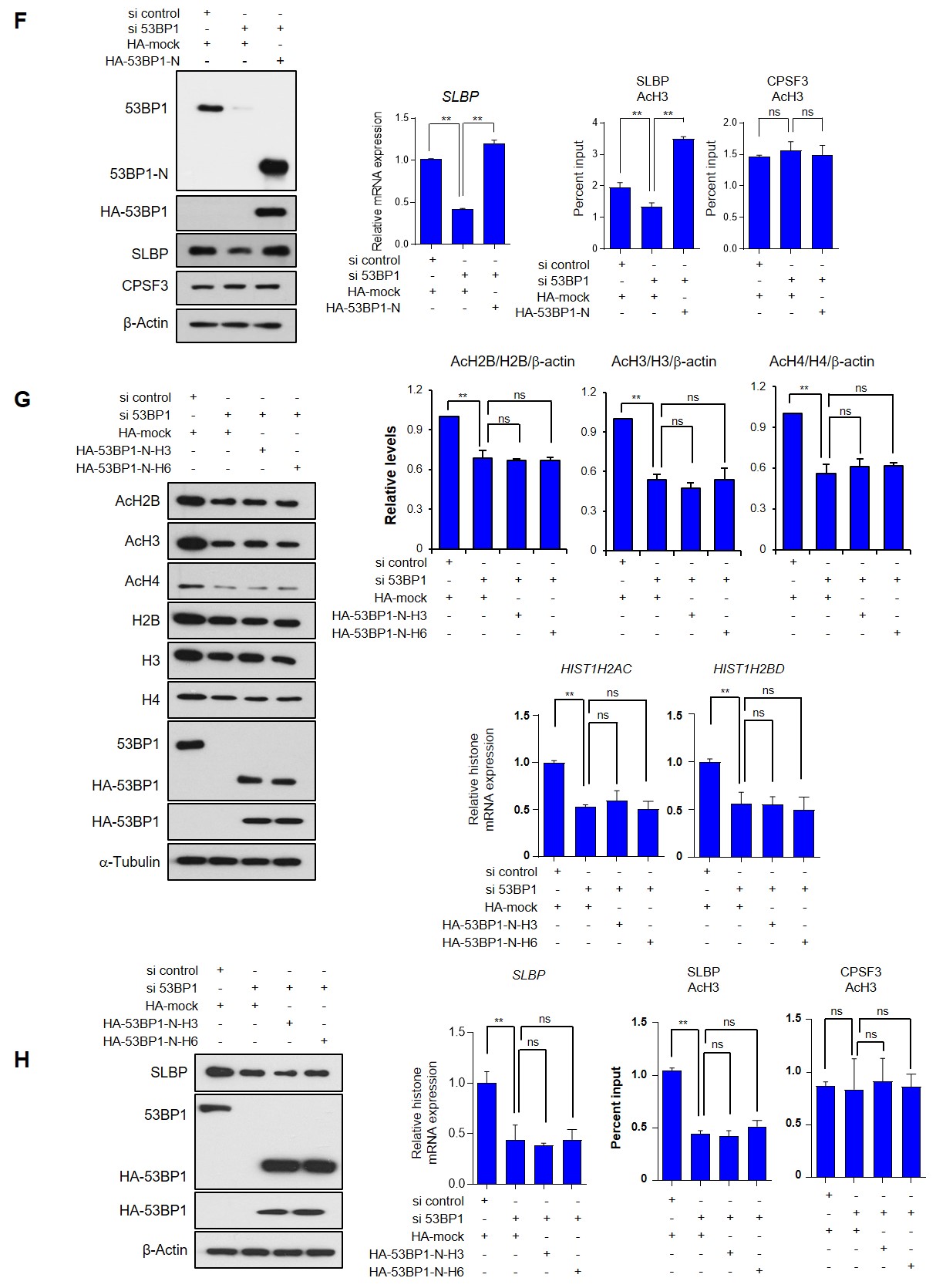
**

**Figure S7. The effect of 53BP1-mediated increase of ACLY activity on the expression levels of histone and SLBP.**

(**A**) Chromatin immunoprecipitation (ChIP)-qPCR analysis of the *SLBP* and *CPSF3* promoters in 53BP1^+/+^ and 53BP1^−/−^ MEFs (left) or control and 53BP1 siRNA-transfected HeLa cells (right) was performed using an antibody that binds acetylated histone H3 (Ac-H3). Data represent ChIP enrichment relative to input. The results are shown as the mean ± SD (n = 3), ^**^*P* < 0.01. ns, not significant, Student’s *t*-test. The results are shown as the mean ± SD (n = 3), ^**^*P* < 0.01. ns, not significant, Student’s *t*-test. (**B**) Densitometric analysis of total and acetylated histones H2B, H3 and H4 in control siRNA-, ACLY siRNA-, and 53BP1 siRNA-transfected HeLa cells is shown. The intensity of acetylated histones was normalized to that of the corresponding total histone levels. The results are shown as the mean ± SD (n = 3), ^**^*P* < 0.01, Student’s *t*-test. (**C**) The indicated normal and cancer human cells were transfected with control and ACLY siRNA. 48 h after transfection, the levels of SLBP and ACLY mRNA were analyzed by RT-qPCR. The results are shown as the mean ± SD (n = 3). ns, not significant, Student’s *t*-test. (**D**) Total RNAs from the control and ACLY siRNA-transfected cancer cells were analyzed by random-primed RT-qPCR for the expression of *HIST1H2AC* and *HIST1H2BD* mRNAs in ACLY-depleted HeLa cells. The results are shown as the mean ± SD (n = 3). ns, not significant, Student’s *t*-test. (**E**) The expression levels of total and acetylated histone, and *HIST1H2AC* and *HIST1H2BD* mRNAs in 53BP1-depleted HeLa cells reconstituted with control or HA-53BP1-N is shown. The results are shown as the mean ± SD (n = 3), ** *P* < 0.01, Student’s *t*-test. (**F**) The expression levels of SLBP protein and mRNA, and ChIP-qPCR analysis of the *SLBP* and *CPSF3* promoter in 53BP1-depleted HeLa cells reconstituted with HA-53BP1-N is shown. CPSF3 was used as a control. The results are shown as the mean ± SD (n = 3), ** *P* < 0.01, Student’s *t*-test. (**G**) The expression levels of total and acetylated histone, SLBP, and *HIST1H2AC* and *HIST1H2BD* mRNAs in 53BP1-depleted HeLa cells reconstituted with 53BP1-H3 or 53BP1-H6 is shown. The results are shown as the mean ± SD (n = 3), ** *P* < 0.01, Student’s *t*-test. (**H**) The expression levels of SLBP protein and mRNA, and ChIP qPCR analysis of the *SLBP* and *CPSF3* promoter in the same cells as described in (**E**) is shown. The results are shown as the mean ± SD (n = 3), ** *P* < 0.01, Student’s *t*-test.

**
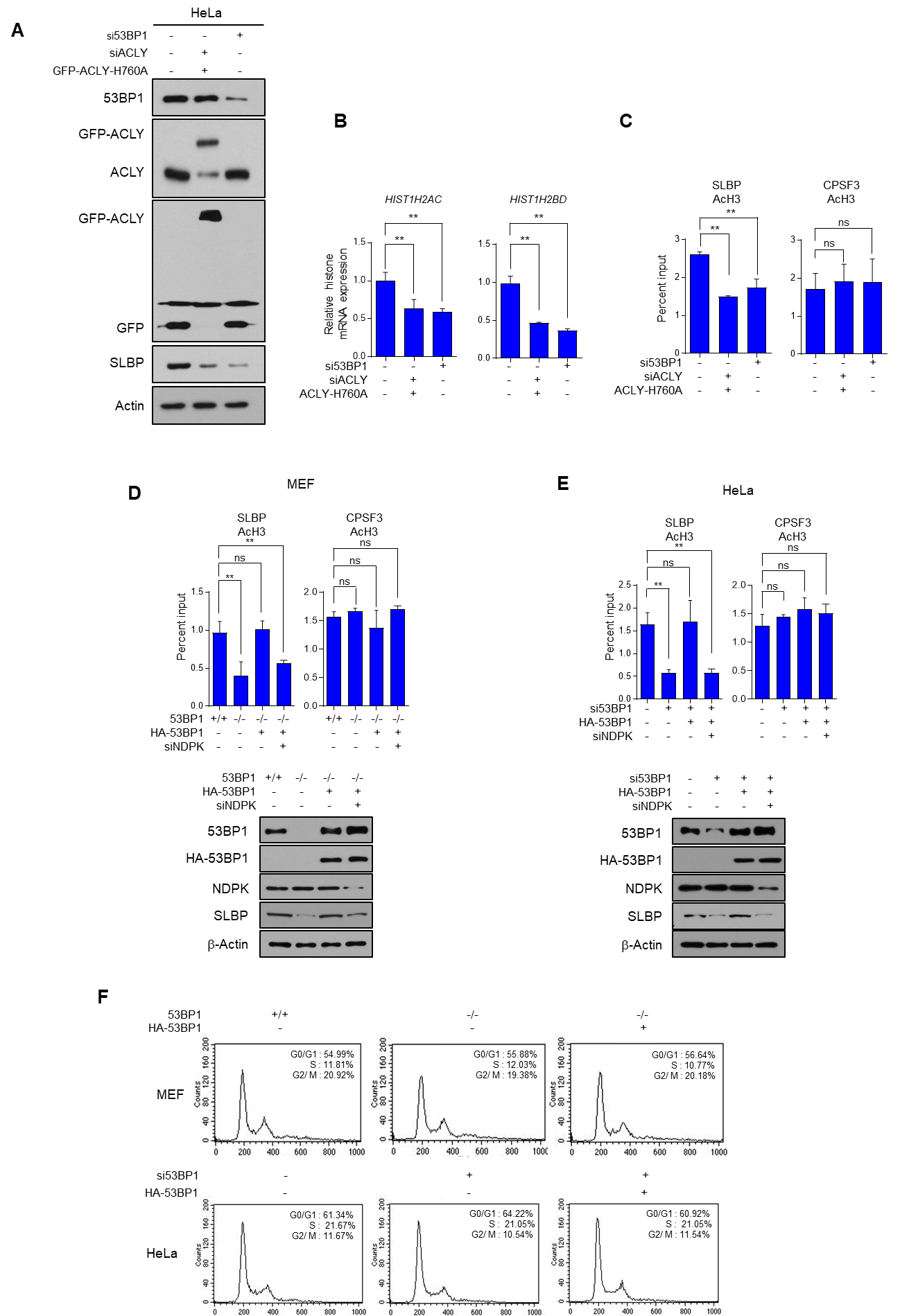
**

**
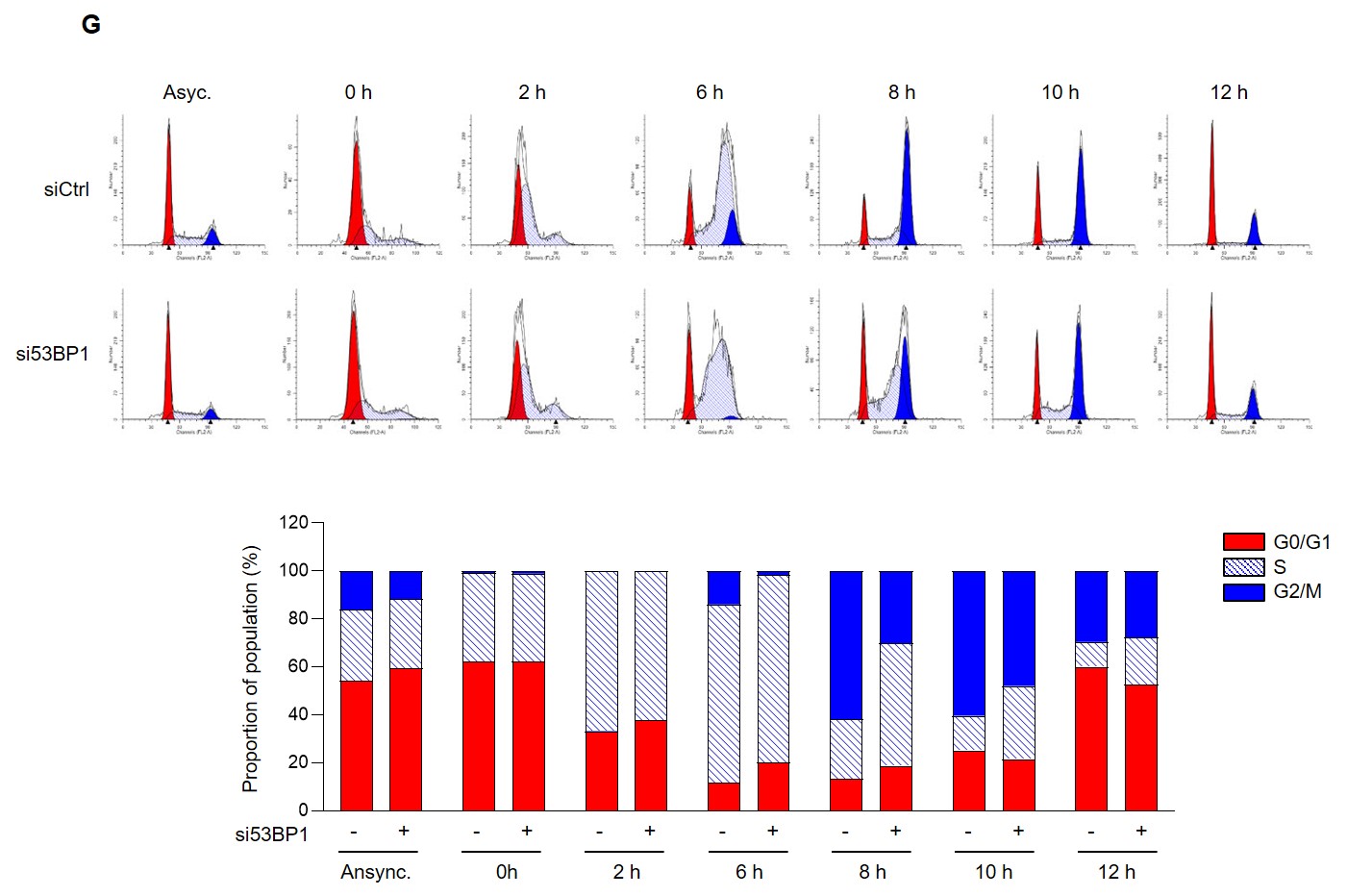
**

**Figure S8. 53BP1 upregulates the SLBP expression by NDPK-mediated ACLY phosphorylation.**

(**A**, **B**) The levels of SLBP protein (**A**) and mRNA (**B**) in control, 53BP1-depleted, ACLY-depleted HeLa cells with the reconstitution of GFP-ACLY-H760A. The results are shown as the mean ± SD (n = 3), ** *P* < 0.01, Student’s *t*-test. (**C**) Chromatin immunoprecipitation (ChIP)-qPCR analysis of the *SLBP* and *CPSF3* promoters in control, 53BP1-depleted, ACLY-depleted HeLa cells with the reconstitution of GFP-ACLY-H760A was performed using an antibody that binds acetylated histone H3 (Ac-H3). Data represent ChIP enrichment relative to input. The results are shown as the mean ± SD (n = 3), ^**^*P* < 0.01. ns, not significant, Student’s *t*-test. (**D, E**) ChIP-qPCR analysis of the *SLBP* and *CPSF3* promoters in 53BP1^+/+^ MEFs, 53BP1^−/−^ MEFs, HA-53BP1-reconstituted 53BP1^−/−^ MEFs, and HA-53BP1- reconstituted 53BP1^−/−^ MEFs transfected with NDPK siRNA (**D**) or control HeLa cells, 53BP1-depleted HeLa cells, HA-53BP1- reconstituted 53BP1-depleted HeLa cells, and HA-53BP1- reconstituted 53BP1-depleted HeLa cells transfected with NDPK siRNA (**E**). ChIP-qPCR was performed using an antibody that binds acetylated histone H3 (Ac-H3). Data represent ChIP enrichment relative to input. Western blot analyses of the expression of SLBP, NDPK, and 53BP1 are shown. The results are shown as the mean ± SD (n = 3), ^**^*P* < 0.01. ns, not significant, Student’s *t*-test. (**F**) Indicated MEFs (53BP1^+/+^MEFs, 53BP1^−/−^MEFs, and 53BP1-reconstituted 53BP1^−/−^ MEFs) and HeLa cells (control siRNA-, 53BP1 siRNA-transfected HeLa, and of 53BP1-reconstituted 53BP1-depleted HeLa) were stained with propidium iodide and the DNA contents were then analyzed by flow cytometry. (**G**) control siRNA- and 53BP1 siRNA-transfected HeLa cells were synchronized at S-phase by double thymidine block. After release, samples were collected at the indicated time points for cell cycle analysis for flow cytometry. The percentages of cells in G0/G1, S, or G2/M calculated by using ModiFit program are indicated.


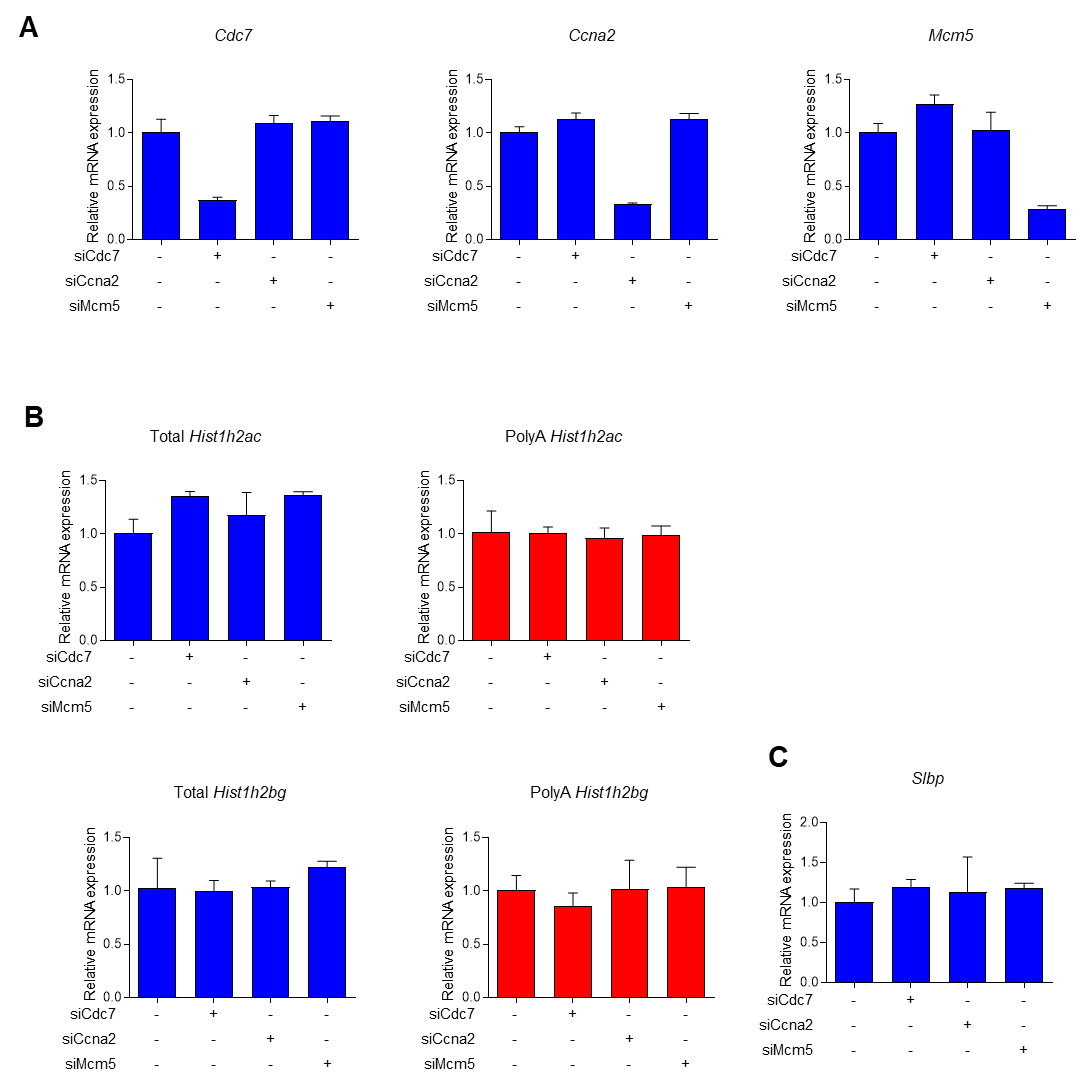


**Figure S9. Knockdown of cell cycle regulatory genes does not affect histone polyadenylation.**

(**A-C**) MEFs cells were transfected with control, Cdc7, Ccna2, or Mcm5 siRNAs, and the levels of Cdc7, Ccna2 and Mcm5 mRNA (A) and total and polyadenylated *Hist1h2ac* and *Hist1h2bg* mRNA (B) and SLBP mRNA (C) were analyzed by RT-qPCR. The results are shown as the mean ± SD (n = 3).


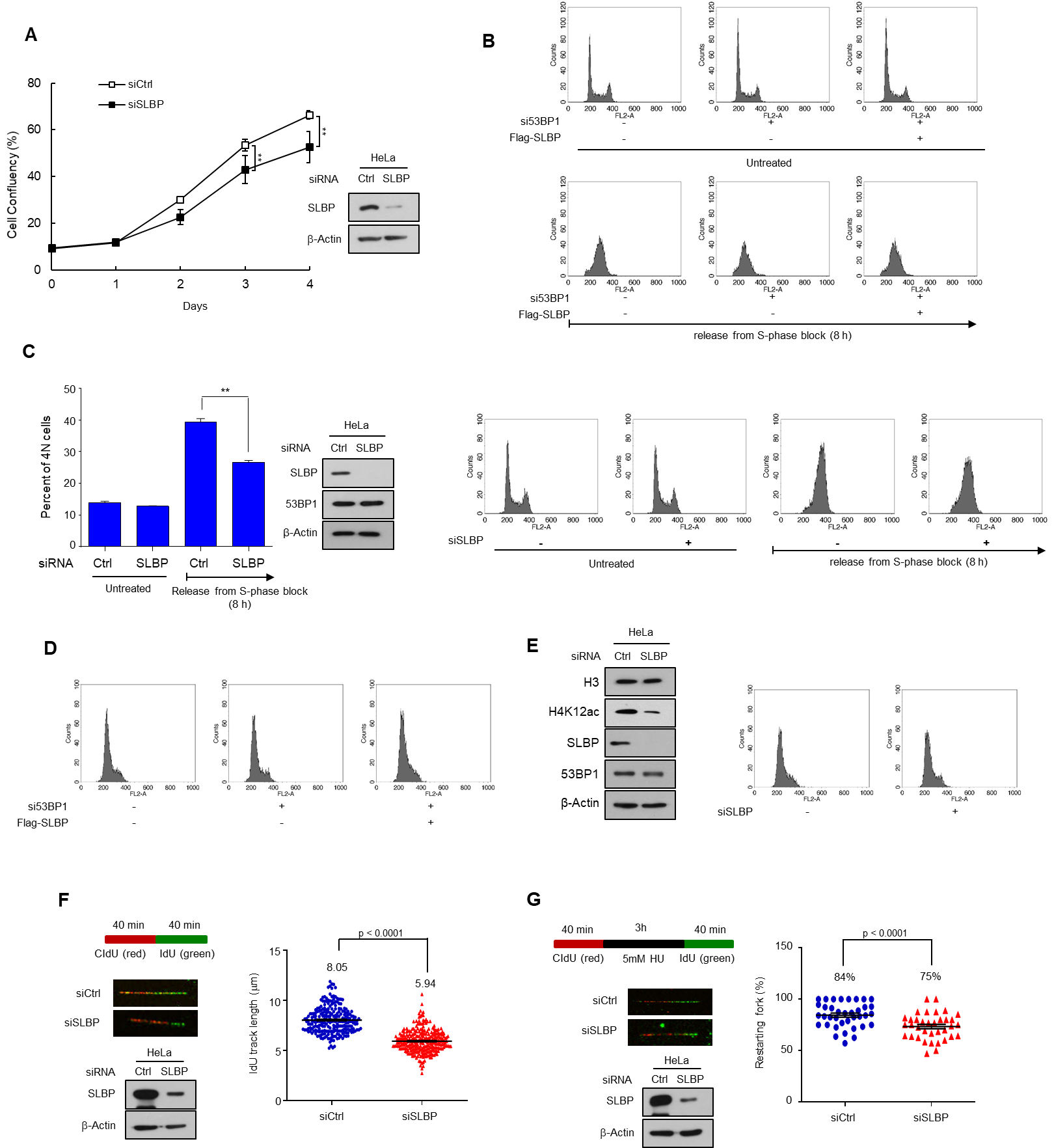


**Figure S10. The effect of an SLBP knockdown on cell proliferation, cell cycle progression in S-phase and the recovery of stalled replication forks.**

(**A**) HeLa cells were transfected with control siRNA or SLBP siRNA and cell proliferation was monitored by live cell imaging for 4 days. The results are shown as the mean ± SD (n = 3), ^**^*P* < 0.01, Student’s *t*-test. (**B**) Control, 53BP1-depleted, and 53BP1-depleted HeLa cells reconstituted with Flag-SLBP were treated with aphidicolin to arrest cells in S-phase. Then cells were incubated with colcemid-containing medium to release from the arrest and to trap in M-phase. Cell cycle profiles were measured by flow cytometry. (**C**) Control and SLBP-depleted HeLa cells were treated with aphidicolin to arrest cells in S-phase and then were released and trapped in M-phase (to measure 4N DNA content) by incubating in the colcemid-containing. Cell cycle profiles were monitored by flow cytometry analysis of propidium iodide-stained cells to measure the nuclear DNA content. The results are shown as the mean ± SD (n = 3), ^**^*P* < 0.01, Student’s *t*-test. (**D**) Control, 53BP1-depleted, and 53BP1-depleted HeLa cells reconstituted with Flag-SLBP were synchronized at S-phase through a two-step thymidine block. Cell cycle profiles were measured by flow cytometry. (**E**) Control or SLBP siRNA-transfected HeLa cells were synchronized at S-phase through a two-step thymidine block. Total cell extracts were then analyzed for H3, H2B and H4K12ac by western blot using the indicated antibodies. Cell cycle profiles were measured by flow cytometry. (**F**) Control and SLBP-depleted HeLa cells were treated with CIdU and IdU for 40 min each. DNA fibers were prepared and stained with CIdU (red) and IdU (green) antibodies and representative images of replication tracks are shown. The replication elongation rates were determined by measuring the IdU track length of CIdU-positive fibers. Representative images and quantification of mean track length are shown. Data represent mean ± SD (n = 3). *P* values between indicated samples were calculated using a Mann-Whitney test. (**G**) Control and SLBP-depleted HeLa cells were pulse-labeled with CIdU, treated with 5 mM HU for 2 h, and then released into IdU. DNA fibers were stained with antibodies recognizing IdU (red) and CIdU (green). Replication fork restart was measured by counting DNA fibers with contiguous IdU and CIdU tracks after exposure to HU and a representative image is shown. Quantification of mean track length is shown. Data represent mean ± SD (n = 3). *P* values between indicated samples were calculated using a Mann-Whitney test.


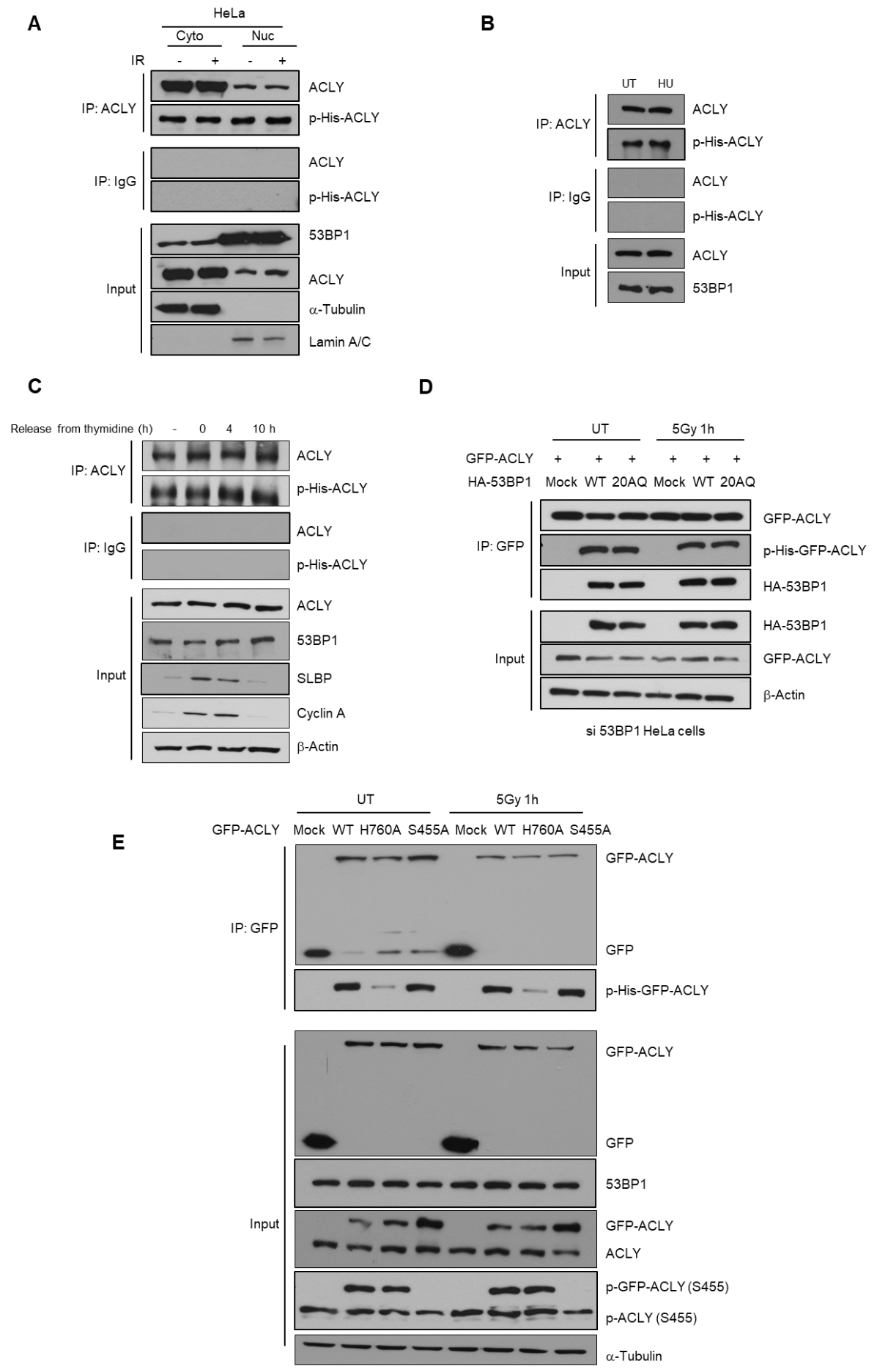


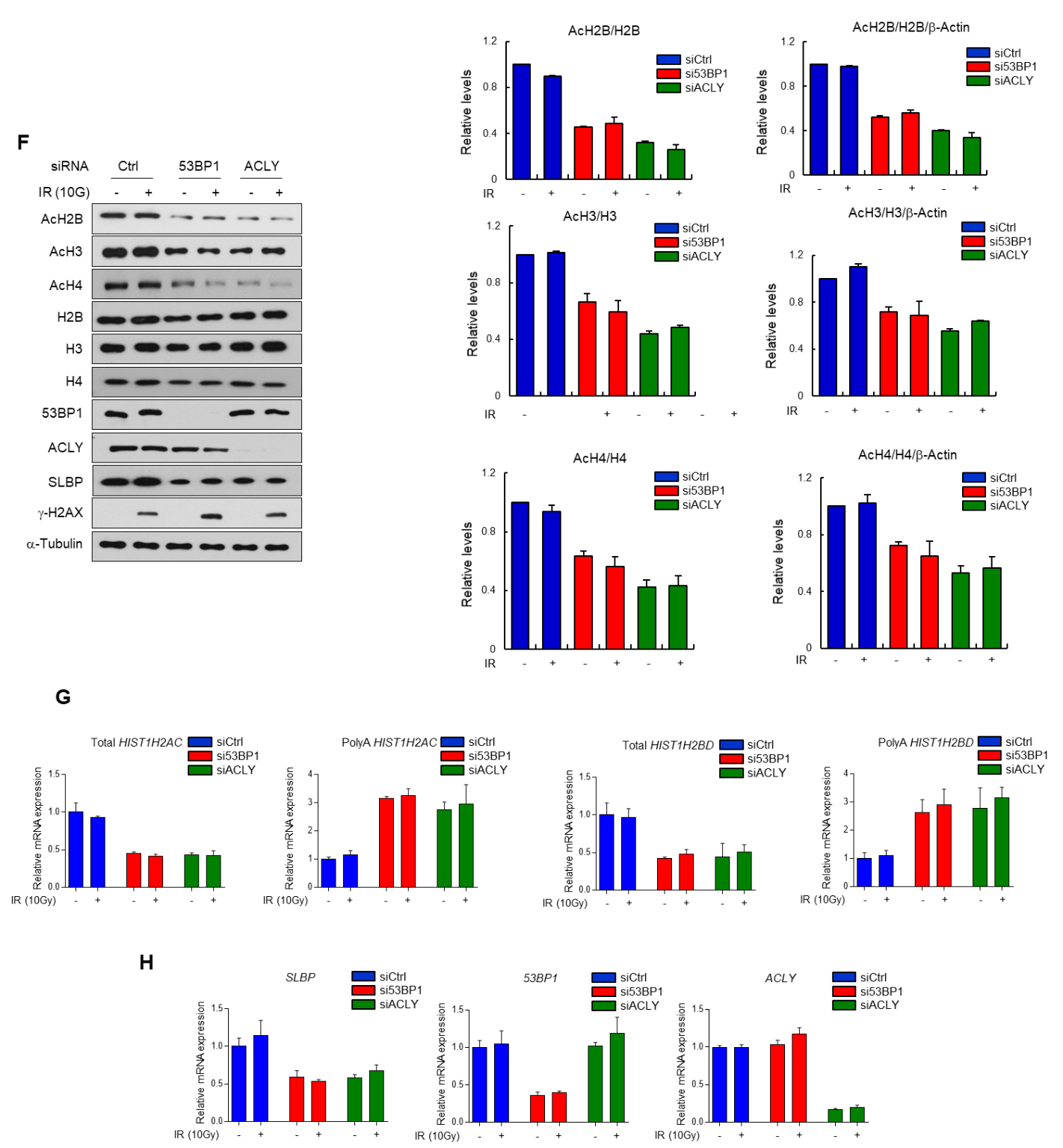


**Figure S11. 53BP1-mediated regulation of ACLY phosphorylation and histone biogenesis is independent of the DNA damage response**

(**A**) HeLa cells were treated with or without 5 Gy IR. 1h after IR treatment, cytosolic (Cyto) and nuclear (Nuc) faction were then immunoprecipitated with anti-ACLY antibody and subjected to western blot analysis with anti-phospho-histidine and anti-ACLY antibodies. (**B**) HeLa cells were treated with or without 3 mM HU for 3 h. Total cell lysates were then immunoprecipitated with anti-ACPY antibody and subjected to western blot analysis with anti-phospho-histidine and anti-ACLY antibodies. (**C**) HeLa cells were synchronized with double thymidine treatment to arrest the cell at G1/S boundary, and then released from double thymidine block. Total cellular lysates of collected cells at a indicated time point after released from double thymidine block were immunoprecipitated with anti-ACLY antibody. Immunoblot were performed with anti-phospho-histidine and anti-ACLY antibodies. (**D**) HeLa cells transfected with GFP-ACLY along with control Mock vector, HA-53BP1-WT, or HA-53BP1-20AQ were treated with or without 5 Gy of IR. 1h after IR treatment, cell lysates were immunoprecipitated with anti-GFP antibody and subjected to immunoblot analysis with anti-HA, anti-phospho-histidine and anti-GFP antibodies. (**E**) HeLa cells transfected with HA-53BP1 along with control Mock vector, GFP-ACLY1-WT, GFP-ACLY-H760A, or GFP-ACLY-S455A were treated with or without 5 Gy of IR. 1h after IR treatment, cell lysates were immunoprecipitated with anti-GFP antibody and subjected to immunoblot analysis with anti-HA, anti-phospho-histidine, and anti-GFP antibodies. (**F**) HeLa cells transfected with control (Ctrl), 53BP1, or ACLY siRNAs were treated with or without 10 Gy of IR. 1h after IR treatment, the amount of total and acetylated histones were analyzed using the indicated antibodies. Quantitation of acetylated histone was done by normalizing to total histone/α-tubulin. The results are shown as the mean ± SD (n = 3). ns, not significant, Student’s *t*-test. (**G**) HeLa cells transfected with control (Ctrl), 53BP1, or ACLY siRNAs were treated with or without 5 Gy IR. 1 h after IR treatment, the levels of total and polyadenylated *Hist1h2ac* and *Hist1h2bd* mRNA were analyzed by RT-qPCR. The results are shown as the mean ± SD (n = 3). ns, not significant, Student’s *t*-test. (**H**) HeLa cells transfected with control (Ctrl), 53BP1, or ACLY siRNAs were treated with or without 5 Gy IR. 1 h after IR treatment, the levels of SLBP, 53BP1, and ACLY were analyzed by RT-qPCR. The results are shown as the mean ± SD (n = 3). ns, not significant, Student’s *t*-test.


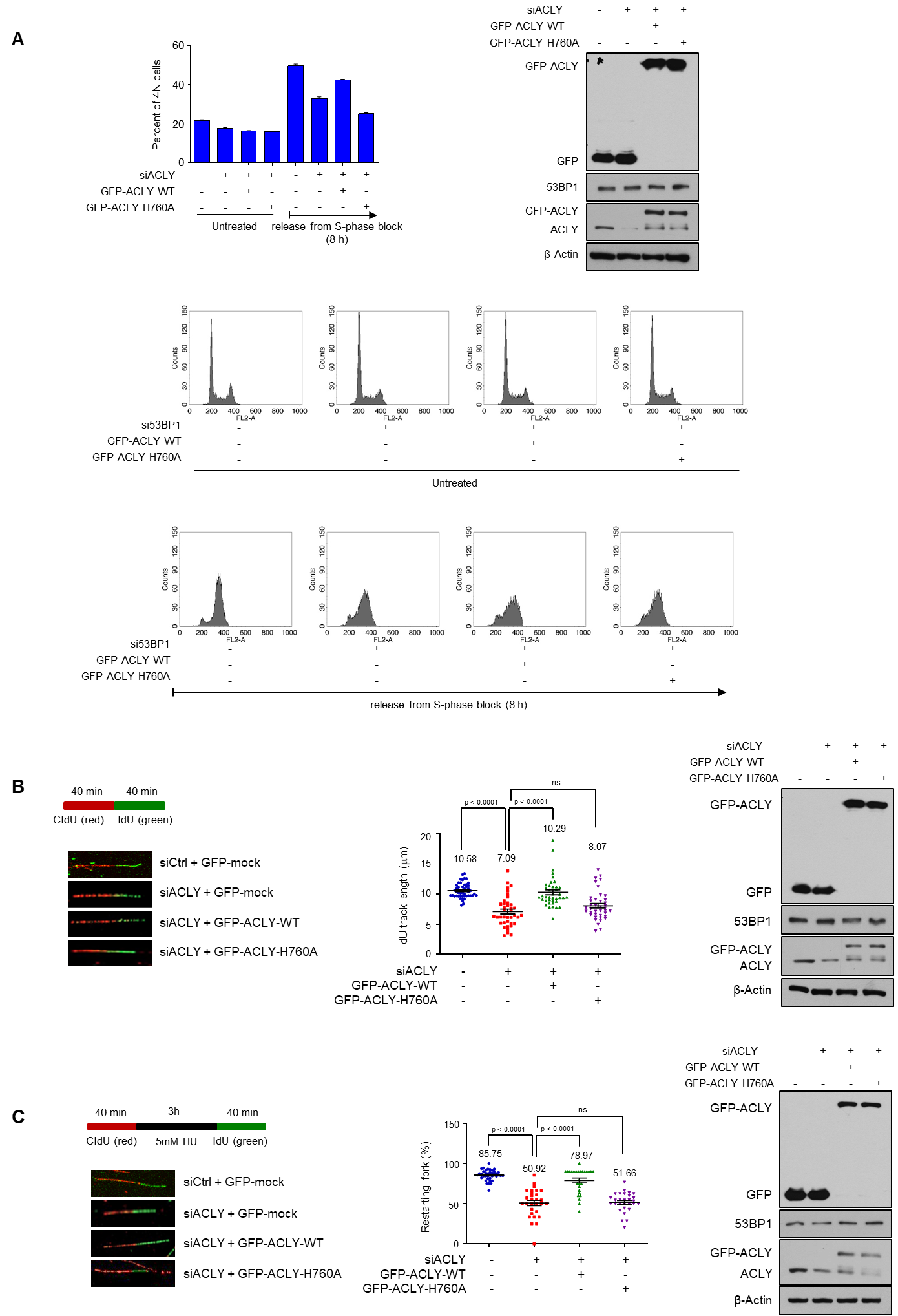


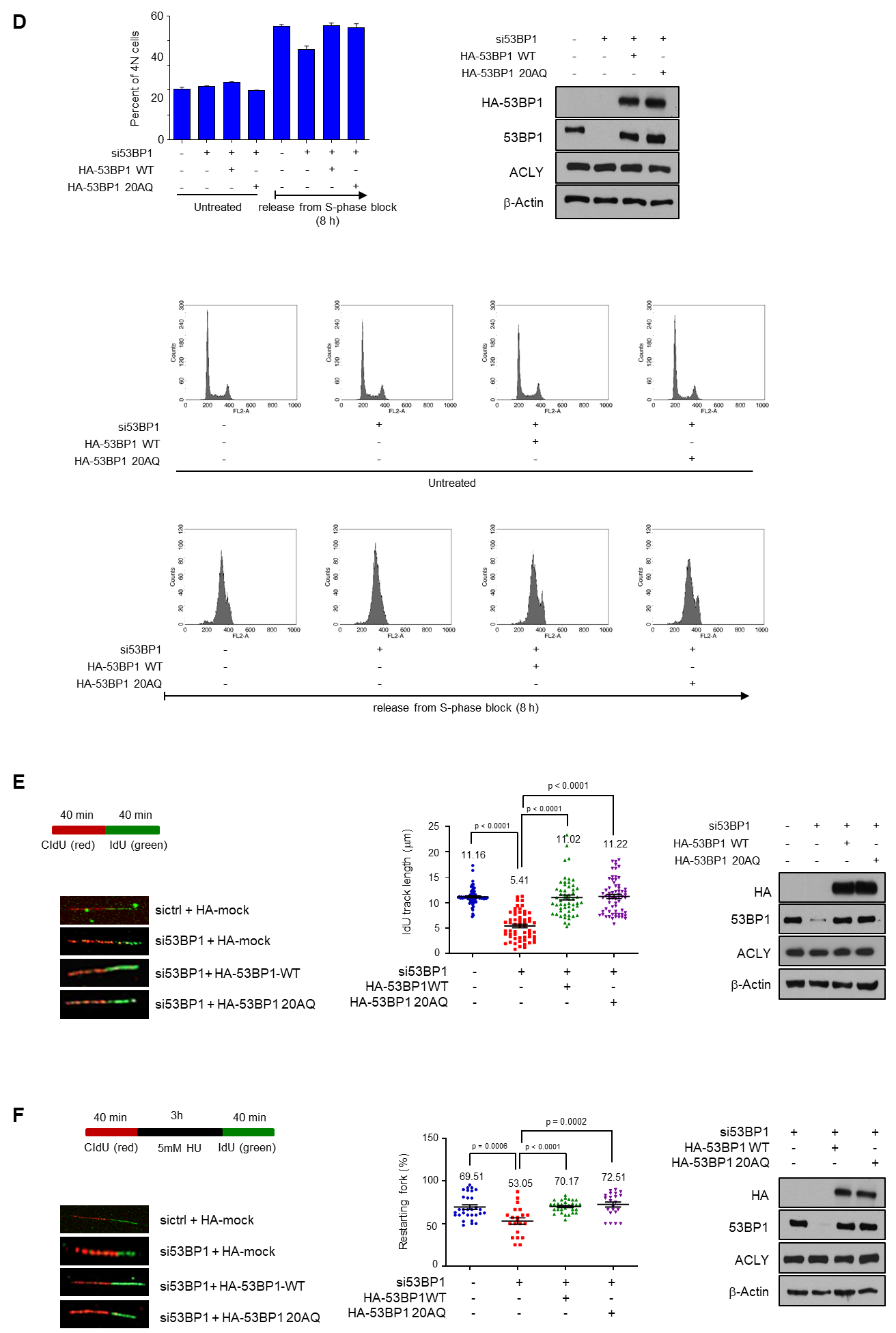


**Figure S12. Effect of phosphorylation of ACLY H760 and N-terminal SQ/TQ domain of 53BP1 on S-phase progression and recovery of stalled replication forks.**

(**A**) ACLY-depleted HeLa cells were transfected with control vector, GFP-ACLY-WT, or GFP-ACLY H760A and treated with aphidicolin to arrest cells in S-phase. Then cells were incubated with colcemid-containing medium to release from the arrest and to trap in M-phase. Cell cycle profiles were monitored by flow cytometry analysis of propidium iodide-stained cells to measure the nuclear DNA content. The results are shown as the mean ± SD (n = 3), ^**^*P* < 0.01.ns, not significant, Student’s *t*-test. (**B**) Replication elongation rates of control, ACLY-depleted, GFP-ACLY-WT-reconstituted/ACLY-depleted, or GFP-ACLY-H760A-reconstituted/ACLY-depleted HeLa cells were measured. Each cell was treated with CIdU (red) and IdU (green) for 40 min each and the IdU track length of CIdU-positive fibers was measured. Data represent mean ± SD (n = 3). *P* values between indicated samples were calculated using a Mann-Whitney test. **(C)** Replication fork restart of control, ACLY-depleted, GFP-ACLY-WT-reconstituted/ACLY-depleted, or GFP-ACLY-H760A-reconstituted/ACLY-depleted HeLa cells was measured. Pulse-labelling of cells with CIdU, HU (hydroxyurea), and IdU was done as shown in the schematic of experiment. DNA fibers were counted by contiguous IdU and CIdU tracks. Data represent mean ± SD (n = 3). *P* values between indicated samples were calculated using a Mann-Whitney test. (**D**) 53BP1-depleted HeLa cells were transfected with control vector, HA-53BP1-WT, or HA-53BP1 20AQ and treated with aphidicolin to arrest cells in S-phase. Then cells were incubated with colcemid-containing medium to release from the arrest and to trap in M-phase. Cell cycle profiles were monitored by flow cytometry analysis of propidium iodide-stained cells to measure the nuclear DNA content. The results are shown as the mean ± SD (n = 3), ^**^*P* < 0.01, Student’s *t*-test. (**E**) Replication elongation rates of control, 53BP1-depleted, HA-53BP1-WT-reconstituted/53BP1-depleted, or HA-53BP1-20AQ-reconstituted/53BP1-depleted HeLa cells were measured. Each cell was treated with CIdU (red) and IdU (green) for 40 min each and the IdU track length of CIdU-positive fibers was measured. Data represent mean ± SD (n = 3). *P* values between indicated samples were calculated using a Mann-Whitney test. **(F)** Replication fork restart of control, 53BP1-depleted, HA-53BP1-WT-reconstituted/53BP1-depleted, or HA-53BP1-20AQ-reconstituted/53BP1-depleted HeLa cells was measured. Pulse-labelling of cells with CIdU, HU (hydroxyurea), and IdU was done as shown in the schematic of experiment. DNA fibers were counted by contiguous IdU and CIdU tracks. Data represent mean ± SD (n = 3). *P* values between indicated samples were calculated using a Mann-Whitney test.

**
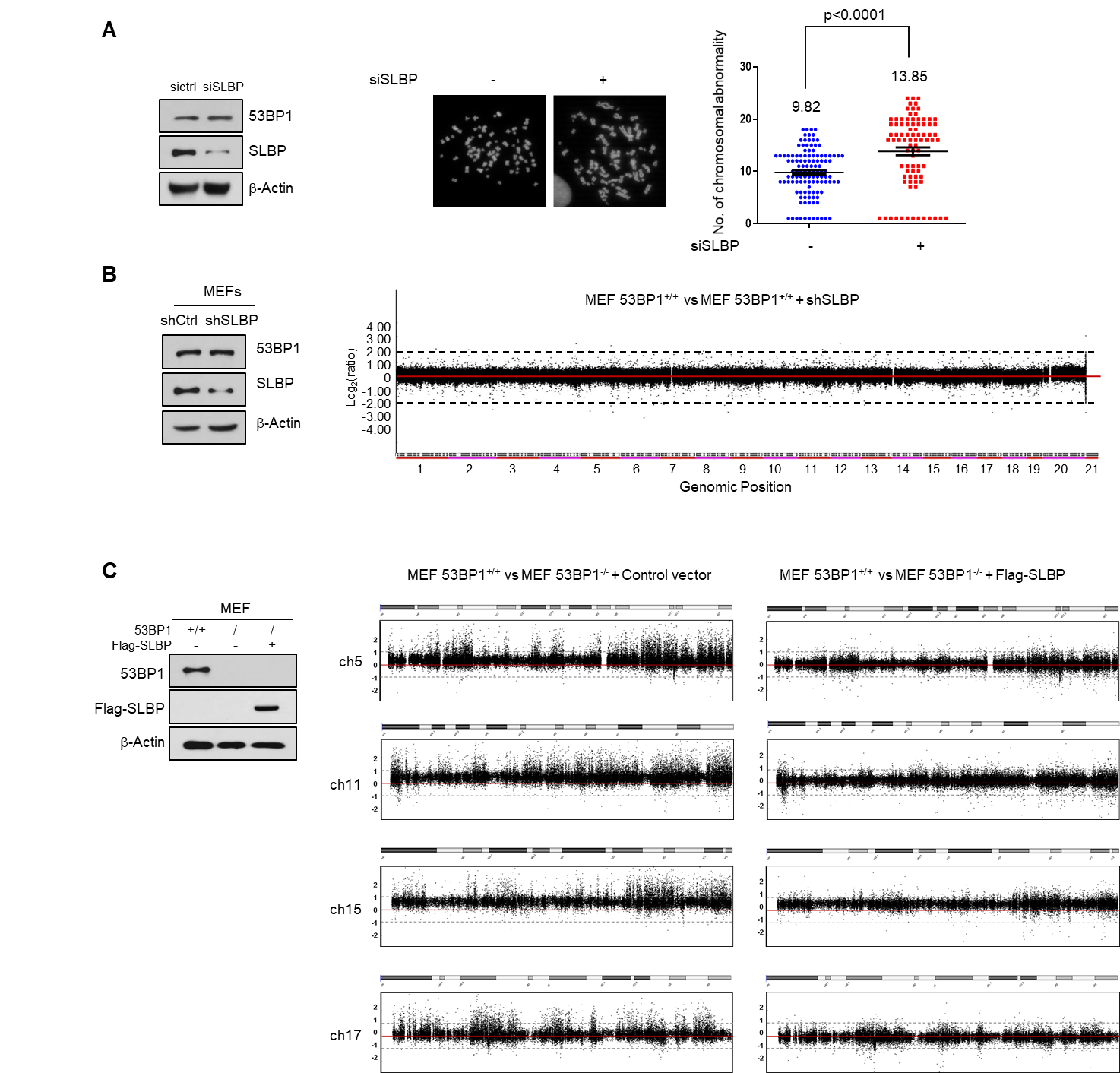
**

**Figure S13. The effect of SLBP downregulation on HU-induced chromosomal abnormalities and chromosome rearrangement is shown.**

(**A**) An analysis of chromosomal aberrations in control and SLBP-depleted MEFs is shown. Representative images of metaphase spreads in the presence of HU (2mM for 24 h) (left) and the plot of the numbers of chromosomal aberrations per metaphase (right) are shown. At least 50 metaphase cells were counted in each experiment. Data represent mean ± SD (n = 3). *P* values between indicated samples were calculated using a Mann-Whitney test. (**B**) Array CGH profiles of clones derived from control and SLBP-depleted MEFs are shown. (**C**) Array CGH profiles in clones derived from 53BP1^−/−^ MEFs (left), 53BP1^−/−^ MEF with SLBP-reconstitution (right) are shown. Normalized intensity ratios for each sample spotted according to cytogenic location, such that the upward deviation from the midline indicates the gain of copy numbers and the downward deviation from the midline indicates the loss of copy numbers.


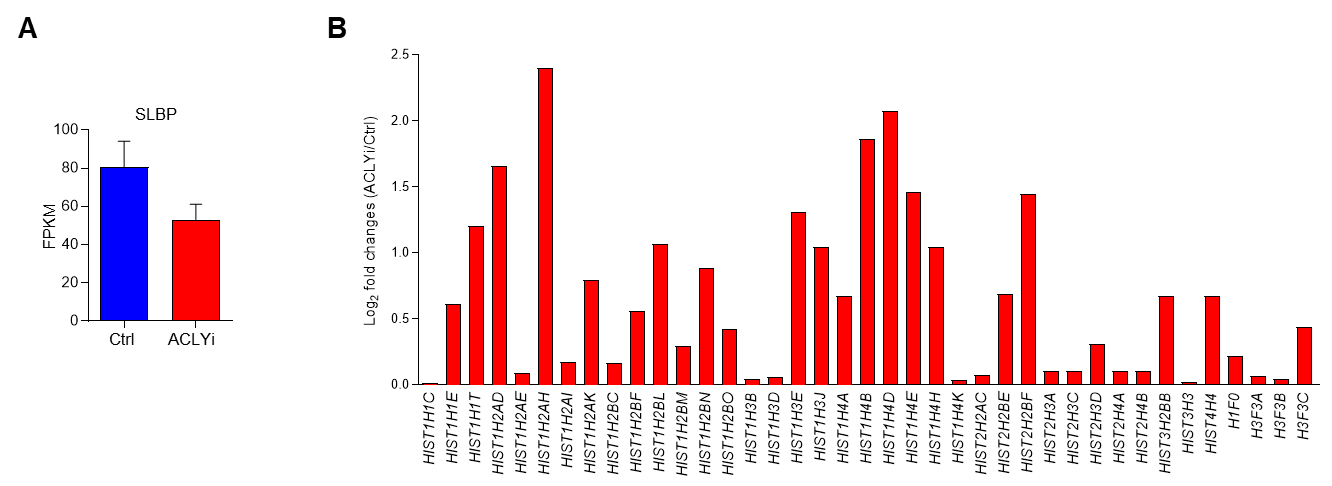


**Figure S14. The effect of an ACLY inhibition on SLBP expression and histone polyadenylation is shown.**

(**A**) C4-2 cells were treated with vehicle or ACLY inhibitor (MS-303141) and the levels of SLBP mRNA were analyzed by RNA-seq. Data represent mean ± SD (n = 3). (**B**) Fold changes (log_2_) of the polyadenylated replication-dependent histone gene families in the RNA-seq data from vehicle and ACLY inhibitor-treated C4-2 cells.

**Table S1. List of genes identified as differentially expressed between 53BP1^+/+^ and 53BP1^-/-^ MEFs.**

| **Higher expression in 53BP1^-/-^ MEFs** | | | |
| --- | --- | --- | --- |
| **Gene Name** | **logFC (53BP1^-/-^/53BP1^+/+^)** | **P-value** | **FDR** |
| *Peg3os* | -8.852002692 | 0.001808 | 0.071279 |
| *Gm5795* | -8.316889348 | 1.87E-09 | 1.76E-06 |
| *Kcna5* | -5.093331137 | 1.72E-08 | 1.19E-05 |
| *Chodl* | -4.093671232 | 1.58E-13 | 3.49E-10 |
| *Agtr1b* | -3.738979162 | 2.07E-05 | 0.003733 |
| *Eif3j2* | -3.367285735 | 6.41E-16 | 1.69E-12 |
| *Cntnap3* | -3.238983114 | 0.000578 | 0.035513 |
| *Lama3* | -3.197227355 | 0.000391 | 0.028172 |
| *Nxpe5* | -3.177692136 | 3.65E-06 | 0.001047 |
| *Nkain4* | -3.143699597 | 3.11E-09 | 2.74E-06 |
| *Trim13* | -3.00024572 | 5.52E-10 | 5.61E-07 |
| *Gm3488* | -2.972645614 | 3.06E-05 | 0.004819 |
| *Casq2* | -2.935925117 | 0.001648 | 0.067414 |
| *Dner* | -2.651802317 | 1.28E-10 | 1.54E-07 |
| *Rgs7bp* | -2.561983172 | 0.001028 | 0.049872 |
| *Cfh* | -2.532024929 | 5.14E-06 | 0.001305 |
| *Fam205a2* | -2.508335397 | 8.00E-05 | 0.009999 |
| *C1qtnf7* | -2.43959871 | 0.001224 | 0.054604 |
| *Tmtc1* | -2.391108675 | 0.000948 | 0.04833 |
| *Ang* | -2.387988655 | 1.15E-06 | 0.000411 |
| *Sorbs2* | -2.266066241 | 1.03E-07 | 5.27E-05 |
| *Clec12a* | -2.252927919 | 1.87E-05 | 0.003475 |
| *Ptpro* | -2.242194491 | 0.000369 | 0.02781 |
| *Pcdhgb8* | -2.240119455 | 1.45E-08 | 1.06E-05 |
| *Selp* | -2.227996073 | 5.76E-07 | 0.000217 |
| *Alox5* | -2.147317786 | 4.66E-05 | 0.006758 |
| *Gvin1* | -2.133716248 | 1.58E-05 | 0.003107 |
| *Vnn1* | -2.128451585 | 4.01E-06 | 0.001105 |
| *Parm1* | -2.125969637 | 3.90E-05 | 0.005925 |
| *Xkr4* | -2.11741321 | 0.002795 | 0.093947 |
| *Slc2a4* | -2.102389241 | 0.001293 | 0.057115 |
| *Cd72* | -2.090059811 | 1.64E-06 | 0.000554 |
| *Stap2* | -2.072886233 | 0.001343 | 0.058729 |
| *Pirb* | -2.07266399 | 0.000276 | 0.022869 |
| *Scn3b* | -2.072507656 | 0.000135 | 0.013679 |
| *Dhh* | -2.061778001 | 0.001992 | 0.07583 |
| *Tgtp1* | -2.059304089 | 0.000153 | 0.014782 |
| *Sp6* | -2.05622423 | 0.000267 | 0.022463 |
| *Snhg11* | -2.026986306 | 0.000425 | 0.029545 |
| *Dennd1c* | -2.003723528 | 0.002711 | 0.091506 |
| *Slc6a12* | -1.998130417 | 0.000507 | 0.032363 |
| *Trim30d* | -1.985486469 | 0.000975 | 0.048769 |
| *Il10ra* | -1.981631668 | 0.000381 | 0.027851 |
| *Rgs5* | -1.969125115 | 0.000314 | 0.025329 |
| *Dpyd* | -1.966026489 | 0.000171 | 0.01615 |
| *Apoe* | -1.965919079 | 1.59E-06 | 0.000553 |
| *Lyz1* | -1.956061497 | 3.55E-06 | 0.001047 |
| *Tifa* | -1.930226152 | 6.60E-06 | 0.001585 |
| *Btk* | -1.91806967 | 0.000649 | 0.038076 |
| *Myo1g* | -1.910892197 | 0.000105 | 0.011756 |
| *Enpp5* | -1.908762813 | 3.64E-09 | 3.00E-06 |
| *Gja3* | -1.884428389 | 0.000374 | 0.02781 |
| *Svop* | -1.878177538 | 0.000641 | 0.037824 |
| *Mctp1* | -1.87056494 | 0.000358 | 0.027516 |
| *Galr2* | -1.869031522 | 0.001021 | 0.049872 |
| *Cyth4* | -1.839909221 | 7.63E-05 | 0.009703 |
| *Galnt6* | -1.834197087 | 0.00161 | 0.066067 |
| *Hist1h4m* | -1.828650547 | 4.43E-05 | 0.006497 |
| *Adgre1* | -1.812950393 | 4.37E-08 | 2.41E-05 |
| *Tnfaip8l2* | -1.807904801 | 0.001105 | 0.051884 |
| *Cd14* | -1.798808932 | 4.36E-07 | 0.000173 |
| *Laptm5* | -1.792830395 | 2.75E-08 | 1.58E-05 |
| *Fcgr3* | -1.789926604 | 8.00E-06 | 0.001792 |
| *Dpep2* | -1.787050107 | 0.001126 | 0.052458 |
| *Fcer1g* | -1.786737091 | 1.78E-06 | 0.000588 |
| *Lyz2* | -1.780002831 | 4.46E-07 | 0.000173 |
| *Fcgr2b* | -1.763286567 | 0.00173 | 0.069469 |
| *Ccr5* | -1.759110584 | 0.001003 | 0.049719 |
| *Cybb* | -1.753093189 | 7.95E-06 | 0.001792 |
| *Gm3500* | -1.737380432 | 0.000836 | 0.044371 |
| *Kif1a* | -1.734446611 | 7.94E-12 | 1.17E-08 |
| *Serpinb6b* | -1.726133069 | 0.000111 | 0.011828 |
| *Mpeg1* | -1.710300208 | 1.04E-07 | 5.27E-05 |
| *Cd52* | -1.709835847 | 0.001172 | 0.053005 |
| *Efemp1* | -1.696744464 | 0.000408 | 0.028798 |
| *Exd1* | -1.693583198 | 0.000676 | 0.038492 |
| *Tyrobp* | -1.691167079 | 6.62E-05 | 0.008829 |
| *Aldh1a1* | -1.691002927 | 0.001481 | 0.062506 |
| *Snca* | -1.690507981 | 0.000853 | 0.044944 |
| *Itgb2* | -1.686516263 | 4.80E-05 | 0.006825 |
| *Lmo2* | -1.671241128 | 0.001149 | 0.052702 |
| *Gal3st2* | -1.659858039 | 0.001884 | 0.073 |
| *Fam150a* | -1.655720129 | 0.000393 | 0.028172 |
| *Myo1f* | -1.653531585 | 2.63E-05 | 0.004344 |
| *C1qb* | -1.65291484 | 2.59E-08 | 1.56E-05 |
| *Atp2a3* | -1.651476395 | 0.002862 | 0.095458 |
| *Pld4* | -1.647657969 | 0.000105 | 0.011756 |
| *Ctss* | -1.644958307 | 5.78E-06 | 0.001415 |
| *Fcrls* | -1.639559729 | 3.97E-06 | 0.001105 |
| *Ikzf1* | -1.636139363 | 0.001492 | 0.062734 |
| *Trem2* | -1.632683575 | 9.27E-05 | 0.011078 |
| *Pkia* | -1.631542802 | 3.49E-05 | 0.005422 |
| *Sh3tc1* | -1.630502767 | 0.001038 | 0.049872 |
| *Hist1h4h* | -1.62879722 | 0.00068 | 0.038537 |
| *Pgm5* | -1.627167625 | 0.002235 | 0.081125 |
| *Gm3558* | -1.626153016 | 0.000319 | 0.02536 |
| *Ccdc68* | -1.622789092 | 2.27E-07 | 1.00E-04 |
| *Cd300ld* | -1.620230784 | 0.00143 | 0.060917 |
| *P2ry6* | -1.617977826 | 0.000927 | 0.047814 |
| *Itgbl1* | -1.615855551 | 2.15E-08 | 1.42E-05 |
| *C1qc* | -1.609867465 | 1.36E-07 | 6.53E-05 |
| *Tlr7* | -1.602676079 | 0.000317 | 0.025356 |
| *Ncf1* | -1.601827453 | 7.64E-05 | 0.009703 |
| *Ptprc* | -1.600315961 | 9.41E-05 | 0.011098 |
| *Tlr8* | -1.589948019 | 0.001108 | 0.051884 |
| *Ms4a6d* | -1.581527509 | 0.000338 | 0.026404 |
| *Csf1r* | -1.564887496 | 2.55E-06 | 0.000801 |
| *Cd300c2* | -1.564188601 | 0.002333 | 0.083961 |
| *Snap91* | -1.562094086 | 0.002687 | 0.090999 |
| *Gpx3* | -1.560436975 | 4.20E-07 | 0.000173 |
| *C1qa* | -1.557890316 | 4.06E-07 | 0.000173 |
| *Efna3* | -1.550353144 | 0.002099 | 0.078096 |
| *Clec4d* | -1.542056893 | 0.00118 | 0.053005 |
| *Tmem151a* | -1.538972841 | 0.000213 | 0.019027 |
| *Aass* | -1.532016711 | 0.001038 | 0.049872 |
| *Maob* | -1.526924035 | 2.63E-05 | 0.004344 |
| *Mtss1* | -1.520910084 | 5.88E-05 | 0.00792 |
| *Akap6* | -1.511731238 | 0.000516 | 0.032428 |
| *Hist1h2bq* | -1.51027688 | 0.000587 | 0.035733 |
| *Hist1h2br* | -1.51027688 | 0.000587 | 0.035733 |
| *Lilrb4a* | -1.509788132 | 1.84E-05 | 0.003475 |
| *Sdsl* | -1.5049868 | 0.000973 | 0.048769 |
| *Csf2rb2* | -1.503572483 | 0.001868 | 0.072569 |
| *Ptpn6* | -1.471570326 | 0.000507 | 0.032363 |
| *Ms4a7* | -1.465232361 | 0.000742 | 0.040929 |
| *Dusp27* | -1.460480088 | 1.42E-05 | 0.002893 |
| *Pik3ap1* | -1.458467451 | 0.001153 | 0.052702 |
| *Hpgds* | -1.445270422 | 0.002243 | 0.081164 |
| *Myom1* | -1.440884754 | 1.05E-08 | 8.14E-06 |
| *Dock2* | -1.437921687 | 0.001709 | 0.068884 |
| *Ndrg2* | -1.436808189 | 0.001161 | 0.05275 |
| *Stab1* | -1.43616547 | 7.81E-06 | 0.001792 |
| *Hist1h2be* | -1.432744243 | 0.002323 | 0.083861 |
| *Lilr4b* | -1.413955721 | 0.000511 | 0.032402 |
| *Cd84* | -1.396153625 | 0.00052 | 0.032584 |
| *Pcsk6* | -1.393038376 | 4.71E-05 | 0.006758 |
| *Hist1h3d* | -1.392598372 | 0.002447 | 0.085985 |
| *Slc22a23* | -1.382823598 | 0.001578 | 0.064951 |
| *Arap3* | -1.381282898 | 0.002161 | 0.079536 |
| *Itgam* | -1.380218083 | 0.000741 | 0.040929 |
| *Spi1* | -1.376673822 | 0.002045 | 0.076742 |
| *Lcp1* | -1.368157498 | 4.50E-06 | 0.001213 |
| *Lpl* | -1.357090399 | 2.98E-05 | 0.004797 |
| *Smpdl3a* | -1.34415115 | 3.54E-05 | 0.005434 |
| *Hcls1* | -1.342751291 | 0.002454 | 0.085985 |
| *Tmem254a* | -1.340858943 | 0.001009 | 0.049719 |
| *Slc2a9* | -1.338476977 | 0.002179 | 0.079726 |
| *Nckap1l* | -1.318072166 | 0.000266 | 0.022463 |
| *Atp6v0c* | -1.311609883 | 7.70E-07 | 0.000283 |
| *Arhgap45* | -1.304105656 | 0.001383 | 0.059951 |
| *Ctsf* | -1.294495311 | 0.000106 | 0.011756 |
| *Myh11* | -1.284404837 | 1.50E-05 | 0.003012 |
| *Vav1* | -1.279209139 | 0.002524 | 0.087774 |
| *Selenop* | -1.269686627 | 0.000941 | 0.04833 |
| *Negr1* | -1.264857752 | 0.001936 | 0.074409 |
| *Cxcl16* | -1.261517103 | 0.001038 | 0.049872 |
| *Alox5ap* | -1.243575128 | 0.002067 | 0.077345 |
| *C3ar1* | -1.234390373 | 0.000673 | 0.038472 |
| *Slitrk5* | -1.233823195 | 0.000379 | 0.027851 |
| *Gca* | -1.230388917 | 0.001176 | 0.053005 |
| *Fas* | -1.229441094 | 0.000449 | 0.030564 |
| *Stmn2* | -1.228783484 | 0.001528 | 0.063862 |
| *Plek* | -1.226733703 | 0.000597 | 0.035834 |
| *Cd53* | -1.225293401 | 0.002411 | 0.085985 |
| *Itga11* | -1.217515693 | 0.000607 | 0.036141 |
| *Cpq* | -1.214625881 | 0.001453 | 0.061721 |
| *Hist2h2aa1* | -1.211116791 | 5.50E-05 | 0.007562 |
| *Fbln5* | -1.181778772 | 9.96E-05 | 0.011642 |
| *Aplp1* | -1.174297539 | 3.57E-06 | 0.001047 |
| *C5ar1* | -1.170535378 | 0.002445 | 0.085985 |
| *Adamts5* | -1.155162165 | 0.001574 | 0.064951 |
| *Cxcl12* | -1.140927721 | 0.002087 | 0.077884 |
| *Coro1a* | -1.136047712 | 0.002602 | 0.089498 |
| *Cx3cr1* | -1.133645295 | 0.002836 | 0.095078 |
| *Perp* | -1.115763021 | 0.001019 | 0.049872 |
| *Gpnmb* | -1.098211621 | 9.16E-05 | 0.011078 |
| *Adamts10* | -1.093735603 | 0.000555 | 0.034423 |
| *Eno3* | -1.092676534 | 0.000725 | 0.040414 |
| *Itga8* | -1.092181985 | 1.32E-05 | 0.00277 |
| *Tcn2* | -1.081381662 | 1.20E-05 | 0.002604 |
| *Col8a1* | -1.07934436 | 0.000119 | 0.012349 |
| *Acyp2* | -1.076712921 | 0.002022 | 0.0761 |
| *Rflnb* | -1.073169295 | 2.53E-05 | 0.004288 |
| *Pla2r1* | -1.070292281 | 0.000607 | 0.036141 |
| *Tinagl1* | -1.067409585 | 0.00011 | 0.011828 |
| *Atp9a* | -1.057887927 | 0.000243 | 0.020981 |
| *Ppip5k1* | -1.038632772 | 0.000395 | 0.028172 |
| *Klhl13* | -1.035984118 | 0.000958 | 0.048691 |
| *Alpk1* | -1.033208048 | 0.000388 | 0.028139 |
| *Cd68* | -1.023257298 | 0.001397 | 0.060283 |
| *Fam160a1* | -1.02179011 | 0.002622 | 0.089965 |
| *Hoxb3* | -1.009909209 | 0.000476 | 0.031595 |

| **Higher expression in WT** | | | |
| --- | --- | --- | --- |
| **Gene Name** | **logFC (53BP1^-/-^/53BP1^+/+^)** | **P-value** | **FDR** |
| *C330027C09Rik* | 1.003006795 | 0.000286 | 0.023486 |
| *Anln* | 1.007771192 | 0.000107 | 0.011785 |
| *Lig1* | 1.010490497 | 0.000136 | 0.013679 |
| *Igf2os* | 1.010497859 | 0.002022 | 0.0761 |
| *Aurka* | 1.011259687 | 0.000969 | 0.048769 |
| *Cdca8* | 1.01461218 | 0.000503 | 0.032363 |
| *Slc1a5* | 1.01636806 | 0.002157 | 0.079536 |
| *Mcm7* | 1.019231583 | 0.000145 | 0.014287 |
| *Kif22* | 1.024062713 | 0.000471 | 0.031471 |
| *Nek2* | 1.025053109 | 0.000672 | 0.038472 |
| *Trip13* | 1.026280207 | 0.00288 | 0.095826 |
| *Shcbp1* | 1.029586838 | 0.001496 | 0.062734 |
| *Nasp* | 1.030101398 | 0.000593 | 0.035743 |
| *S1pr3* | 1.035065489 | 0.00014 | 0.01388 |
| *Dnmt1* | 1.037577656 | 0.000127 | 0.013094 |
| *Mcm4* | 1.042946779 | 0.000614 | 0.036364 |
| *Pbk* | 1.044646946 | 0.001384 | 0.059951 |
| *Ccnb2* | 1.045306872 | 0.001143 | 0.052592 |
| *Ncapg* | 1.048116139 | 0.00087 | 0.04523 |
| *Iqgap3* | 1.053854374 | 0.000239 | 0.020764 |
| *Cep55* | 1.057781996 | 0.001361 | 0.059357 |
| *Ndc80* | 1.058107374 | 0.00077 | 0.042233 |
| *Kif20b* | 1.058650553 | 0.000791 | 0.04299 |
| *Hmgn2* | 1.060214784 | 0.001063 | 0.050331 |
| *Igfbp6* | 1.060581726 | 0.002525 | 0.087774 |
| *Foxm1* | 1.062802512 | 3.06E-05 | 0.004819 |
| *Plk4* | 1.070683925 | 0.000722 | 0.040414 |
| *Bub1* | 1.07297417 | 0.000744 | 0.040929 |
| *Ube2c* | 1.074221557 | 0.000192 | 0.017658 |
| *Prc1* | 1.083090241 | 0.000169 | 0.016039 |
| *Kif2c* | 1.088274714 | 0.000472 | 0.031471 |
| *Lrrc17* | 1.092439719 | 0.001423 | 0.060854 |
| *Rad51* | 1.095716059 | 0.001231 | 0.054737 |
| *Cdt1* | 1.097004105 | 0.001046 | 0.049937 |
| *Fanca* | 1.099449625 | 0.000302 | 0.024624 |
| *Mis18bp1* | 1.102869483 | 0.002164 | 0.079536 |
| *Ect2* | 1.107331898 | 0.000259 | 0.022189 |
| *Mcm3* | 1.1121676 | 0.000363 | 0.027525 |
| *Cenpa* | 1.113676182 | 0.000444 | 0.030522 |
| *Spag5* | 1.118729859 | 0.000113 | 0.011828 |
| *Racgap1* | 1.122923597 | 7.21E-06 | 0.001701 |
| *Mcm2* | 1.12322461 | 0.000102 | 0.011756 |
| *Cenpe* | 1.124816079 | 0.002438 | 0.085985 |
| *Recql4* | 1.133066698 | 0.00292 | 0.096923 |
| *Nptxr* | 1.134292758 | 0.000457 | 0.030971 |
| *Ckap2l* | 1.140596574 | 7.60E-05 | 0.009703 |
| *Bnc2* | 1.146859804 | 0.000502 | 0.032363 |
| *Pif1* | 1.149544103 | 0.002168 | 0.079536 |
| *Hjurp* | 1.150692113 | 1.22E-05 | 0.002604 |
| *Ncapd2* | 1.152014462 | 4.87E-06 | 0.001283 |
| *Ccnb1* | 1.152673769 | 6.87E-05 | 0.009028 |
| *Kif18b* | 1.152771813 | 0.000157 | 0.015062 |
| *Kif23* | 1.155777259 | 2.85E-05 | 0.004643 |
| *E2f8* | 1.162432688 | 0.000806 | 0.043618 |
| *Bub1b* | 1.165560697 | 4.31E-05 | 0.006398 |
| *Melk* | 1.174379989 | 0.000467 | 0.031471 |
| *Tk1* | 1.177910803 | 8.39E-05 | 0.010261 |
| *Hmgb3* | 1.178439114 | 0.000211 | 0.018996 |
| *Chaf1b* | 1.186306209 | 0.000207 | 0.018891 |
| *Dnph1* | 1.188153184 | 0.000668 | 0.038472 |
| *Aurkb* | 1.19124882 | 5.69E-05 | 0.007751 |
| *Cenpf* | 1.19308052 | 0.002008 | 0.0761 |
| *Cdca2* | 1.19558904 | 0.000105 | 0.011756 |
| *Rad54l* | 1.19561112 | 0.00168 | 0.068487 |
| *Uhrf1* | 1.205709405 | 2.11E-05 | 0.003733 |
| *Cdc25c* | 1.210679088 | 0.001953 | 0.074571 |
| *Steap1* | 1.211275886 | 0.001162 | 0.05275 |
| *Timeless* | 1.212155836 | 4.98E-05 | 0.006952 |
| *Ada* | 1.212959272 | 0.00014 | 0.01388 |
| *Pclaf* | 1.213376948 | 8.39E-05 | 0.010261 |
| *Prr11* | 1.213768517 | 0.000109 | 0.011828 |
| *Birc5* | 1.215417384 | 1.68E-05 | 0.003247 |
| *Pola1* | 1.217361151 | 0.000861 | 0.044972 |
| *Cit* | 1.220365498 | 0.000209 | 0.018923 |
| *Dio3* | 1.221557339 | 0.001128 | 0.052458 |
| *Prim1* | 1.222063935 | 0.002649 | 0.090439 |
| *Kif4* | 1.222979779 | 9.31E-05 | 0.011078 |
| *Top2a* | 1.224749433 | 0.000238 | 0.020764 |
| *Gfra1* | 1.226680448 | 0.00015 | 0.014691 |
| *Plk1* | 1.227236444 | 4.95E-06 | 0.001283 |
| *Lmnb1* | 1.22848185 | 2.08E-06 | 0.000671 |
| *Depdc1a* | 1.231539973 | 0.001535 | 0.063973 |
| *Sgol1* | 1.236794437 | 0.000496 | 0.032363 |
| *Kntc1* | 1.237561918 | 0.001826 | 0.071782 |
| *Espl1* | 1.239002194 | 1.70E-05 | 0.003247 |
| *Kif11* | 1.239407198 | 0.000112 | 0.011828 |
| *Dut* | 1.243260622 | 0.000191 | 0.017658 |
| *Dtl* | 1.244455045 | 0.002476 | 0.086523 |
| *Cdca7l* | 1.265441123 | 0.000831 | 0.044371 |
| *Hells* | 1.270466468 | 0.000968 | 0.048769 |
| *Ercc6l* | 1.283118158 | 0.000488 | 0.032066 |
| *BC030867* | 1.304050625 | 0.001077 | 0.050813 |
| *Mcm5* | 1.310389302 | 1.89E-05 | 0.003475 |
| *Pole* | 1.313149741 | 0.0004 | 0.028404 |
| *Cdc45* | 1.317840342 | 0.000593 | 0.035743 |
| *Ttk* | 1.318292849 | 0.000104 | 0.011756 |
| *Hmgb2* | 1.319187322 | 3.16E-06 | 0.000971 |
| *Neto2* | 1.323562306 | 4.23E-05 | 0.006354 |
| *Ccna2* | 1.323855891 | 5.45E-06 | 0.001359 |
| *Mcm6* | 1.324946743 | 1.04E-05 | 0.002284 |
| *Stc2* | 1.328009001 | 0.001334 | 0.058544 |
| *Cdca7* | 1.335125343 | 0.000222 | 0.01971 |
| *Pole2* | 1.342146944 | 0.001206 | 0.054009 |
| *Mki67* | 1.35902481 | 0.001807 | 0.071279 |
| *6430706D22Rik* | 1.366732337 | 1.38E-07 | 6.53E-05 |
| *Sbsn* | 1.374776698 | 0.000478 | 0.031595 |
| *Fbxo5* | 1.381860529 | 0.00056 | 0.034586 |
| *Kif15* | 1.382157665 | 8.02E-05 | 0.009999 |
| *Nt5e* | 1.388832363 | 1.39E-05 | 0.002878 |
| *Cdc7* | 1.395733132 | 0.000113 | 0.011828 |
| *Il1rl2* | 1.397675169 | 0.000184 | 0.017255 |
| *E2f7* | 1.398246981 | 0.000338 | 0.026404 |
| *Clspn* | 1.399297082 | 0.001141 | 0.052592 |
| *Wdhd1* | 1.406301894 | 0.000783 | 0.042716 |
| *Jmjd7* | 1.412446726 | 0.00171 | 0.068884 |
| *Kif14* | 1.424491676 | 2.39E-05 | 0.004103 |
| *Mybl2* | 1.438274103 | 5.00E-05 | 0.006952 |
| *Trp53bp1* | 1.443978625 | 2.46E-08 | 1.55E-05 |
| *Npnt* | 1.468922108 | 0.000274 | 0.022869 |
| *Ticrr* | 1.478092083 | 0.000513 | 0.032402 |
| *Tnfsf11* | 1.494591019 | 0.001853 | 0.072204 |
| *Ska1* | 1.500935152 | 0.000697 | 0.039337 |
| *Galnt14* | 1.505705691 | 0.002635 | 0.090164 |
| *Slco2a1* | 1.516001754 | 0.000382 | 0.027851 |
| *Bcl11b* | 1.525634513 | 0.001847 | 0.072204 |
| *Cdc6* | 1.546402566 | 0.000277 | 0.022869 |
| *Exo1* | 1.55382658 | 0.000548 | 0.034125 |
| *Lmo1* | 1.560655901 | 0.001903 | 0.073489 |
| *Hoxc10* | 1.567737159 | 0.000234 | 0.020651 |
| *Gm5801* | 1.68202286 | 0.000305 | 0.024748 |
| *Lrrc15* | 1.711600541 | 0.000498 | 0.032363 |
| *Col18a1* | 1.743334716 | 6.08E-12 | 1.00E-08 |
| *Tmem132e* | 1.755225513 | 0.001401 | 0.060283 |
| *Crispld2* | 1.757525151 | 0.000714 | 0.040122 |
| *Col5a3* | 1.788473974 | 1.91E-07 | 8.71E-05 |
| *Egr2* | 1.941031139 | 0.002452 | 0.085985 |
| *Itgb7* | 1.965520007 | 0.001692 | 0.068724 |
| *Tnn* | 2.003665897 | 0.002678 | 0.090935 |
| *Col6a3* | 2.049123346 | 2.60E-11 | 3.44E-08 |
| *Adh7* | 2.067105647 | 0.001047 | 0.049937 |
| *Il1rl1* | 2.089385431 | 0.000374 | 0.02781 |
| *Spock3* | 2.192859283 | 2.18E-05 | 0.003786 |
| *Barx1* | 2.208959963 | 0.002383 | 0.085297 |
| *Col15a1* | 2.336185938 | 2.12E-05 | 0.003733 |
| *Ahcy* | 2.445405501 | 1.65E-16 | 5.46E-13 |
| *Car6* | 2.494336633 | 0.000192 | 0.017658 |
| *Tmem100* | 2.762941134 | 0.001032 | 0.049872 |
| *Gm1045* | 2.768336094 | 0.001001 | 0.049719 |
| *Pkp1* | 3.002963837 | 0.000447 | 0.030564 |
| *Gm10653* | 3.062787519 | 1.44E-10 | 1.58E-07 |
| *Otud7a* | 3.226347787 | 0.000413 | 0.028972 |
| *U90926* | 3.801737706 | 0.00067 | 0.038472 |
| *Gm8801* | 6.153799608 | 3.38E-13 | 6.38E-10 |
| *Eif3j1* | 6.981539048 | 1.04E-52 | 6.84E-49 |
| *Hmga1-rs1* | 7.749794931 | 2.83E-42 | 1.25E-38 |
| *Atp6v0c-ps2* | 7.792177928 | 7.24E-72 | 9.56E-68 |

**Table S2. List of polyadenylated histone genes expression in 53BP1^+/+^ and 53BP1^-/-^ MEFs.**

| **Replication-dependent histones** | | | | |
| --- | --- | --- | --- | --- |
| **Gene Name** | **Transcript Name** | **TPM**  **(53BP1^-/-^)** | **TPM**  **(53BP1^+/+^)** | **log_2_**  **(53BP1^-/-^/53BP1^+/+^)** |
| *Hist1h1a* | NM_030609 | 0.543834 | 0.388445 | 0.485455 |
| *Hist1h1b* | NM_020034 | 1.518455 | 0.888212 | 0.773628 |
| *Hist1h1c* | NM_015786 | 167.7735 | 101.037 | 0.731631 |
| *Hist1h1d* | NM_145713 | 5.902045 | 2.718205 | 1.118561 |
| *Hist1h1e* | NM_015787 | 4.05784 | 1.448545 | 1.486107 |
| *Hist1h1t* | NM_010377 | 0.13812 | 0.112491 | 0.296113 |
| *Hist1h2ab* | NM_175660 | 24.9295 | 15.11067 | 0.722287 |
| *Hist1h2ac* | NM_178189 | 4.01185 | 2.979425 | 0.429234 |
| *Hist1h2ad* | NM_178188 | 12.73675 | 5.98268 | 1.090133 |
| *Hist1h2ae* | NM_178187 | 3.188235 | 1.96669 | 0.696988 |
| *Hist1h2af* | NM_175661 | 1.866656 | 1.88888 | -0.01707 |
| *Hist1h2ag* | NM_178186 | 0.938891 | 1.125762 | -0.26187 |
| *Hist1h2ah* | NM_175659 | 0.49416 | 0.479428 | 0.043666 |
| *Hist1h2ai* | NM_178182 | 4.37902 | 1.30848 | 1.742716 |
| *Hist1h2ak* | NM_178183 | 0.27157 | 0.143472 | 0.920555 |
| *Hist1h2al* | NM_003511 | 0.311323 | 0.292534 | 0.089808 |
| *Hist1h2an* | NM_178184 | 0.132731 | 0.3164 | -1.25324 |
| *Hist1h2ao* | NM_001177544 | 2.12997 | 1.87438 | 0.18442 |
| *Hist1h2ap* | NM_178185 | 1.145222 | 2.655354 | -1.21328 |
| *Hist1h2bb* | NM_175664 | 2.999365 | 2.44318 | 0.295897 |
| *Hist1h2bc* | NM_023422 | 322.0934 | 200.1858 | 0.68614 |
| *Hist1h2be* | NM_178194 | 11.36811 | 3.378571 | 1.750507 |
| *Hist1h2bf* | NM_178195 | 1.164915 | 1.40733 | -0.27274 |
| *Hist1h2bg* | NM_178196 | 12.43211 | 8.739905 | 0.508381 |
| *Hist1h2bh* | NM_178197 | 1.78065 | 1.329265 | 0.421775 |
| *Hist1h2bj* | NM_178198 | 5.567255 | 3.70156 | 0.588833 |
| *Hist1h2bk* | NM_175665 | 1.064319 | 0.902834 | 0.237397 |
| *Hist1h2bl* | NM_178199 | 2.34156 | 0.896718 | 1.384744 |
| *Hist1h2bm* | NM_178200 | 0.284051 | 0.19209 | 0.564368 |
| *Hist1h2bn* | NM_178201 | 2.62851 | 1.601642 | 0.714694 |
| *Hist1h2bp* | NM_178202 | 1.077135 | 0.734201 | 0.552952 |
| *Hist1h2bq* | NM_001313880 | 45.66235 | 11.76153 | 1.956929 |
| *Hist1h2br* | NM_001313878 | 45.66235 | 11.76153 | 1.956929 |
| *Hist1h3a* | NM_013550 | 0.133401 | 0.720196 | -2.43263 |
| *Hist1h3b* | NM_178203 | 28.8204 | 18.87415 | 0.610679 |
| *Hist1h3c* | NM_175653 | 7.008495 | 2.822955 | 1.311899 |
| *Hist1h3d* | NM_178204 | 26.774 | 10.14475 | 1.400099 |
| *Hist1h3e* | NM_178205 | 1.227265 | 1.901555 | -0.63173 |
| *Hist1h3f* | NM_013548 | 0.947633 | 0.331042 | 1.517313 |
| *Hist1h3g* | NM_145073 | 2.01997 | 1.30774 | 0.627258 |
| *Hist1h3i* | NM_178207 | 3.29352 | 2.93658 | 0.165493 |
| *Hist1h4a* | NM_178192 | 4.01508 | 1.305106 | 1.621262 |
| *Hist1h4b* | NM_178193 | 0.493784 | 0.769943 | -0.64087 |
| *Hist1h4c* | NM_178208 | 2.101945 | 0.290096 | 2.857125 |
| *Hist1h4d* | NM_175654 | 1.968575 | 1.118794 | 0.815208 |
| *Hist1h4h* | NM_153173 | 0.172778 | 0.210363 | -0.28396 |
| *Hist1h4i* | NM_175656 | 26.75615 | 8.48232 | 1.65734 |
| *Hist1h4j* | NM_178210 | 36.9962 | 24.0095 | 0.623772 |
| *Hist1h4k* | NM_178211 | 0.208559 | 0.886057 | -2.08695 |
| *Hist1h4m* | NM_001195421 | 1.157012 | 1.35821 | -0.2313 |
| *Hist2h2aa1* | NM_013549 | 279.613 | 120.3123 | 1.216647 |
| *Hist2h2aa2* | NM_178212 | 19.77421 | 19.16707 | 0.04499 |
| *Hist2h2ab* | NM_178213 | 0.23852 | 0.118868 | 1.004747 |
| *Hist2h2ac* | NM_175662 | 0.461061 | 0.408395 | 0.174993 |
| *Hist2h2bb* | NM_175666 | 0.099513 | 0.101564 | -0.02944 |
| *Hist2h2be* | NM_178214 | 1.382525 | 1.4765 | -0.09488 |
| *Hist2h3c1* | NM_178216 | 16.30706 | 4.16416 | 1.969399 |
| *Hist2h3c2* | NM_054045 | 88.6041 | 37.33401 | 1.246883 |
| *Hist2h4* | NM_033596 | 1.43141 | 0.384853 | 1.895059 |
| *Hist3h2a* | NM_178218 | 88.2646 | 63.6892 | 0.470786 |
| *Hist4h4* | NM_175652 | 0.352885 | 0.180007 | 0.971149 |

| **Replication-independent histones** | | | | |
| --- | --- | --- | --- | --- |
| **Gene Name** | **Transcript Name** | **TPM**  **(53BP1^-/-^)** | **TPM**  **(53BP1^+/+^)** | **log_2_**  **(53BP1^-/-^/53BP1^+/+^)** |
| *H1f0* | NM_008197 | 92.70955 | 103.2601 | -0.15549 |
| *H1fx* | NM_198622 | 10.24169 | 20.8955 | -1.02874 |
| *H2afj* | NM_177688 | 66.81125 | 56.41185 | 0.244093 |
| *H2afv* | NM_029938 | 22.57729 | 27.44444 | -0.28164 |
| *H2afx* | NM_010436 | 52.5841 | 73.93535 | -0.49164 |
| *H2afy* | NM_012015 | 91.18131 | 108.3722 | -0.24918 |
| *H2afy2* | NM_207000 | 33.73145 | 54.0497 | -0.68019 |
| *H2afy3* | NR_003523 | 0.631564 | 0.819407 | -0.37565 |
| *H2afz* | NM_016750 | 244.5887 | 425.1933 | -0.79776 |
| *H3f3a* | NM_008210 | 557.417 | 596.1125 | -0.09683 |
| *H3f3b* | NM_008211 | 261.439 | 379.0445 | -0.53589 |

**Table S3. List of histone metabolism-related genes expression in 53BP1^+/+^ and 53BP^-/-^ MEFs.**

| **Gene Name** | **TPM (53BP1^-/-^)** | **TPM (53BP1^+/+^)** | **log_2_ (53BP1^-/-^/53BP1^+/+^)** |
| --- | --- | --- | --- |
| *Slbp* | 31.17816 | 52.48754 | -0.75144 |
| *Casp8ap2 (Flash)* | 7.294348 | 11.22543 | -0.62192 |
| *Cstf2 (Cstf64)* | 16.17991 | 23.17303 | -0.51824 |
| *Srrt* | 67.24133 | 88.36353 | -0.3941 |
| *Cpsf2 (Cpsf100)* | 27.46 | 35.8418 | -0.38431 |
| *Cpsf6 (CFim68)* | 22.26078 | 27.80787 | -0.32099 |
| *Nelf* | 23.63925 | 28.77065 | -0.28341 |
| *Lsm11* | 4.496315 | 5.364885 | -0.2548 |
| *Npat* | 10.94303 | 12.54765 | -0.1974 |
| *Zbtb17 (Zfp100)* | 15.0235 | 16.9634 | -0.1752 |
| *Cpsf3 (Cpsf73)* | 44.3998 | 46.63195 | -0.07077 |
| *Cdk9* | 45.9424 | 47.81465 | -0.05763 |
| *Lsm10* | 41.85522 | 39.51472 | 0.083017 |
| *Sympk* | 90.071 | 82.6427 | 0.124175 |
| *Mif4gd (Slip1)* | 23.44467 | 20.55029 | 0.190101 |

**Table S4. List of polyadenylated histone genes expression in Ctrl and ACLYi.**

| **Replication-dependent Histones** | | | | |
| --- | --- | --- | --- | --- |
| **Gene Name** | **Transcript Name** | **TPM (ACLYi)** | **TPM (Ctrl)** | **log_2_ (ACLYi/Ctrl)** |
| *HIST1H1C* | NM_005319 | 339.771 | 336.2905 | 0.014854697 |
| *HIST1H1E* | NM_005321 | 3.41111 | 2.23538 | 0.609721178 |
| *HIST1H1T* | NM_005323 | 1.2188875 | 0.5289295 | 1.204417629 |
| *HIST1H2AD* | NM_021065 | 2.512185 | 0.796095 | 1.657930203 |
| *HIST1H2AE* | NM_021052 | 7.92326 | 7.436365 | 0.091496561 |
| *HIST1H2AH* | NM_080596 | 1.270636 | 0.240771 | 2.399817259 |
| *HIST1H2AI* | NM_003509 | 12.99915 | 11.5468 | 0.170924202 |
| *HIST1H2AK* | NM_003510 | 18.3712 | 10.59619 | 0.793900248 |
| *HIST1H2BC* | NM_003526 | 46.7532 | 41.7114 | 0.164623377 |
| *HIST1H2BF* | NM_003522 | 12.42735 | 8.44516 | 0.557322028 |
| *HIST1H2BL* | NM_003519 | 18.79075 | 8.98045 | 1.065163007 |
| *HIST1H2BM* | NM_003521 | 0.705915 | 0.57661 | 0.29189862 |
| *HIST1H2BN* | NM_003520 | 17.5308 | 9.509 | 0.882526298 |
| *HIST1H2BO* | NM_003527 | 10.81325 | 8.08089 | 0.420214101 |
| *HIST1H3B* | NM_003537 | 3.93757 | 3.81559 | 0.045399415 |
| *HIST1H3D* | NM_001376937 | 5.13676 | 4.93947 | 0.056502419 |
| *HIST1H3E* | NM_003532 | 19.59895 | 7.883545 | 1.313859947 |
| *HIST1H3J* | NM_003535 | 3.582275 | 1.73915 | 1.042493724 |
| *HIST1H4A* | NM_003538 | 0.62691 | 0.3924705 | 0.675674125 |
| *HIST1H4B* | NM_003544 | 6.399575 | 1.760865 | 1.861691791 |
| *HIST1H4D* | NM_003539 | 3.4935 | 0.8280265 | 2.076924295 |
| *HIST1H4E* | NM_003545 | 20.57785 | 7.45225 | 1.465344277 |
| *HIST1H4H* | NM_003543 | 107.8661 | 52.22725 | 1.046366882 |
| *HIST1H4K* | NM_003541 | 4.136355 | 4.02794 | 0.038317816 |
| *HIST2H2AC* | NM_003517 | 4.48191 | 4.24899 | 0.076993731 |
| *HIST2H2BE* | NM_003528 | 120.34 | 74.6487 | 0.688927221 |
| *HIST2H2BF* | NM_001024599 | 23.11152 | 8.47536 | 1.447265592 |
| *HIST2H3A* | NM_001005464 | 3.755825 | 3.4904 | 0.105737464 |
| *HIST2H3C* | NM_021059 | 3.755825 | 3.4904 | 0.105737464 |
| *HIST2H3D* | NM_001123375 | 4.346525 | 3.502605 | 0.311434143 |
| *HIST2H4A* | NM_003548 | 73.15305 | 68.0201 | 0.104956889 |
| *HIST2H4B* | NM_001034077 | 73.15305 | 68.0201 | 0.104956889 |
| *HIST3H2BB* | NM_175055 | 6.102025 | 3.830745 | 0.671663097 |
| *HIST3H3* | NM_003493 | 0.5014475 | 0.4932925 | 0.02365531 |
| *HIST4H4* | NM_175054 | 8.31134 | 5.209175 | 0.67402619 |
| *H1F0* | NM_005318 | 130.4925 | 112.1685 | 0.218299306 |
| *H3F3A* | NM_002107 | 624.575 | 597.521 | 0.063885405 |
| *H3F3B* | NM_005324 | 333.4165 | 323.2775 | 0.044552401 |
| *H3F3C* | NM_001013699 | 0.808776 | 0.598125 | 0.435293167 |

**Table S5. List of primer sequences used for RT-PCR, ChIP and cloning.**

| **Gene** | | **Forward primer sequence** | **Reverse primer sequence** | **Used** |
| --- | --- | --- | --- | --- |
| *Slbp*(mouse) | | TCTGGAAGGTGGCTTTGCAT | GTGCCAGCATACACATCAAAGTT | RT-PCR |
| *Srrt*(mouse) | | CCTCCCATCTTGGGCTATGG | CACTATGGCCCTTGGGTCTC | RT-PCR |
| *Casp8ap2*(mouse) | | GGTAAGTTCCTGAGGGCCTG | CTGTAGACCCGGCGATGTTC | RT-PCR |
| *Cstf2*(mouse) | | TCAATGGCGCACCTCCTATG | CTGCTCTGGGGTCTTGCATT | RT-PCR |
| *Cpsf2*(mouse) | | CAGGGTGTGAGACACCAGTC | ACTGGCCTTCAGCCAAGTTT | RT-PCR |
| *Lsm11*(mouse) | | GCAATGTGCTTACGCGGATG | TGAAGGTGCGGATGTGAACG | RT-PCR |
| *Cpsf6*(mouse) | | TCACGGGAAAAGAGTCGTCG | CCTCTTCCTTCAGCTTCTAACGA | RT-PCR |
| *Cdk9*(mouse) | | AGAGACATTCCTGGACGCAG | AGTGTCCTGGCTGACCAAAC | RT-PCR |
| *Mif4gd*(mouse) | | GAGCCTGACGTTTACACCCA | TCCCAGCGGGATCTGAGTTA | RT-PCR |
| *Zbtb17*(mouse) | | AACCCACACTGGCAGAAAGT | CAGGGACCACAGGCAACATT | RT-PCR |
| *Npat*(mouse) | | AGGCTGGCTTTGAACTCATTAC | GCCTGCAATATTGGCACTTAGAG | RT-PCR |
| *Cpsf3*(mouse) | | GCACGTTTACAGCAAGAGGC | TTCTACAGCCCGAGTCTCCA | RT-PCR |
| *Sympk*(mouse) | | AGGAAGTCTTCAACCGCCTG | CTCCGCAAAACACAGGTTGG | RT-PCR |
| *Nelf*(mouse) | | ATGCAAGCGGTATTCAGGGG | GCTTTCATAGGGGTGGGGTC | RT-PCR |
| *Lsm10*(mouse) | | CGGTGAAGGAGCGAACTATTT | CTCGGGCCACACTCTCATC | RT-PCR |
| *U7*(mouse) | | AAGTGTTACAGCTCTTTTAGAATTTGT | AGGGGTTTTCCGACCGAAGTCAGA | RT-PCR |
| *Cdc7(mouse)* | | CGCGGACGTCCTAACTTCTG | GAGTCCCCAAAGAGGCTTCC | RT-PCR |
| *Ccna2(mouse)* | | TTTGGGTTCTTCTCTGGCTCC | GATGGGAGCGTTAGGACCTC | RT-PCR |
| *Mcm5(mouse)* | | AACGAACCAATAGGAGCGCA | CGCGCCACACGAACCT | RT-PCR |
| *SLBP*(human) | | AAGTCCCAAGACACCTTCGAC | TCACATCCTTCTTCCGCTGG | RT-PCR |
| *SRRT*(human) | | CATTGTCAAGATGCTGGATG | CTCATCCTTGTCGTTGGTTT | RT-PCR |
| *CASP8AP2*(human) | | AGTGAGGCTAAAAGTGAAGGTA | TGAACTGGGAGATTCTGTGG | RT-PCR |
| *CSTF2*(human) | | ATCCTTGCCTGCGAATGTCC | GGGTGGTCCTCGGCTCTC | RT-PCR |
| *CPSF2*(human) | | CTTTGGAACCCTTGCCACCT | TGCGGACTGCTACTTGATTGTTG | RT-PCR |
| *LSM11*(human) | | TGGGTGAACTCCATCGCTGTA | GTCGAATGCAACAAGGAAGCC | RT-PCR |
| *CPSF6*(human) | | AAGATTGCCTTCATGGAATTGAG | TCGTGATCTACTATGGTCCCTCTCT | RT-PCR |
| *CDK9*(human) | | AGGAGGGGTTCCCCATTACA | TAGGGGGAAGCTTTGGTTCG | RT-PCR |
| *MIF4GD*(human) | | GCGCATGGATGAGCTCTTTG | CCCTGATCTGGAGGCCTAGT | RT-PCR |
| *ZBTB17*(human) | | GTTCCGCCCGACTCTAACAT | TCATTACCTGCCATGTCCCG | RT-PCR |
| *NPAT*(human) | | AATTGCCTCTCCAGTCCAGC | GAGATTTTGGAGGGACGGGG | RT-PCR |
| *CPSF3*(human) | | AATGGCTGGCAAACCCTTCTAATG | CATCGTCTTCACTTCCCTCTTCACA | RT-PCR |
| *SYMPK*(human) | | GAGATCATCGCATTCCAAGCA | TCACATTCTCGTCCCTCAAGAG | RT-PCR |
| *NELF*(human) | | TACAGCGTTGACCGTGTGTC | CCCTGAACACCGCTTGCATA | RT-PCR |
| *LSM10*(human) | | AAGGAGCGGACCATCTCTGA | TGATGTTCACGTCATCTGGGA | RT-PCR |
| *U7*(human) | | CAGTGTTACAGCTCTTTTAGAATTTG | AGGGGCTTTCCGGTAAAAAGC | RT-PCR |
| *GAPDH (human, mouse)* | | TGCACCACCAACTGCTTAGC | GGCATGGACTGTGGTCATGAG | RT-PCR |
| *Slbp*(mouse) | | GATGCAGTCCAAGATCCCGA | GTATAAGCTAGAGGCGGGCT | ChIP |
| *Cpsf3*(mouse) | | ATAACGTCGCCAAGATGCTC | GCTTTGGAAGCCTGATCTTG | ChIP |
| *SLBP*(human) | CCTGCTCTACTCTGCGCTCT | | GGGACCGCCTTACAACCT | ChIP |
| *CPSF3*(human) | | CGCAGTCCTGACGTCCTAC | AAGGAAGAACCCCATTCACC | ChIP |
| *GFP-ACLY-WT* | | CATAAGCTTATGTCGGCCAAGGCAATTTCA | [TAACCGCGGTTACATGCTCATGTGTTCCGG](javascript:AddMRU('f')) | Cloning |
| *GFP-ACLY-N1* | | CATAAGCTTATGTCGGCCAAGGCAATT | TAACCGCGGCTCGTTGACACCCCC | Cloning |
| *GFP-ACLY-N2* | | CATAAGCTTATGGAGCTGGCAAACTATGGG | TAACCGCGGAGGCTTGATGCCTCCAACAGT | Cloning |
| *GFP-ACLY-N3* | | CATAAGCTTATGAAGCCTGGGTGCTTTAAG | TAACCGCGGCTGTCCTCGCTCATCGCAGAT | Cloning |
| *GFP-ACLY-N4* | | CATAAGCTTATGCAGGAGCTCATCTACGCG | TAACCGCGGTGAAATATCATCCCACGGATG | Cloning |
| *GFP-ACLY H760A* | | GAGGTCCAGTTTGGCGCTGCTGGAGCTTGTGCC | GGCACAAGCTCCAGCAGCGCCAAACTGGACCTC | Cloning |
| *HA-53BP1-N(1-699)* | | TCCCTCGAGCCTGGGGAGCAGATGGACCCT | TGAGGGCCCAGTCAGAGAAAGGTGCAACGGA | Cloning |
| *HA-53BP1 (1-100)* | | TCCCTCGAGCCTGGGGAGCAGATGGACCCT | AGA GGG CCC AGA ATC CAC AGG GTC TGC AAC C | Cloning |
| *HA-53BP1-(1-200)* | | TCCCTCGAGCCTGGGGAGCAGATGGACCCT | AGA GGG CCC GGT GGT TAC TGA TTG TAG | Cloning |
| *HA-53BP1-(1-300)* | | TCCCTCGAGCCTGGGGAGCAGATGGACCCT | GGT GGG CCC TGG TGA CTT CTG AAT CTG CAG TC | Cloning |
| *HA-53BP1-(1-400)* | | TCCCTCGAGCCTGGGGAGCAGATGGACCCT | TAA GGG CCC TTC TTC AGA TAA C AC TGA CG | Cloning |
| *HA-53BP1-(1-500)* | | TCCCTCGAGCCTGGGGAGCAGATGGACCCT | TAA GGG CCC AAT CTC TGA AGT TTT AGA ACA CTC | Cloning |
| *HA-53BP1-(1-600)* | | TCCCTCGAGCCTGGGGAGCAGATGGACCCT | TGA GGG CCC TAA AAT ACT AAT GTC ATC CC | Cloning |
| *HA-53BP1-(101-699)* | | ACC CTC GAG ATG TCT AAC CTT GAC ACA | TGA GGG CCC AGT CAG AGA AAG GTG CAA CGG A | Cloning |
| *HA-53BP1-(201-699)* | | ACC CTC GAG ATG AAC TCT GGT TAT ACC AGG CT | TGA GGG CCC AGT CAG AGA AAG GTG CAA CGG A | Cloning |
| *HA-53BP1-(301-699)* | | AAT CTC GAG ATG CCA GAG CCT GAG GTT | TGA GGG CCC AGT CAG AGA AAG GTG CAA CGG A | Cloning |
| *HA-53BP1-(401-699)* | | AAT CTC GAG ATG TCA ACT CAG GAA GAC TTG TT | TGA GGG CCC AGT CAG AGA AAG GTG CAA CGG A | Cloning |
| *HA-53BP1-(501-699)* | | GCC CTC GAG ATG ATT GAA CCA AAG AAT | TGA GGG CCC AGT CAG AGA AAG GTG CAA CGG A | Cloning |
| *HA-53BP1-(601-699)* | | AAT CTC GAG ATG GCC ACT GGT TGC AAG GGC AG | TGA GGG CCC AGT CAG AGA AAG GTG CAA CGG A | Cloning |
| *HA-53BP1-C* | | ATACTCGAGGATCCCCCCACCACA | CGCAAGGGCCCTTAGTGAGAAACATAATC | Cloning |
| *pGEX-4T-1-53BP1 (699)* | | AATGGATCCATGCCTGGGGAGCAGATGGACCCT | ACCCTCGAGTCAAGTCAGAGAAAGGTGCAACGG | Cloning |
|  | |  |  |  |

**Table S6. List of primer sequences used for detection of histone biogenesis.**

| **Gene** | **Forward primer sequence** | **Reverse primer sequence** | **Used** |
| --- | --- | --- | --- |
| *Hist1h1a*_total(mouse) | TCGGTGGAATCGCAAAGGAG | GTCTCCGACATGGTTGCTGA | RT-PCR |
| *Hist1h1a*_polyA(mouse) | GCCTGGTGGGATTTAGGCAT | ACCACACCTGGAGAGGGTAG | RT-PCR |
| *Hist1h2ac*_total(mouse) | CAACGACGAGGAGCTCAACAAG | TTCTGTTGCTTATTTCCCCTTGG | RT-PCR |
| *Hist1h2ac*_polyA(mouse) | AAGCCTACCACCTACTCCGT | GGAACAACCAAGGAGCTTGC | RT-PCR |
| *Hist1h2bg*_total(mouse) | TGTTTCTACCATGCCCGAGCC | CTTGGTCACGGCCTTCTTGG | RT-PCR |
| *Hist1h2bg*_polyA(mouse) | ACGCTGGGAAATGACACAATA | CGTTGGTTACACTGAGGCAA | RT-PCR |
| *Hist1h2bk*_total(mouse) | TGCTTACCGCGTCCAGACATA | ACACAACTCCCAAAACCTCGT | RT-PCR |
| *Hist1h2bk*_polyA(mouse) | CAGAGTTGCTTGTCTCGGTCA | AGGTATGCCAAACACTCTCCC | RT-PCR |
| *Hist1h3d*_total(mousse) | ACGCGTGTTTAAAATTACTCCTT | TTGTGACCCCAAAACGAACC | RT-PCR |
| *Hist1h3d*_polyA(mouse) | GCAAATGTCGGAATCCTGCC | TCTCTCCAGAGAACTCGCCT | RT-PCR |
| *Hist1h3g*_total(mouse) | CTAGGCACAAGAAGCGGGAA | TTTAGTCTGGCACACCCGAG | RT-PCR |
| *Hist1h3g*_polyA(mouse) | GGTCTCCTTTCATCACACCGA | TGCCCAAATTACTGTAACCATGC | RT-PCR |
| *Hist1h4a*_total(mouse) | TGGCTGAGAGGAGGGATCAG | ACGTCATAAACCCCGCCAAT | RT-PCR |
| *Hist1h4a*_polyA(mouse) | AGTGTGCGCAGCTAAACTCT | ACTTTACTTGAACCCCGCCC | RT-PCR |
| *HISH1H1A*_total(human) | AAGGCAACGGGTGCATCTAA | GATTTCCTTGTTGCCGCAGG | RT-PCR |
| *HISH1H1A*_polyA(human) | TGAACCAAGGTTGAAGCCCA | CCCAAAACGCAATATCCCCAC | RT-PCR |
| *HIST1H2AC*_total(human) | GACGAGGAGCTCAACAAACTG | ACCTGTCAAATCACTTGCCC | RT-PCR |
| *HIST1H2AC*_polyA(human) | CCTGTCCACTGTTGGTAGGC | TTCACTTACCACCATTCCAGC | RT-PCR |
| *HIST1H2AE*_total(human) | AACGACGAGGAGCTAAACAAGT | GAGTTCTCGTTTTACTTGCCC | RT-PCR |
| *HIST1H2AE*_polyA(human) | CGCTTCTGACGTTACTGGTCA | GGTGTCACAACCTCGGTTTCT | RT-PCR |
| *HIST1H2BD*_total(human) | ACGATGCCTGAACCTACCAA | AGCCTTAGTCACCGCCTTCT | RT-PCR |
| *HIST1H2BD*_polyA(human) | CCAACTCATCCTGGTTTGCT | TCCCCTCGGTAACCTTCTTT | RT-PCR |
| *HIST1H2BK*_total(human) | AAGGCCGTCACCAAGTACAC | GCCTTTGGGGTTGGGCTTTA | RT-PCR |
| *HIST1H2BK*_polyA(human) | AAGGCCGTCACCAAGTACAC | AGGCAATTGTGCTTCTTTTGAT | RT-PCR |
| *HIST1H3G*_total(human) | CAAGTTGCCTTTCCAACGCC | CGGAACTCTGAAAGCGCAGA | RT-PCR |
| *HIST1H3G*_polyA(human) | AGGGGTTTGGAGCTTCGATT | CGCGACTAACGCTTGAGACA | RT-PCR |
| *HIST1H4A*_total(human) | TGGAGAACGTGATCCGTGAC | ATCAGCAACCTTAACCGCCA | RT-PCR |
| *HIST1H4A*_polyA(human) | TGGGTAGGGGAGCAACGATA | GATTGGGCAGCGCTTCTTTG | RT-PCR |
| *HIST1H4B*_total(human) | CAAGCGTCACCGAAAAGTGC | CCTTGAGAACGCCACGAGT | RT-PCR |
| *HIST1H4B*_polyA(human) | GGTTGTTTGGTCTGAGTGGC | TCATTTATCGGACTCGCGCT | RT-PCR |

**Table S7. List of siRNA sequences used in this study.**

| **Gene** | **siRNA sequence** |
| --- | --- |
| *si53BP1* | GGACAAGUCUCUCAGCUAU dTdT |
| *si53BP1-3`UTR* | UACUUGGUCUUACUGGUUU dTdT |
| *siACLY* | GGCAUGUCCAACGAGCUCAA dTdT |
| *siSLBP* | GUUCUGAUUCAAAGGAGUCUA dTdT |
| *siNDPK* | CAUUCUUUGCCGGCCUGGUGAAAUA dTdT |
| *Mouse siCdc7* | UUCGAGGUACAGGUCAUAAAG dTdT |
| *Mouse siCcn2* | GCUUCGAAGUUUGAAGAAAUA dTdT |
| *Mouse siMcm5* | UUGUAUGUACAGAGGUAAUAA dTdT |
| Negative control | CCUACGCCACCAAUUUCGU dTdT |

**Table S8. List of antibodies used in this study: WB, ChIP, IP, PLA.**

| **Antibody** | **Species** | **Application** | **Cat. No** | **Suppliers** |
| --- | --- | --- | --- | --- |
| 53BP1 | mouse, human | WB | TA309918 | Origene |
| 53BP1 | human | WB, IF, PLA | 612523 | BD Transduction Laboratories |
| 53BP1 | mouse, human | WB, IP | sc-22760 | Santa cruz |
| β-Actin | mouse, human | WB | SC-47778 | Santa cruz |
| Histone H2A | mouse, human | WB | AB18255 | Abcam |
| Histone H2B | mouse, human | WB | AB1790 | Abcam |
| Histone H3 | mouse, human | WB | AB1791 | Abcam |
| Histone H4 | mouse, human | WB | AB7311 | Abcam |
| Histone Acetyl-H2B(K5,12,15,20) | mouse, human | WB, ChIP | 07-373 | Millpore |
| Histone Acetyl-H3 (N-terminus K9, K14) | mouse, human | WB, ChIP | 06-599 | Millpore |
| Histone Acetyl-H4 (K5,8,12,16) | mouse, human | WB, ChIP | 06-866 | Millpore |
| HA | mouse, human | WB, IP | sc-805 | Santa cruz |
| HA | mouse, human | WB, IP | sc-7392 | Santa cruz |
| SLBP | human | WB | AB181972 | Abcam |
| SLBP | mouse | WB | SC-390833 | Santa cruz |
| CPSF3 | mouse, human | WB | A301-091A | Bethyl |
| Flag | mouse, human | WB | F7425 | Sigma-Aldrich |
| p53 | human | WB | SC-126 | Santa cruz |
| GFP | mouse, human | WB, IP | SC-8334 | Santa cruz |
| GFP | mouse, human | WB, IP | sc-9996 | Santa cruz |
| GFP | mouse, human | WB, IP | NB600-308 | NOVUS Biologicals |
| α-Tubulin | mouse, human | WB | SC-8035 | Santa cruz |
| ACLY | mouse, human | WB | #4332 | Cell Signaling |
| ACLY | mouse, human | WB, IP,PLA | ab157098 | abcam |
| ACLY | mouse, human | WB, IP | NB110-55476 | NOVUS Biologicals |
| Phosphohistidine (3-pHis) | mouse, human | WB, IP | MABS1352 | Millpore |
| NDPK | mouse, human | WB, IP | sc-166937 | Santa cruz |
| NDPK | mouse, human | WB, IP | NBP2-19549 | NOVUS Biologicals |
| Acetyl-Histone H4 Antibody Sampler Kit | mouse, human | WB,Chip | #8346 | cell signaling |
| GST(B-14) | mouse, human | WB | sc-138 | Santa cruz |
| Anti-BrdU (B44) | mouse, human | Fiber assay | 347580 | BD Biosciences |
| Anti-BrdU (BUI/75(ICR1)) | mouse, human | Fiber assay | ab6326 | abcam |
| Alexa Fluor® 594 Donkey Anti-Rat IgG (H+L) | mouse, human | Fiber assay | A-21442 | Invitrogen |
| Alexa Fluor® 488 chicken anti-mouse IgG (H+L) *2 mg⁄mL* | mouse, human | Fiber assay | A-21200 | Invitrogen |
| Fluorescent mounting medium with DAPI | mouse, human | PLA assay, Chromosomal aberration analysis | H1200-10 | Vector Laboratories |
| Peroxidase-conjugated Affinipure Goat Anti-mouse IgG Fcg fragment specific | mouse, human | WB, IP | 115-035-008 | Jackson |
| Peroxidase-conjugated Affinipure Goat Anti-rabbit IgG Fc fragment specific | mouse, human | WB, IP | 111-035-008 | Jackson |
| IgG from mouse serum | mouse, human | IP | I5381 | Sigma-Aldrich |
| IgG from Rabbit serum | mouse, human | IP | I5006 | Sigma-Aldrich |
